# Synthesis of Quinoline-Based Pt–Sb Complexes with L- or Z-Type Interaction: Ligand-Controlled Redox via Anion Transfer

**DOI:** 10.1021/acs.organomet.4c00221

**Published:** 2024-08-13

**Authors:** Christopher K. Webber, Fanji Kong, Jugal Kumawat, Jyothish Joy, Erica K. Richardson, Paolo Siano, Diane A. Dickie, Daniel H. Ess, T. Brent Gunnoe

**Affiliations:** † Department of Chemistry, University of Virginia, Charlottesville, Virginia 22904, United States; ‡ Department of Chemistry and Biochemistry, Brigham Young University, Provo, Utah 84604, United States

## Abstract

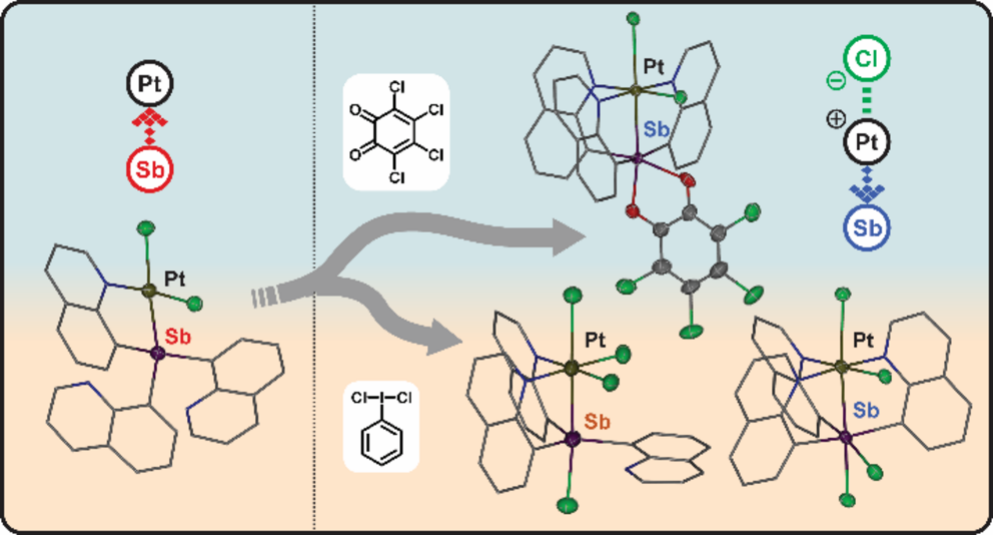

A series of Pt–Sb complexes with two or three L-type quinoline side arms were prepared and studied. Two ligands, tri(8-quinolinyl)stibane (SbQ_3_, Q = 8-quinolinyl, **1**) and 8,8′-(phenylstibanediyl)diquinoline (SbQ_2_Ph, **2**), were used to synthesize the Pt^II^–Sb^III^ complexes (SbQ_3_)PtCl_2_ (**3**) and (SbQ_2_Ph)PtCl_2_ (**4**). Chloride abstraction with AgOAc provided the bis-acetate complexes (SbQ_3_)Pt(OAc)_2_ (**5**) and (SbQ_2_Ph)Pt(OAc)_2_ (**6**). To better understand the electronic effects of the Sb moiety, analogous bis-chloride complexes were oxidized to an overall formal oxidation state of +7 (i.e., Pt + Sb formal oxidation states = 7) using dichloro(phenyl)-λ^3^-iodane (PhICl_2_) and 3,4,5,6-tetrachloro-1,2-dibenzoquinone (*o*-chloranil) as two-electron oxidants. Depending on the oxidant, different conformational changes occur within the coordination sphere of Pt as confirmed by single-crystal X-ray diffraction and NMR spectroscopy. In addition, the nature of Pt–Sb interactions was evaluated via molecular and localized orbital calculations.

## Introduction

**Figure 1:**
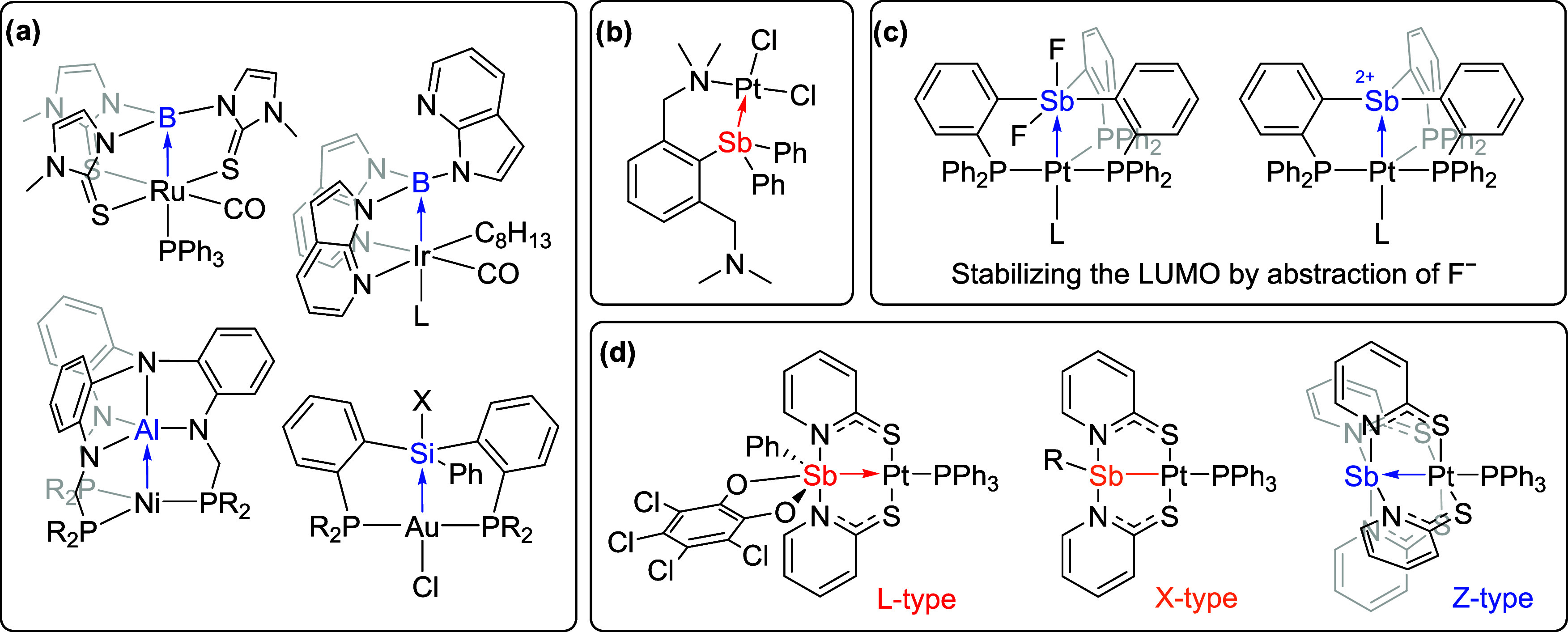
(a) Selected examples of transition metal complexes with Z-type ligands. (b) Previously reported Pt^II^–Sb^III^ complex (L-type Sb ligand) supported by nitrogen-based side arms. (c) Examples of tunable Lewis acidity of Pt–Sb complexes. (d) Pt–Sb complexes exhibiting a variety of bonding types.^36−39,41−43^

Recently, the potential of multimetallic (or metalloid) metal complexes in homogeneous catalysis has been demonstrated.^1−16^ One example is the use of Z-type ligands that form direct bonds with transition metals to modulate reactivity (Figure 1).^17−43^ Based on the covalent bond classification (CBC) method published by Green, in general, the 2-center-2-electron bonding interactions between the metal center and the ligand can be classified into three types, which are known as the L-type, X-type, and Z-type interactions.^44^ Ligands of L-type are considered neutral two-electron donors to the metal center, and X-type ligands are defined as forming a bond with one electron from the metal center and one electron from the ligand to form a covalent bond.^44^ Z-type ligands are defined to form bonds with a metal through the donation of an electron pair from the metal center to an empty ligand orbital.^44,45^ In this article, we specifically discuss the Z-type interactions in σ-symmetry, for which a Z-type ligand is referred to as a σ-acceptor ligand. Among examples of Z-type ligands, side-arm ligands with P and S donors are common auxiliary chelating functional groups used to position the Z-type moiety.^19^ Compared to these, *N*-based donors, such as quinoline and amines, appear to be less common; however, examples of *cis*-bidentate ligands with one amine/quinoline arm that position the L-type Sb^III^ center within the coordination sphere of ligated metals have been reported (Figure 1b).^40,41,46^

Previously, examples of Sb-based ligands with redox and coordination non-innocent features have been demonstrated.^17,47,48^ Among these, the Gabbaï group had studied Pt–Sb complexes with phosphine auxiliary donors, which they have shown to catalyze electrophilic transformations and photo-driven reductive elimination of chlorine.^42,48−51^ In addition, their studies have demonstrated that by abstracting the halides from Sb^V^, the lowest unoccupied molecular orbital (LUMO) on Sb can be stabilized, which results in an increase in Lewis acidity of the Sb center that leads to differences in the catalytic activity of Pt complexes (Figure 1c).^42^ The Wagler group has utilized Mössbauer spectroscopy coupled with DFT calculations to fully characterize and assign the full spectrum of L-, X- and Z-type bonding interactions between Pt and Sb with pyridine-2-thiolate supporting ligands (Figure 1d).^43^

**Figure 2:**
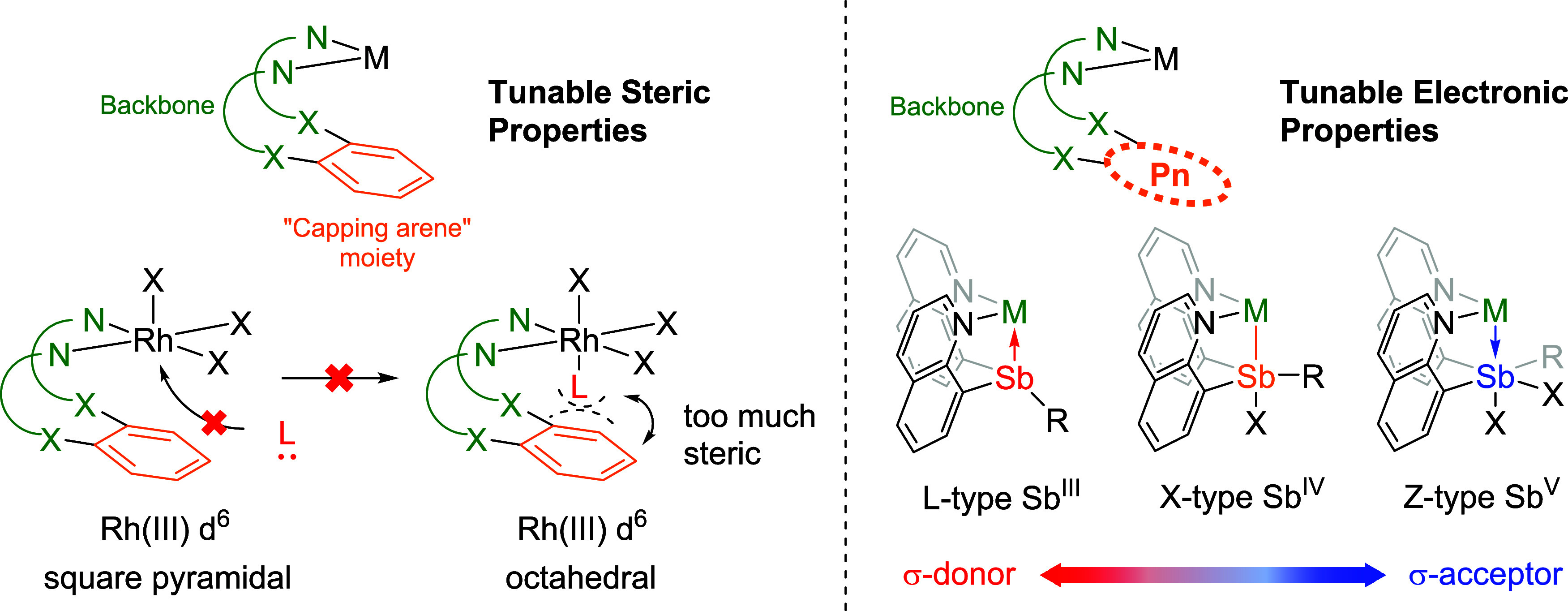
Design of “capping arene” ligands with tunable steric properties (left). The design of Sb-based ligands offers potential redox and coordination flexibility (right). Pn = Pnictogen.

A series of “capping arene” ligands that offer tunable steric properties have been studied previously.^52−60^ The design of this ligand structure utilizes L-type *N*-based donor groups to position a “capping arene” moiety near a transition metal center, and depending on the metal center and oxidation state, the arene group can potentially donate (via donation of arene π-electrons) and/or remove electron density (through metal-to-arene back bonding into π* orbitals).^61−64^ By synthesizing different capping arene motifs, M–arene distances can be controlled and thus offer tunable steric bulk and metal–arene interactions (Figure 2, left). For example, we reported the influence of the capping arene ligand structure on the rate of reductive elimination from Rh^III^,^53^ Rh-catalyzed olefin hydrogenation,^55^ and Co-catalyzed water oxidation.^57^

Building on the “capping arene” structure and the notion of tuning reactivity with the donor/withdrawing ability of the arene group, we found an analogy to Sb^III^/Sb^V^ donor (L-type) or acceptor (Z-type) motifs (Figure 2, right). Antimony was selected for our studies due to its strong Lewis acidity (based on the fluoride ion affinity)^65,66^ and tunable σ-donation/accepting ability (varied by control of the Sb oxidation state, e.g., Sb^III^ vs Sb^V^).

Complexes with a Pt–Sb bond have been reported.^42,48−50,67^ Herein, we report a series of Pt–Sb complexes with the ligands bonding in bi-, tri-, and tetradentate coordination modes in which the Sb center is in +3 to +5 formal oxidation states.

## Results and Discussion

### Synthesis and Characterization of Sb^III^ Ligands

**Scheme 1:**
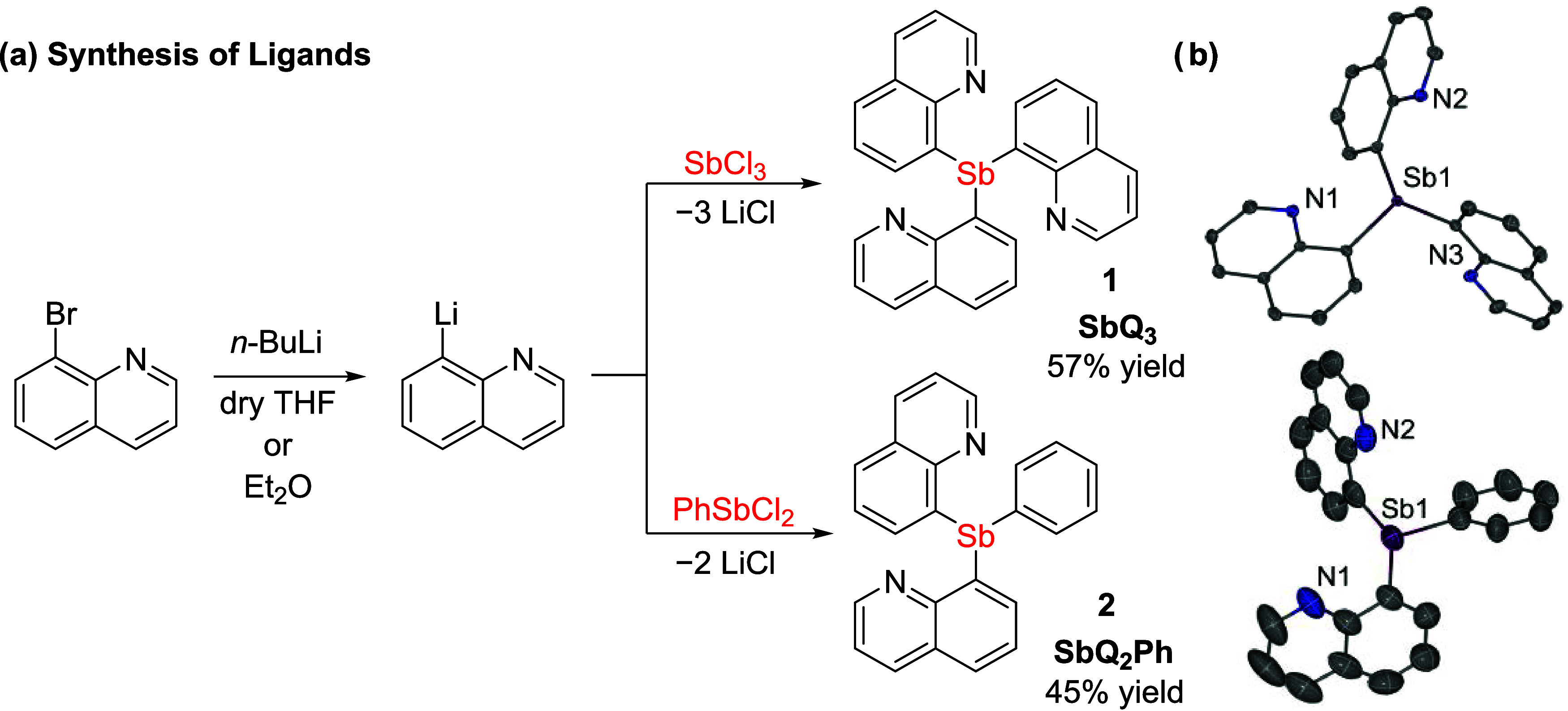
(a) Synthesis of SbQ_3_ (**1**) and SbQ_2_Ph (**2**) (Q = 8-quinolinyl) and (b) ORTEPs of **1** and **2** Ellipsoids are drawn at the 50% probability level and hydrogen atoms and non-coordinating solvents are omitted for clarity. THF = tetrahydrofuran; Et_2_O = diethyl ether.

Two quinoline-appended Sb^III^ ligands with a variable number of *N*-donor side arms, tri(quinolin-8-yl)-λ^3^-stibane (SbQ_3_, **1**) and di(quinolin-8-yl)-phenyl-λ^3^-stibane (SbQ_2_Ph, **2**), were synthesized (Scheme 1a). The proligands **1** and **2** offer potential tetradentate and tridentate coordination, respectively. The use of quinoline-based Sb ligands has been explored previously by the Wright group who utilized 2-methylquinoline Sb ligands to bind various coinage metals including Ag^I^, Au^I^, and Cu^I^.^68^ Compound **1** was isolated in multigram scale with an isolated yield of 57% from the treatment of SbCl_3_ with three equivalents of 8-lithio-quinoline. Compound **2** was synthesized in a similar manner using SbPhCl_2_ and two equivalents of 8-lithio-quinoline with a 45% isolated yield.

Both proligands exhibited stability in the presence of air and moisture (a characteristic also indicated by quinoline-appended Sb ligands from the Wright group)^68^ and could be washed with water quickly with no significant impurity formation. The solid-state structures of compounds **1** and **2** indicated trigonal pyramidal geometries, which is consistent with the presence of a Sb^III^ center (Scheme 1b). Furthermore, the approximate 95° C–Sb–C bond angles of **1** and **2** (range of C–Sb–C bond angles, for **1**: 94.10(7)–97.34(7)°; for **2**: C–Sb–C 92.9(3)–95.8(3)°) are in agreement with the previously reported similar Sb quinoline ligands. The N atoms of the quinoline side arms are oriented toward the lone-pair of Sb with Sb···N contacts with relatively short distances ranging from 3.0316(17) to 3.0989(16) Å for **1** and from 3.090(8) to 3.103(10) Å for **2**, which is likely due to the sterics between quinoline rings in the solid-state structures. Similar observations have been reported for Sb and Bi compounds.^68^

### Synthesis of Pt–Sb Complexes

**Scheme 2:**
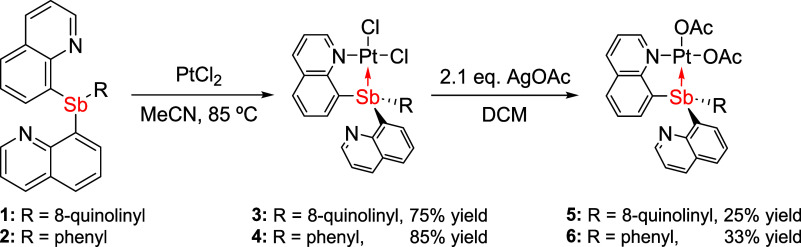
Synthetic Routes for Pt–Sb Complexes (SbQ_3_)PtCl_2_ (**3**), (SbQ_2_Ph)PtCl_2_ (**4**), (SbQ_3_)Pt(OAc)_2_ (**5**), and (SbQ_2_Ph)Pt(OAc)_2_ (**6**) from SbQ_3_ (**1**) and SbQ_2_Ph (**2**) (OAc = acetate)

The L-type Pt complexes (SbQ_3_)PtCl_2_ (**3**) and (SbQ_2_Ph)PtCl_2_ (**4**) were synthesized by reacting PtCl_2_ with one equivalent of **1** or **2**, respectively, in MeCN at 85 °C with isolated yields of 75 and 85%, respectively (Scheme 2).

**Figure 3:**
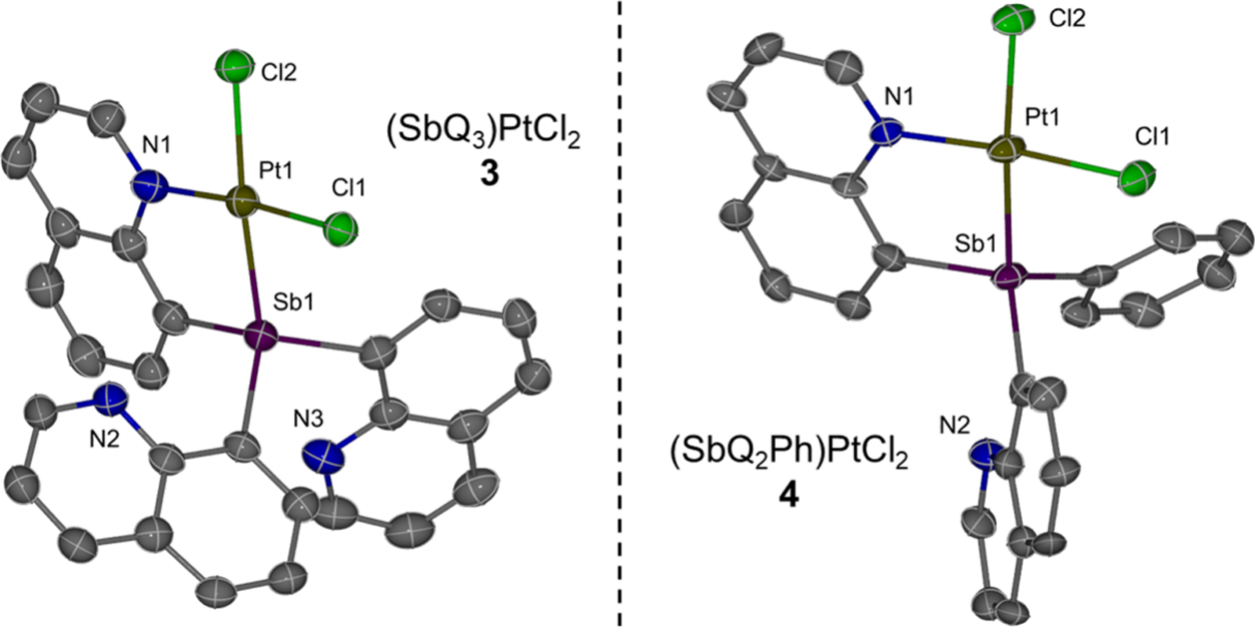
ORTEPs of (SbQ_3_)PtCl_2_ (**3**) and (SbQ_2_Ph)PtCl_2_ (**4**). Ellipsoids are drawn at 50% probability level, and hydrogen atoms and non-coordinating solvents are omitted for clarity.

The solid-state structures of **3** and **4** were determined by single-crystal X-ray diffraction (Figure 3). The Pt–Sb bond distances for **3** and **4** are 2.4556(4) and 2.4500(8) Å, respectively, which are shorter than the sum of Sb and Pt covalent radii (i.e., 2.75 Å). Thus, the Pt–Sb distances are consistent with a bonding interaction. The Pt–Cl bond length of the chloride trans to Sb is slightly longer than that of the *cis*-chloride with distances of 2.3818(14) versus 2.2962(13) Å for complex **3** and 2.370(2) versus 2.297(2) Å for complex **4** (Table 1). For complexes **3** and **4**, both ligands coordinate to the Pt center in a bidentate fashion, with Pt bound to Sb and the nitrogen atom from one of the quinoline side arms, while the other quinoline side arm(s) is uncoordinated. This results in a square planar geometry for the Pt center, which is commonly observed for L-type bidentate pnictogen Pt^II^ complexes.^69^

**Table 1:** Selected Bond Lengths for (SbQ_3_)PtCl_2_ (**3**) and (SbQ_2_Ph)PtCl_2_ (**4**)

		bond distance (Å)	
entry	bond	**3**	**4**
1	Pt1–Sb1	2.4556(4)	2.4500(8)
2	Pt1–Cl1	2.2962(13)	2.297(2)
3	Pt1–Cl2	2.3818(14)	2.370(2)
4	Pt1–N1	2.073(5)	2.081(7)
5^a^	H_a_···Cl2	2.4029(14)	2.4606(19)

a H_a_ is based on the calculated position of the quinoline proton *ortho* to the nitrogen.

The ^1^H NMR spectrum of complex **3** shows two groups of proton resonances attributed to the quinolines with an approximate 2-to-1 integrated ratio. This observation is consistent with the solid-state structure, which includes one coordinated quinoline side arm and two chemically equivalent uncoordinated quinoline groups. The proton resonances belonging to the coordinated quinoline are shifted downfield, while the chemical shifts of the uncoordinated quinoline groups are similar to the free ligand. The ^1^H NMR spectrum of **4** is consistent with the X-ray structure that includes an uncoordinated quinoline side arm.

**Figure 4:**
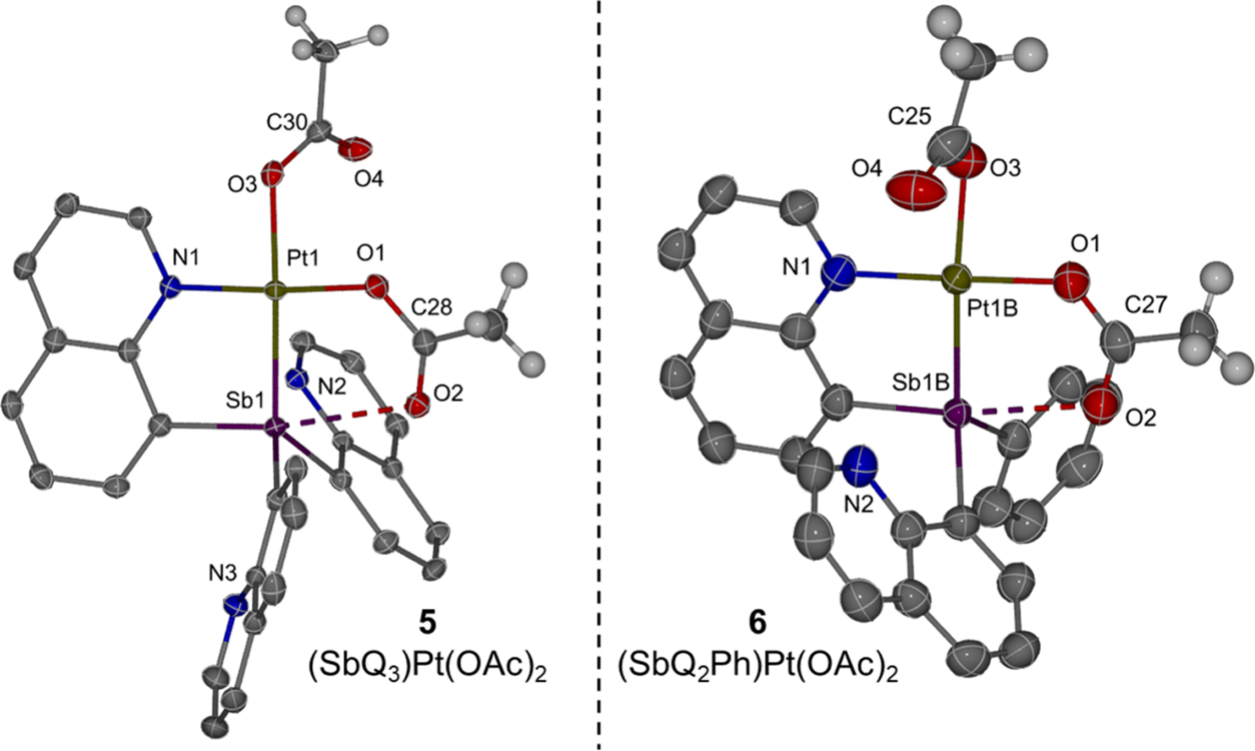
ORTEPs of (SbQ_3_)Pt(OAc)_2_ (**5**) and (SbQ_2_Ph)Pt(OAc)_2_ (**6**). Ellipsoids are drawn at the 50% probability level. Some of the hydrogen atoms, the non-coordinating solvents, and the minor position of the disordered atoms (**6**) are omitted for clarity.

The Pt carboxylate complexes (SbQ_3_)Pt(OAc)_2_ (**5**) and (SbQ_2_Ph)Pt(OAc)_2_ (**6**) were synthesized by the treatment of complex **3** or **4**, respectively, with 2.1 equiv of AgOAc in dichloromethane (DCM) at room temperature (Scheme 2). The X-ray structures of both **5** and **6** indicate similar acetate binding modes except for an apparent interaction between one acetate bridging Pt and Sb in **6** (Figure 4). Complex **6** has disordered Pt–Sb atoms, resulting in two separate bond distances in the structure. In complexes **5** and **6**, the lengths of the Pt–O bonds trans to the Sb center are 2.1104(14) and 2.141(5)/2.103(3) Å, respectively. In contrast, the acetate ligands cis to Sb have slightly shorter Pt–O bond distances ranging from 1.979(5) to 2.0037(14) Å, which suggests a stronger trans influence of Sb compared to quinoline. The distance between the Sb and O2 atom is 2.6948(14) Å for **5** and ranged from 2.457(7) to 2.531(3) Å for **6** (Table 2), and both Sb1···O2 distances are longer than the sum of Sb and oxygen covalent radii (i.e., 2.05 Å). The distinct bond lengths in the crystal structures between O1–C and O2–C (Table 2) suggest that there is little resonance delocalization across the acetate ligand. We speculate that the acetate ligand can transfer between Pt and Sb centers via the formation of a paddlewheel-type structure. Similar examples have been reported before with bridging pyS ligands.^43^

**Table 2:** Selected Bond Lengths for Complex (SbQ_3_)Pt(OAc)_2_ (**5**) and (SbQ_2_Ph)Pt(OAc)_2_ (**6**) Based on Single-Crystal X-ray Structures

		bond distances (Å)	
entry	bond	**5**	**6**
1	Pt1–Sb1	2.45013(15)	2.423(7)/2.4529(9)
2	Pt1–O1	2.0037(14)	1.979(5)/2.015(3)
3	Pt1–O3	2.1104(14)	2.141(5)/2.103(3)
4	Pt1–N1	2.0263(16)	2.078(5)/2.037(4)
5	Sb1···O2	2.6948(14)	2.457(7)/2.531(3)
6	O1–C	1.292(3)	1.275(6)
7	O2–C	1.235(3)	1.239(6)

**Figure 5:**
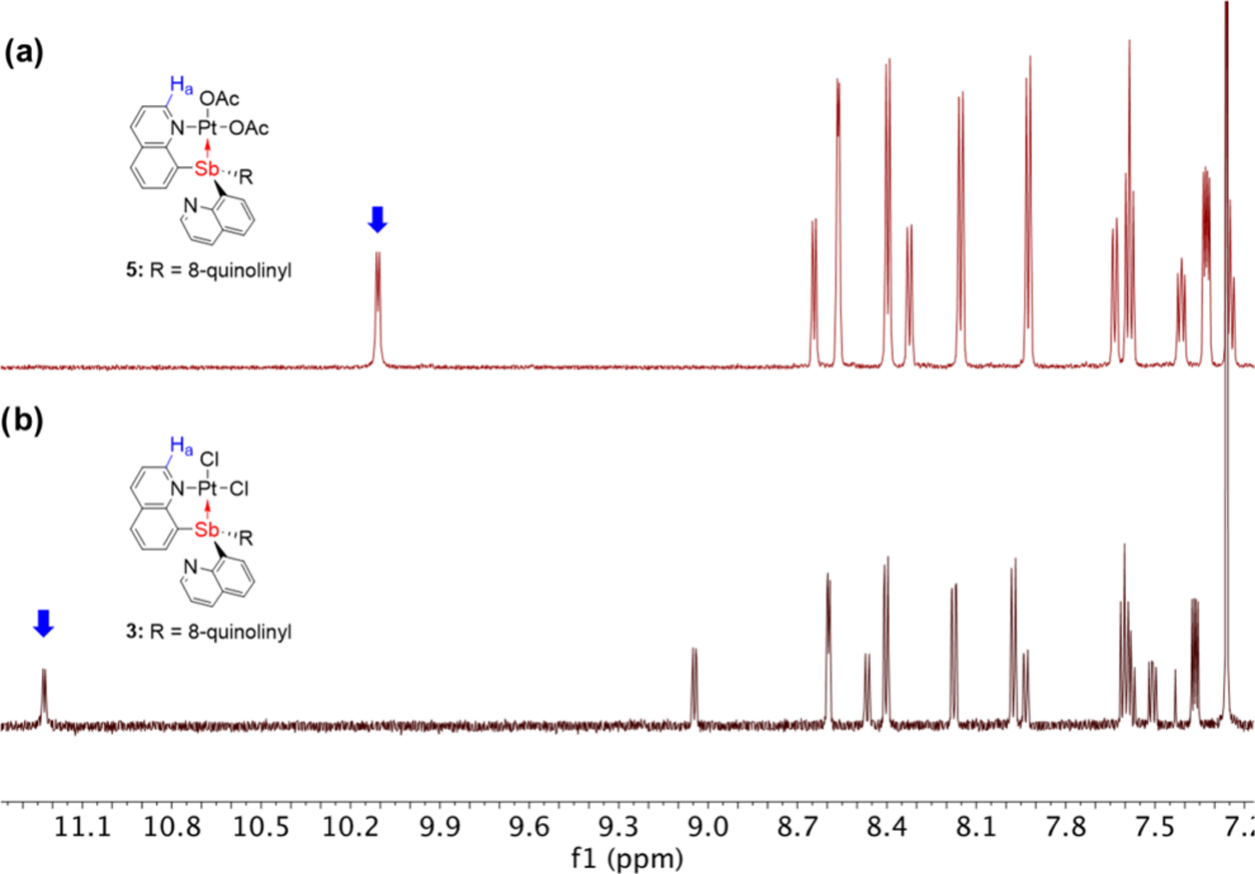
Representative ^1^H NMR spectra of (a) (SbQ_3_)Pt(OAc)_2_ (**5**) and (b) (SbQ_3_)PtCl_2_ (**3**) in CDCl_3_ demonstrating the upfield shift of ^1^H NMR peaks when acetate is substituted for chloride.

In the ^1^H NMR spectra of complexes **5** and **6**, the most downfield-shifted resonance, assigned as the quinoline proton *ortho* to the nitrogen (H_a_), has a chemical shift of ∼10 ppm. However, in complexes **3** and **4**, the corresponding proton resonances exhibit an uncommon downfield chemical shift >11 ppm, as shown in Figure 5 labeled with a blue arrow (complex **3** vs **5**). The downfield shifts for **3** and **4** are likely attributable to the close through-space distance between H_a_ and Cl2 on Pt that is trans to the Sb center (Table 1). In the ^1^H NMR spectra of **5** and **6**, two proton resonances have been observed for the coordinated acetate (2.26 and 1.96 ppm for **5**; 2.37 and 2.10 ppm for **6**). In addition, EXSY experiments have been completed, and the data are consistent with no exchange between the two coordinated acetates (Supporting Information, Section 8).

### Reactions with Chemical Oxidants

**Scheme 3:**
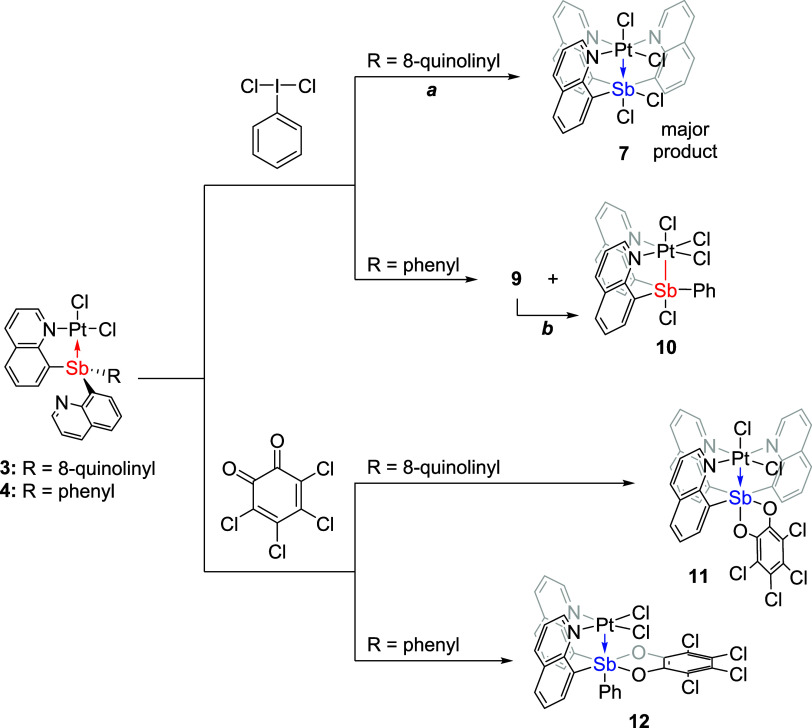
Overview of the Synthetic Routes for Complexes (Cl_2_SbQ_3_)PtCl_2_ (**7**), (ClSbQ_2_Ph)PtCl_3_ (**10**), {(*o*-Chloranil)SbQ_3_}PtCl_2_ (**11**), and {(*o*-Chloranil)SbQ_2_Ph}PtCl_2_ (**12**) via the Oxidation of (SbQ_3_)PtCl_2_ (**3**) or (SbQ_2_Ph)PtCl_2_ (**4**) Using PhICl_2_ or *o*-Chloranil (a) (ClSbQ_3_)PtCl_3_ (**8**) was also observed from the reaction, see Supporting Information, Section 2. (b) An intermediate, (Cl_2_SbQ_2_Ph)PtCl_2_ (**9**), with an unconfirmed structure was observed in the reaction. This intermediate converts to complex **10** over time.

To obtain Pt → Sb complexes with a formal Sb^V^ center, iodobenzene dichloride (**PhICl**_**2**_) and 3,4,5,6-tetrachloro-1,2-dibenzoquinone (***o*****-chloranil**) were tested as oxidants with complexes **3** and **4** (Scheme 3). Using PhICl_2_ as the oxidant with either complex **3** or **4** generated a mixture of products due to the redox of both the Pt and Sb centers in which the Sb center served as either a Z-, X-, or L-type ligand; in contrast, the chelating oxidant, *o*-chloranil, led to the formation of single products with selective oxidation of solely the Sb center (see below).

**Figure 6:**
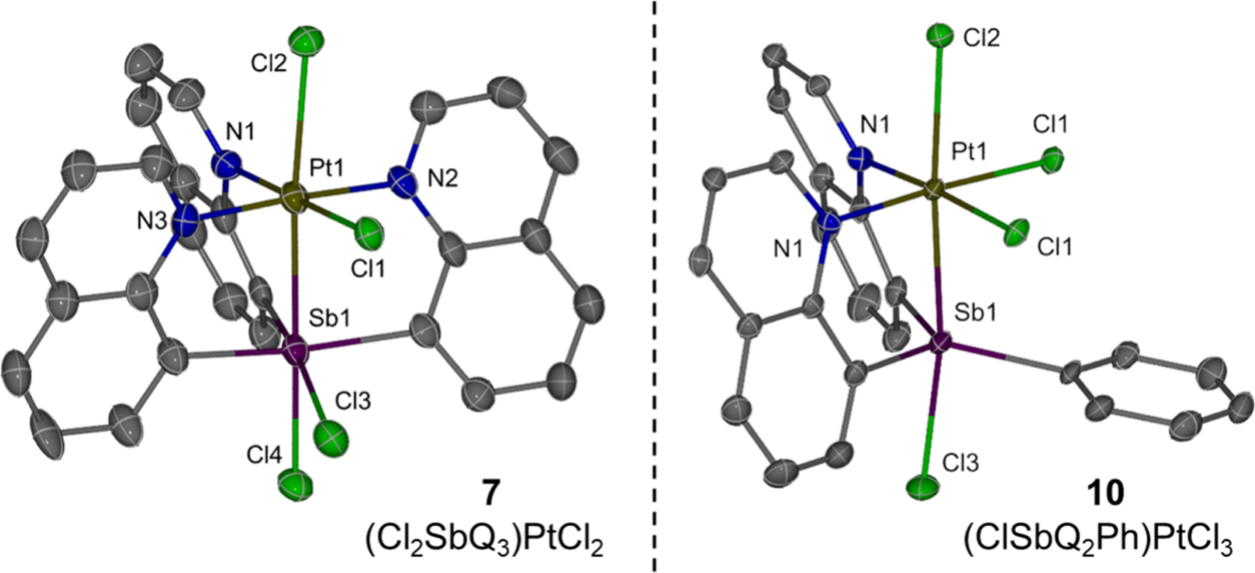
ORTEPs of (Cl_2_SbQ_3_)PtCl_2_ (**7**) and (ClSbQ_2_Ph)PtCl_3_ (**10**). Ellipsoids are drawn at the 50% probability level and hydrogen atoms and non-coordinating solvents are omitted for clarity.

Reacting complex **3** with PhICl_2_ in DCM or chloroform at room temperature resulted in the formation of a white solid precipitate, Cl_2_SbQ_3_PtCl_2_ (**7**), as the major isolated product. During the in situ ^1^H NMR studies, an intermediate, likely ClSbQ_3_PtCl_3_ (**8**), was observed. This complex converts to complex **7** (see Supporting Information, Section 2). Complex **7** has poor solubility in most NMR solvents, but with dimethyl sulfoxide (DMSO) it results in the conversion to a different complex likely due to the solvent (i.e., DMSO) coordination. By using CDCl_3_, in which complex **7** is slightly soluble, a ^1^H NMR spectrum of **7** was obtained; however, a small amount of complex **8** remains in the solution as an impurity. The solid-state structures of complexes **7** (Figure 6, left) and **8** (Figure S2) have been confirmed by X-ray crystallography. The observed ^1^H NMR spectrum of complex **7** is consistent with the solid-state crystal structure in which two groups of quinoline proton resonances are observed with a 2-to-1 integration ratio.

Similar to complex **3**, the oxidation of complex **4** using PhICl_2_ results in two products based on ^1^H NMR spectroscopy (Scheme 3). One of the formed products, which we propose is (Cl_2_SbQ_2_Ph)PtCl_2_ (**9**), is soluble in CDCl_3_, while the other product, (ClSbQ_2_Ph)PtCl_3_ (**10**), has poor solubility and forms a white precipitate during the reaction in DCM or chloroform. The crystal structure of complex **10** (Figure 6, right) shows a coordination mode similar to that of complex **8** for which three chlorides coordinate to the Pt center and one chloride coordinates to the Sb center. The observed ^1^H NMR spectrum of complex **10** is consistent with the solid-state crystal structure in which only one group of quinoline proton resonances is observed due to symmetry. Although we were unable to characterize **9** with single-crystal X-ray diffraction, due to the conversion from **9** to **10**, we propose that **9** has two chlorides bonded to Sb and two are coordinated to Pt (see Supporting Information, Section 3). The ^1^H NMR spectrum of complex **9** shows one group of quinoline proton resonances that suggest the phenyl ring is likely trans to the Pt center. In addition, the presence of a more downfield-shifted proton in complex **10** (11.5 ppm) than in **9** (11.0 ppm) in CDCl_3_ (Figure S6) is consistent with the proposed structure of **9** without an axial chloride trans to the Sb center. The conversion from complex **9** to **10** is potentially initiated by the chloride transfer from the Sb to Pt center, while the conversion from **8** to **7** likely involves a chloride transfer from Pt to Sb. To better understand this difference in coordination mode, the relative energies of complex **7** versus **8** and complex **9** versus **10** were examined via DFT calculations, which suggests that the isolated products (**7** and **10**) were relatively more stable in energy (see Supporting Information, Section 4).

For complex **10**, the Sb center is bonded to only one chloride and three carbon atoms, which gives formal Pt^IV^ and Sb^III^ or Pt^III^ and Sb^IV^ oxidation states (if Sb–Cl is considered as a formal bond). Complexes **7** and **10** each have much longer Pt–Cl distances trans to Sb (2.6842(15) and 2.6164(18) Å, respectively, Table 3) compared to complexes **3** or **4** (2.3818(14) and 2.370(2) Å, Table 1), which are longer than the sum of covalent radii of Pt and Cl (i.e., 2.38 Å). In contrast, the Pt–Cl bond cis to Sb is similar between the oxidized complexes (2.3189(15) Å for **7** and 2.3094(12) Å for **10**) and the Sb^III^ complexes (2.2962(13) Å for **3** and 2.297(2) Å for **4**). These distances are consistent with the chloride trans to Sb more closely resembling a non-coordinating anion. Anion transfers between Sb-bound metals have been explored previously, and tuning the electronic nature of Sb has been reported to elicit changes in coordination modes.^70,71^

**Table 3:** Selected Bond Lengths for Complexes (Cl_2_SbQ_3_)PtCl_2_ (**7**) and (ClSbQ_2_Ph)PtCl_3_ (**10**) Based on Single-Crystal X-ray Structures

		bond distances (Å)	
entry	bond	**7**	**10**
1	Pt1–Sb	2.6316(5)	2.5941(6)
2	Pt1–Cl1	2.3189(15)	2.3094(12)
3	Pt1–Cl2	2.6842(15)	2.6164(18)
4	Pt1–N1	2.044(5)	2.048(4)
5^a^	Sb1–Cl_A_	2.4469(16)	2.493(2)
6^b^	Sb1–Cl_B_	2.5569(17)	N/A
7^c^	H_a_···Cl2	2.5939(16)/2.5400(16)/2.5308(16)	2.6232(16)

a Sb–Cl_A_ is trans to Pt.

b Sb–Cl_B_ is cis to Pt.

c H_a_ is the calculated position of the quinoline proton *ortho* to the nitrogen.

To address the chloride transfer between Pt and Sb, *o*-chloranil was selected as the oxidant due to its bidentate chelating nature, which could kinetically inhibit transfer from Sb to the Pt center. Stirring either **3** or **4** in DCM with 1.1 equivalents of *o*-chloranil led to a single Pt^II^–Sb^V^ product (complexes **11** and **12**, respectively) with *o*-chloranil coordinated to the Sb center (Scheme 3). Similar to complex **7**, the ^1^H NMR spectrum of complex **11** includes two groups of quinoline proton resonances with a 2-to-1 integration ratio as well as of two downfield-shifted resonances due to H_a_ (>12 ppm), which both suggest that the Sb ligand coordinated to Pt in a tetradentate fashion.

**Figure 7:**
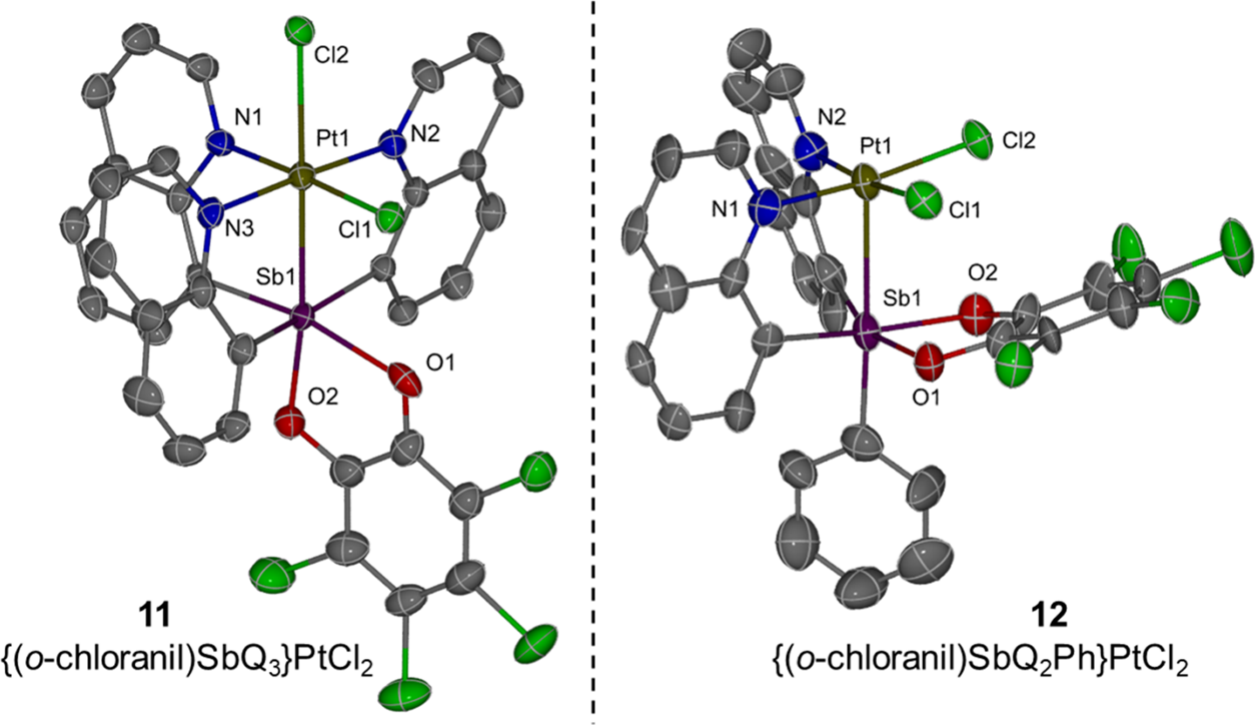
ORTEPs of {(*o*-chloranil)SbQ_3_}PtCl_2_ (**11**) and {(*o*-chloranil)SbQ_2_Ph}PtCl_2_ (**12**). Ellipsoids are drawn at the 50% probability level, and hydrogen atoms and non-coordinating solvents are omitted for clarity.

The crystal structure of complex **11** shows octahedral geometries for both Pt and Sb (Figure 7, left). Based on the structures, Sb can be assigned a formal oxidation state of +5 with bonding to the Pt^II^ center as a Z-type ligand. However, transition metal complexes with *d*(8) configuration (e.g., Pt^II^) normally favor a square planar geometry to minimize the energy. Similar to complex **7**, in complex **11**, the chloride trans to Sb might be better described as a counterion given the long Pt–Cl bond relative to the cis chloride (2.7792(14) vs 2.2938(14) Å, Table 4) as well as the sum of covalent radii of Pt and Cl (i.e., 2.38 Å). Therefore, the Pt^II^ center could potentially be considered to be in a square planar geometry with three quinoline and one chloride donor or in a square pyramidal geometry if the Pt–Sb interaction is considered, while the Sb serves as a σ-acceptor that increases the Lewis acidity of the Pt center thus causing a close Pt contact with the chloride counterion in a position trans to the Sb center. However, in the crystal structure of complex **11**, the Sb1–O1 bond is longer than Sb1–O2 (2.208(5) vs 2.048(4) Å, Table 4), while O1–C is shorter than a typical O–C single bond and different from O2–C (1.268(8) vs 1.332(8) Å, Table 4). This suggests that a covalent bonding interaction may not be the best way to describe the interaction between Sb1 and O1 atoms and, thus, raises the question about the oxidation states of Pt and Sb in complex **11** (Pt^II^–Sb^V^ vs Pt^III^–Sb^IV^, see below for more discussion).

**Table 4:** Selected Bond Lengths for Complexes {(*o*-Chloranil)SbQ_3_}PtCl_2_ (**11**) and {(*o*-Chloranil)SbQ_2_Ph}PtCl_2_ (**12**) Based on Single-Crystal X-ray Structures

		bond distances (Å)	
entry	bond	**11**	**12**
1	Pt1–Sb1	2.6245(4)	2.8375(12)
2	Pt1–Cl1	2.2938(14)	2.287(3)
3	Pt1–Cl2	2.7792(14)	2.295(3)
4	Sb1–O1	2.208(5)	2.061(10)
5	Sb1–O2	2.048(4)	2.062(10)
6	O1–C	1.268(8)	1.343(11)
7	O2–C	1.332(8)	1.333(12)
8^a^	H_a_···Cl2	2.5039(15)/2.5145(15)/2.6016(14)	NA

a H_a_ is the calculated position of the quinoline proton *ortho* to the nitrogen.

The ^1^H NMR spectrum of complex **12** is consistent with the solid-state structure (Figure 7, right) for which the two quinoline groups are equivalent due to symmetry. The Pt center in complex **12** shows a square pyramidal geometry, while the Sb center is in an octahedral geometry. Different from complex **11**, the two Sb–O bonds in complex **12** have similar bond lengths (∼2.06(1) Å, Table 4) as well as two O–C bonds (1.343(11) and 1.333(12) Å, Table 4). Furthermore, the Pt–Sb bond length of 2.8375(12) Å (Table 4) is longer than the sum of covalent radii (i.e., 2.75 Å), which likely indicates only a weak interaction between Pt and Sb. Based on these observations, it seems reasonable to conclude that complex **12** can be considered as a “square planar” Pt^II^ complex with two quinoline and two chloride donor ligands, while the Sb^V^ moiety serves as a Z-type ligand withdrawing electron density from the Pt center through a σ-interaction. We speculate that the geometrical differences between complexes **12** and **11** or **7** are caused by the lack of the third quinoline side arm.

### Pt–Sb Bonding Analysis

With the synthesis of several unique Pt–Sb complexes, we wanted to understand the bonding between the Pt and Sb centers. To do this, we examined both molecular (MO) and natural localized (NLMO) orbitals, which provide the ability to differentiate the X-, L-, and Z-type bonding scenarios. We were most interested in complexes **7** and **11** because they have a unique formal Pt^II^ coordination environment and geometry in which the Pt–Cl distance for the axial chloride connected to the Pt was found to be much longer than that for the equatorial chloride. Also, the absence of the axial chloride in **12** presented a much longer Pt–Sb distance.

**Figure 8:**
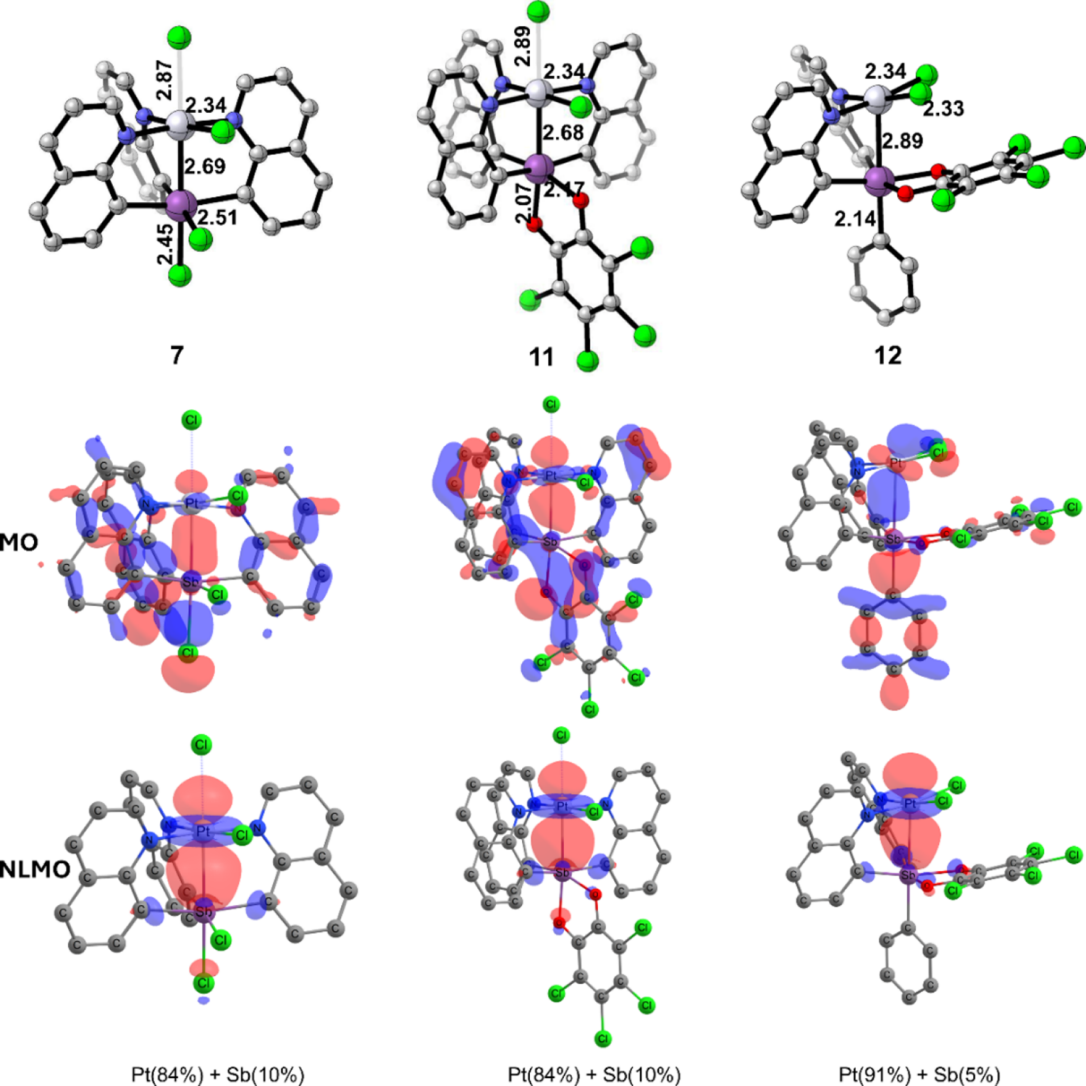
Structural features and relevant molecular orbitals (MOs) and natural localized molecular orbitals (NLMOs) of the Pt–Sb interaction in complexes **7**, **11**, and **12**.

Figure 8 provides an overview of the MOs and NLMOs for Pt–Sb interactions in complexes **7** and **11**. A complementary intrinsic bond orbital (IBO) analysis, which gave a similar bonding description, can be found in the Supporting Information. The MO for complex **7** shows multicentered σ-bonding with some bonding between the Pt and Sb centers. This orbital extends to the chloride ligand attached to the Sb center. Because it was unclear from visual inspection the contribution from Pt and Sb, we carried out NLMO calculations. This revealed that the Pt–Sb interaction is mostly a donor–acceptor type interaction for which Pt acts as an L-type ligand donating a pair of electrons to the vacant orbital on Sb. For complex **7**, the Pt–Sb interaction consists of an 84% electronic contribution from Pt and 10% contribution from Sb. A nearly identical bonding description was found for complex **11**. For complex **12**, the Pt contribution increases to 91% and Sb contribution decreases to 5%, which is likely due to the absence of the axial chloride ligand that polarizes the Pt-lone pair electrons more toward the Sb in complexes **7** and **11**. In the absence of such an axial push in complex **12**, electron density is more localized on the Pt.

Based on the above orbital analysis, a simplified bonding model can be developed for complexes **7**, **11**, and **12**. If we consider the axial chloride ligand in complexes **7** and **11** as a counterion chloride and only held together with Pt through an electrostatic, nonorbital interaction, then its complementary positive charge on Pt makes the Pt fragment a stable square planar 16-electron complex and the Sb fragment a five-coordinate structure with a vacant sixth coordination site. The 16-electron Pt^II^ fragment can then donate to the vacant orbital on the Sb^V^ fragment, which makes the Pt–Sb interaction a donor–acceptor type bonding (Pt fragment as L-type and Sb fragment as Z-type). As mentioned above, in complexes **7** and **11**, electron polarization induced by the negatively charged chloride ligand makes the Pt–Sb bond stronger and shorter. Absence of such an axial push in complex **12** makes the Pt–Sb bond longer with an axial lone pair more localized on the Pt center. Importantly, the Pt–Sb interaction in these complexes is mostly due to the geometrical constraints induced by the bridging quinoline ligand and this relatively weak donor–acceptor interaction would unlikely result in a stable intermolecular bond.

The above bonding perspective is further supported by a second-order perturbative natural bond orbital (NBO) analysis of donor–acceptor interaction energy based on the overlap and energy difference between the donor and acceptor orbitals (see the Supporting Information for values). Importantly, it is well known that this type of analysis likely overestimates the absolute interaction energies but can be used for qualitative relative values. The interaction energies computed for complexes **7** and **11** were 111.2 and 71.9 kcal/mol, respectively. These interactions showed electron delocalization between the Pt lone pair and the vacant acceptor orbital on the Sb fragment. In contrast, the interaction energy in complex **12** was calculated to be only 14.7 kcal/mol, which supports the description of a much weaker bonding interaction between Pt and Sb. The difference between **7** and **11** compared to that of **12** can be traced to the difference in Pt to Sb orbital overlap. For complexes **7** and **11**, the overlap was calculated to be 0.13 and 0.12 au, while for **12** the overlap was calculated to be 0.05 au.

## Conclusions

In conclusion, we synthesized a variety of Pt–Sb complexes with the Sb center in +3 to +5 oxidation states. Depending on the Pt and Sb oxidation states, the ligand moiety can bond in bi-, tri-, or tetradentate fashions. Oxidizing the Pt^II^–Sb^III^ complexes (**3** or **4**) with PhICl_2_ leads to a mixture of Pt–Sb complexes with different bonding interactions between the Pt and Sb centers (**7** and **8** or **9** and **10**) due to the transfer of chloride(s) from the Sb to the Pt center. The bidentate oxidant *o*-chloranil was found to circumvent the potential substrate(s) transfer from Sb to Pt center and thus selectively produce the Z-type Pt–Sb complexes (**11** or **12**). X-ray crystal structures and NLMO modeling of complexes **7**, **11**, and **12** suggested a Z-type Pt → Sb interaction, while the axial chloride in complexes **7** and **11** is better described as an anion.

## Experimental Section

### General Information

All reactions were performed in air under ambient conditions, unless otherwise noted. The synthesis of ligands **SbQ**_**3**_ and **SbQ**_**2**_**Ph** was performed under a dinitrogen atmosphere using Schlenk line techniques. Synthesis of complexes **5** and **6** was performed in a nitrogen-filled glovebox.

All NMR reactions were performed using Wilmad medium wall precision low pressure/vacuum (LPV) NMR tubes. Tetrahydrofuran (THF) and diethyl ether (Et_2_O) were dried via a potassium-benzophenone/ketyl still under a dinitrogen atmosphere and stored over activated 4 Å molecular sieves inside a glovebox. Pentane and methylene chloride were dried using a solvent purification system with activated alumina and stored under activated 3 Å molecular sieves inside a dinitrogen-filled glovebox. Chloroform-*d* and methylene chloride-*d*_2_ were stored over activated 4 Å molecular sieves inside a glovebox. SbPhCl_2_ and iodobenzene dichloride were synthesized as previously reported.^72,73^ All other chemicals were purchased from commercial sources and used as received.

NMR spectra were recorded on a Varian VNMRS 600 MHz or a Bruker Avance III 800 or 400 MHz spectrometer. All reported chemical shifts were referenced to residual ^1^H resonances (^1^H NMR) or ^13^C ^1^H resonances (^13^C{^1^H} NMR). ^1^H NMR: chloroform-*d* 7.26 ppm; methylene chloride*-d*_2_ 5.32 ppm; dimethyl sulfoxide-*d*_6_ 2.50 ppm. ^13^C{^1^H} NMR: chloroform-*d* 77.16 ppm; methylene chloride*-d*_2_ 53.84 ppm; dimethyl sulfoxide-*d*_6_ 39.52 ppm.^74^ Elemental analyses were performed at the University of Virginia, Chemistry Department, Elemental Analysis Facility, using a PerkinElmer CHNS-O series II analyzer.

### Synthesis and Characterization of Complexes

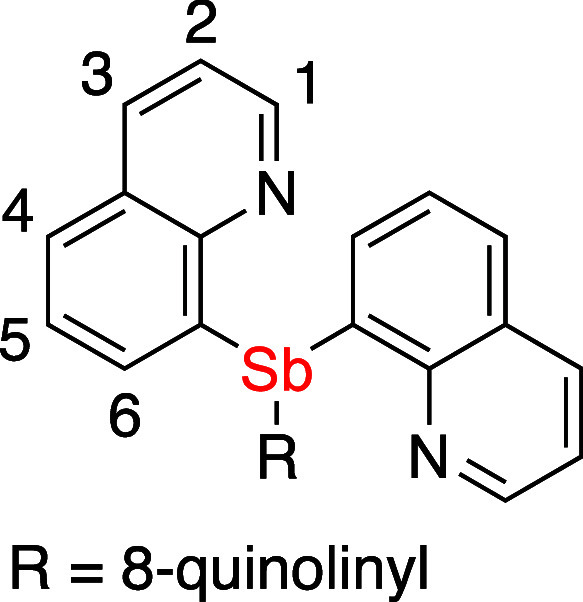



#### Tri(quinolin-8-yl)-λ^3^-stibane (SbQ_3_, **1**)

To a solution of 8-bromoquinoline (3.81 g, 18.24 mmol) in 45 mL of THF under a dinitrogen atmosphere, a 2.5 M solution of *n*-BuLi in hexanes (7.5 mL, 18.24 mmol) was syringed slowly at −78 °C. The mixture was stirred for approximately 3 h, and a solution of SbCl_3_ (1.38 g, 6.00 mmol) in 15 mL of THF was cannulated into the mixture at −78 °C. The mixture was then allowed to warm to room temperature and stirred overnight. The mixture was then concentrated using a rotavap until 5–10 mL of the solvent remained, and then EtOAc was added while stirring until a suspension of light-yellow solid in an orange solution was observed. A vacuum filtrate was used to collect the solid which is a mixture of LiCl with Q_3_Sb. The solid was redissolved using CHCl_3_ and washed with water quickly to remove the LiCl. The organic layer was concentrated to dryness to yield a light-yellow solid product (1.7 g, 57% yield) and X-ray quality crystals of SbQ_3_ were obtained by vapor diffusion of Et_2_O into a toluene solution of product. ^1^H NMR (600 MHz, CDCl_3_) δ 8.85 (dd, ^3^*J*_HH_ = 4 Hz, ^4^*J*_HH_ = 2 Hz, 3H, 1-position), 8.17 (dd, ^3^*J*_HH_ = 8 Hz, ^4^*J*_HH_ = 2 Hz, 3H, 3-position), 7.77 (d, ^3^*J*_HH_ = 8 Hz, 3H, 4- or 6-position), 7.37 (dd, ^3^*J*_HH_ = 8 Hz, ^4^*J*_HH_ = 4 Hz, 3H, 2-position), 7.22 (t, ^3^*J*_HH_ = 7 Hz, 3H, 5-position), 7.09 (d, ^3^*J*_HH_ = 7 Hz, 3H, 4- or 6-position). ^13^C{^1^H} NMR (201 MHz, CDCl_3_) δ: 152.5, 149.5, 145.0, 138.0, 136.2, 128.1, 127.9, 127.3, 121.0. Anal. Calcd for C27H18N3Sb: C, 64.06; H, 3.58; N, 8.30. Found: C, 63.92; H, 3.64; N, 8.25.
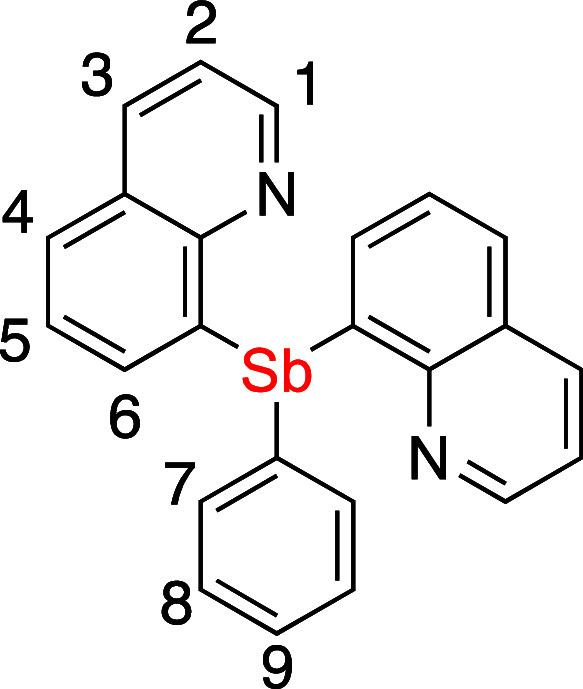


#### Di(quinolin-8-yl)-phenyl-λ^3^-stibane (SbQ_2_Ph, **2**)

A mixture of Ph_3_Sb (0.43 g, 1.22 mmol) and SbCl_3_ (0.56 g, 2.45 mmol) was stirred under dinitrogen without solvent for approximately 1 day; at the end, the solid mixture had changed to liquid (PhSbCl_2_). Then, 10 mL of distilled Et_2_O was added. To a solution of 8-bromoquinoline (1.5 g, 7.32 mmol) in 20 mL of distilled Et_2_O under a dinitrogen atmosphere, a 2.5 M solution of *n*-BuLi in hexanes (2.9 mL, 7.32 mmol) was syringed slowly at −78 °C. The mixture was allowed to warm to room temperature and stirred for about 1 h. The solution of 8-lithio-quinoline was both cooled to −78 °C and cannulated slowly into the solution of PhSbCl_2_, during which the formation of a solid precipitate was observed. The mixture was allowed to warm to room temperature and stirred overnight. The reaction mixture was concentrated using a rotavap until about 10 mL of solvent remained. The solid mixture was collected by vacuum filtration and is a mixture of LiCl with Q_2_SbPh. The solid was redissolved using DCM and washed with water to remove the LiCl. The organic layer was concentrated to dryness to yield a light-yellow solid product, which can be further purified via reprecipitation from DCM and pentanes (0.75 g, 45% yield in the presence of small amounts of **1**). **The solid can be used directly for the synthesis of Pt complexes without further purification. Small amounts of **1** as an impurity could be purified by column chromatography using EtOAc/hexanes with triethylamine. However, some of the product is lost using this method (approximately 40–50% recovery from the column). X-ray quality crystals of SbQ_2_Ph were obtained by vapor diffusion of diethyl ether into a DCM solution of product. ^1^H NMR (800 MHz, CD_2_Cl_2_) δ 8.83 (dd, ^3^*J*_HH_ = 4 Hz, ^4^*J*_HH_ = 1 Hz, 2H, 1-position), 8.22 (dd, ^3^*J*_HH_ = 8 Hz, ^4^*J*_HH_ = 2 Hz, 2H, 3-position), 7.84 (dd, ^3^*J*_HH_ = 8 Hz, ^4^*J*_HH_ = 1 Hz, 2H, 4- or 6-position), 7.42 (dd, ^3^*J*_HH_ = 8 Hz, ^4^*J*_HH_ = 4 Hz, 2H), 7.39–7.35 (m, 2H, 7- or 8-position), 7.35 (dd, ^3^*J*_HH_ = 8, 7 Hz, 2H, 5-position), 7.31–7.27 (m, 1H, 9-position), 7.27–7.22 (m, 4H, 4- or 6-position and 7- or 8-position). ^13^C{^1^H} NMR (201 MHz, CD_2_Cl_2_) δ: 152.2, 149.8, 144.3, 140.3, 137.8, 137.2, 136.7, 128.9, 128.7, 128.5, 128.4, 127.6, 121.6. Anal. Calcd for C24H17N2Sb: C, 63.33; H, 3.76; N, 6.15. Found: C, 62.95; H, 3.67; N, 5.95.
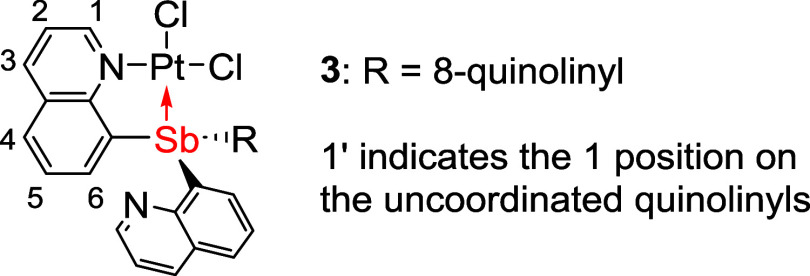


#### (SbQ_3_)PtCl_2_ (**3**)

In a screw-top pressure tube, a suspension of complex **1** (125 mg, 0.247 mmol) and PtCl_2_ (66 mg, 0.246 mmol) was stirred in approximately 3 mL of MeCN at 85 °C under air. During the heating, the suspension dissolved into a yellow solution, which slowly evolved into a yellow precipitate. After 1 h, the mixture was cooled to room temperature and then filtered through a fine porous frit. After washed with diethyl ether, the collected yellow powder on the frit was then redissolved in CHCl_3_ and filtered through a medium porous frit packed with Celite. The filtrate was then evaporated, leaving a yellow powder which was then dried under a vacuum (143 mg, 75% yield). X-ray quality crystals of SbQ_3_PtCl_2_ were obtained by vapor diffusion of diethyl ether into a CHCl_3_ solution of product. ^1^H NMR (600 MHz, CDCl_3_): δ 11.23 (d, ^3^*J*_HH_ = 5 Hz, 1H, 1-position), 9.05 (dd, ^3^*J*_HH_ = 7 Hz, ^4^*J*_HH_ = 1 Hz, 1H, 4- or 6-position), 8.60 (dd, ^3^*J*_HH_ = 4 Hz, ^4^*J*_HH_ = 2 Hz, 2H, 1′-position), 8.47 (d, ^3^*J*_HH_ = 8 Hz, 1H, 3-position), 8.40 (dd, ^3^*J*_HH_ = 7 Hz, ^4^*J*_HH_ = 1 Hz, 2H, 4′- or 6′-position), 8.18 (dd, ^3^*J*_HH_ = 8 Hz, ^4^*J*_HH_ = 2 Hz, 2H, 3′-position), 7.98 (d, ^3^*J*_HH_ = 7 Hz, 2H, 4′- or 6′-position), 7.93 (d, ^3^*J*_HH_ = 8 Hz, 1H, 4- or 6-position), 7.59 (m, 3H, 5- and 5′-position), 7.51 (dd, ^3^*J*_HH_ = 8, 6 Hz, 1H, 2-position), 7.37 (dd, ^3^*J*_HH_ = 8, 4 Hz, 2H, 2′-position). ^13^C{^1^H} NMR (201 MHz, CDCl_3_) δ: 158.0, 157.9, 151.8, 150.2, 149.4, 140.7, 139.8, 138.9, 136.4, 134.7, 132.4, 131.4, 131.0, 130.7, 128.6, 128.1, 127.5, 123.1, 122.1. **The ^13^C resonances at 158.0 and 157.9 ppm show as a doublet due to the insufficient default ^1^H decoupling bandwidths which did not cover the most downfield-shifted proton resonances (i.e., over 11 ppm, Supporting Information, Section 5). Anal. Calcd for C27H18Cl2N3PtSb: C, 42.00; H, 2.35; N, 5.44. Found: C, 41.33 H; 2.45; N, 5.29.
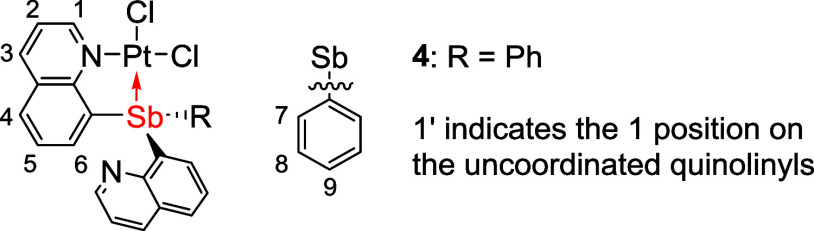


#### (SbQ_2_Ph)PtCl_2_ (**4**)

In a screw-top pressure tube, a suspension of complex **2** (62 mg, 0.136 mmol) and PtCl_2_ (36 mg, 0.136 mmol) in approximately 4 mL of MeCN was stirred under ambient conditions at 85 °C for ∼1 h. During the heating, the suspension turned into a yellow solution and then slowly evolved into a yellow precipitate. After the mixture was cooled back to room temperature, DCM was added to dissolve the solid precipitate. After evaporating the solvents, the crude product can be isolated as a yellow solid, which can be further purified via reprecipitation using pentane and DCM or CHCl_3_ (83 mg, 85% yield). X-ray quality crystals of SbQ_2_PhPtCl_2_ can be obtained by vapor diffusion of diethyl ether into a CHCl_3_ or 1,2-dichloroethane solution of the product. ^1^H NMR (600 MHz, CDCl_3_) δ 11.20 (dd, ^3^*J*_HH_ = 6 Hz, ^4^*J*_HH_ = 2 Hz, 1H, 1-position), 9.13 (dd, ^3^*J*_HH_ = 4 Hz, ^4^*J*_HH_ = 2 Hz, 1H, 4- or 6-position), 9.11 (dd, ^3^*J*_HH_ = 7 Hz, ^4^*J*_HH_ = 2 Hz, 1H, 1′-position), 8.85 (dd, ^3^*J*_HH_ = 7 Hz, ^4^*J*_HH_ = 1 Hz, 1H), 8.45 (dd, ^3^*J*_HH_ = 8 Hz, ^4^*J*_HH_ = 2 Hz, 1H, 3-position), 8.28 (dd, ^3^*J*_HH_ = 8 Hz, ^4^*J*_HH_ = 2 Hz, 1H, 4- or 6-position), 8.14–8.09 (m, 2H), 8.03 (dd, ^3^*J*_HH_ = 8 Hz, ^4^*J*_HH_ = 1 Hz, 1H), 7.99 (dd, ^3^*J*_HH_ = 8 Hz, ^4^*J*_HH_ = 2 Hz, 1H), 7.71 (td, ^3^*J*_HH_ = 8 Hz, ^4^*J*_HH_ = 4 Hz, 2H), 7.58 (dd, ^3^*J*_HH_ = 8, 4 Hz, 1H, 5-position), 7.49 (dd, ^3^*J*_HH_ = 8, 6 Hz, 1H, 2-position), 7.45–7.36 (m, 3H). ^13^C{^1^H} NMR (201 MHz, CDCl_3_) δ: 158.1, 152.4, 150.8, 149.2, 141.0, 140.7, 140.0, 137.1, 135.6, 132.3, 132.1, 131.7, 131.4, 130.9, 130.8, 129.4, 128.9, 128.5, 128.0, 127.7, 123.5, 122.6. Anal. Calcd for C24H17Cl2N2PtSb: C, 39.97; H, 2.38; N, 3.88. Found: C, 40.41; H, 2.48; N, 3.85.
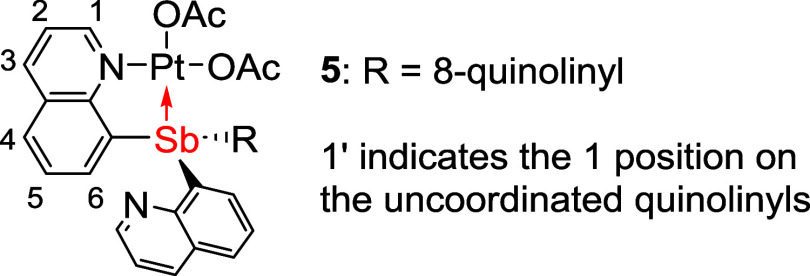


#### (SbQ_3_)Pt(OAc)_2_ (**5**)

In a 100 mL flask, a mixture of complex **3** (152 mg, 0.196 mmol) and silver acetate (70 mg, 0.422 mmol) in 25 mL of DCM was stirred under N_2_ at room temperature. After being stirred overnight, the reaction mixture was filtered through Celite, leaving a yellow filtrate. The filtrate was then evaporated, leaving a yellow residue, which was redissolved in a minimal amount of DCM. Then, pentane was used to precipitate out the product from the DCM solution as an off-white powder (41 mg, 25% yield). Efforts to further precipitate solid gave a yellowish powder which was impure based on the ^1^H NMR spectroscopy. X-ray quality crystals of SbQ_3_Pt(OAc)_2_ were obtained by vapor diffusion of diethyl ether into a DCM solution of product. ^1^H NMR (600 MHz, CD_2_Cl_2_) δ 10.03 (dd, ^3^*J*_HH_ = 6 Hz, ^4^*J*_HH_ = 2 Hz, 1H, 1-position), 8.63 (dd, ^3^*J*_HH_ = 7 Hz, ^4^*J*_HH_ = 2 Hz, 1H, 4- or 6-position), 8.55 (dd, ^3^*J*_HH_ = 4 Hz, ^4^*J*_HH_ = 2 Hz, 2H, 1′-position), 8.40 (dd, ^3^*J*_HH_ = 8 Hz, ^4^*J*_HH_ = 2 Hz, 1H, 3-position), 8.31 (dd, ^3^*J*_HH_ = 7 Hz, ^4^*J*_HH_ = 1 Hz, 2H, 4′- or 6′-position), 8.23 (dd, ^3^*J*_HH_ = 8 Hz, ^4^*J*_HH_ = 2 Hz, 2H, 3′-position), 8.00 (dd, ^3^*J*_HH_ = 8 Hz, ^4^*J*_HH_ = 1 Hz, 2H, 4′- or 6′-position), 7.70 (dd, ^3^*J*_HH_ = 8 Hz, ^4^*J*_HH_ = 2 Hz, 1H, 4- or 6-position), 7.63 (dd, ^3^*J*_HH_ = 8, 7 Hz, 2H, 5′-position), 7.47 (dd, ^3^*J*_HH_ = 8, 6 Hz, 1H, 2-position), 7.38 (dd, ^3^*J*_HH_ = 8, 4 Hz, 2H, 2′-position), 7.28 (d, ^3^*J*_HH_ = 8, 7 Hz, 1H, 5-position), 2.26 (s, 3H, acetate–C*H*_3_),1.96 (s, 3H, acetate–C*H*_3_). ^13^C{^1^H} NMR (201 MHz, CD_2_Cl_2_) δ: 179.2, 177.2, 155.2, 151.3, 150.2, 149.5, 144.6, 140.0, 139.7, 139.5, 136.7, 136.0, 130.6, 130.1, 130.0, 128.8, 128.1, 127.3, 122.2, 122.0, 25.7, 19.1. Anal. Calcd for C31H24N3O4PtSb: C, 45.44; H, 2.95; N, 5.13. Found: C, 45.15; H, 3.02; N, 4.97.
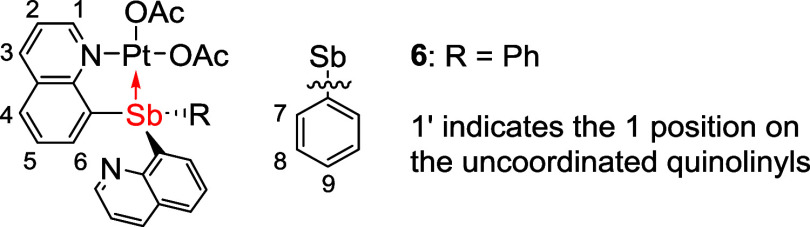


#### (SbQ_2_Ph)Pt(OAc)_2_ (**6**)

In a 100 mL flask, a mixture of complex **4** (230 mg, 0.319 mmol) and silver acetate (46 mg, 0.673 mmol) in 25 mL of DCM was stirred under N_2_ at room temperature. The next day, the reaction mixture was filtered through Celite, and the yellow filtrate was then evaporated to afford a yellow residue as a crude product. The yellow residue was redissolved in a minimal amount of DCM, and then, pentane was added to precipitate out the solid product. The product was collected via vacuum filtration as a yellow-white powder (80 mg, 33% yield). X-ray quality crystals of SbQ_2_PhPt(OAc)_2_ were obtained by vapor diffusion using DCM and diethyl ether. ^1^H NMR (600 MHz, CDCl_3_) δ 10.04 (dd, ^3^*J*_HH_ = 6 Hz, ^4^*J*_HH_ = 2 Hz, 1H, 1-position), 8.82 (dd, ^3^*J*_HH_ = 4 Hz, ^4^*J*_HH_ = 2 Hz, 1H, 4- or 6-position), 8.49 (dd, ^3^*J*_HH_ = 7 Hz, ^4^*J*_HH_ = 1 Hz, 1H, 1′-position), 8.44 (dd, ^3^*J*_HH_ = 7 Hz, ^4^*J*_HH_ = 1 Hz, 1H), 8.35 (dd, ^3^*J*_HH_ = 8 Hz, ^4^*J*_HH_ = 2 Hz, 1H), 8.17 (dd, ^3^*J*_HH_ = 8 Hz, ^4^*J*_HH_ = 2 Hz, 1H), 7.94 (ddd, ^3^*J*_HH_ = 7, 6 Hz, ^4^*J*_HH_ = 1 Hz, 3H), 7.74 (dd, ^3^*J*_HH_ = 8 Hz, ^4^*J*_HH_ = 1 Hz, 1H), 7.63 (t, ^3^*J*_HH_ = 8 Hz, 1H), 7.46–7.40 (m, 4H), 7.40–7.36 (m, 2H), 2.37 (s, 3H, acetate–C*H*_3_), 2.10 (s, 3H, acetate–C*H*_3_). ^13^C{^1^H} NMR (201 MHz, CDCl_3_) δ: 179.6, 178.1, 155.4, 152.0, 150.2, 149.2, 143.6, 139.6, 139.5, 138.9, 136.5, 135.7, 135.5, 132.4, 130.7, 130.3, 130.2, 129.8, 128.8, 128.7, 128.2, 127.3, 122.3, 121.9, 25.7, 19.2. Anal. Calcd for C28H23N2O4PtSb: C, 43.77; H, 3.02; N, 3.65. Found: C, 43.51; H, 2.97; N, 3.64.
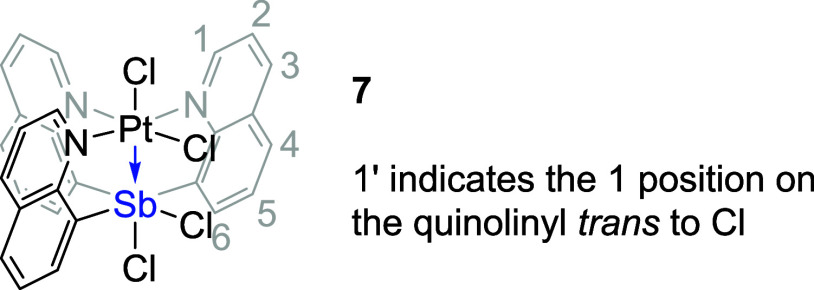


#### (Cl_2_SbQ_3_)PtCl_2_ (**7**)

In a 100 mL flask, a suspension of complex **3** (93 mg, 0.121 mmol) and iodobenzene dichloride (34 mg, 0.122 mmol) in DCM was stirred under N_2_ overnight. By the next day, a white precipitate had formed. The mixture was then transferred to a 4-dram scintillation vial, and the supernatant was removed via a pipette. The remaining white solid was washed with a minimal amount of DCM and then dried to yield the product (47 mg, 46% yield). X-ray quality crystals of Cl_2_SbQ_3_PtCl_2_ were obtained by vapor diffusion of diethyl ether into a DCM solution of product. ^1^H NMR (600 MHz, CDCl_3_) δ 11.54 (d, ^3^*J*_HH_ = 5 Hz, 1H, 1′-position), 11.29 (d, ^3^*J*_HH_ = 5 Hz, 2H, 1-position), 9.20 (d, ^3^*J*_HH_ = 7 Hz, 2H), 9.01 (d, ^3^*J*_HH_ = 7 Hz, 1H), 8.43 (d, ^3^*J*_HH_ = 8 Hz, 2H), 8.37 (d, ^3^*J*_HH_ = 7 Hz, 1H), 7.90 (d, ^3^*J*_HH_ = 8 Hz, 2H), 7.76 (t, ^3^*J*_HH_ = 8 Hz, 3H), 7.62 (t, ^3^*J*_HH_ = 7 Hz, 2H), 7.59–7.54 (m, 1H), 7.54–7.50 (m, 1H). The ^13^C NMR spectrum cannot be obtained with a reasonable signal-to-noise ratio due to the poor solubility of complex **7**. Anal. Calcd for C27H18Cl4N3PtSb: C, 38.46; H, 2.15; N, 4.98. Found: C, 37.97; H, 2.21; N, 4.70.
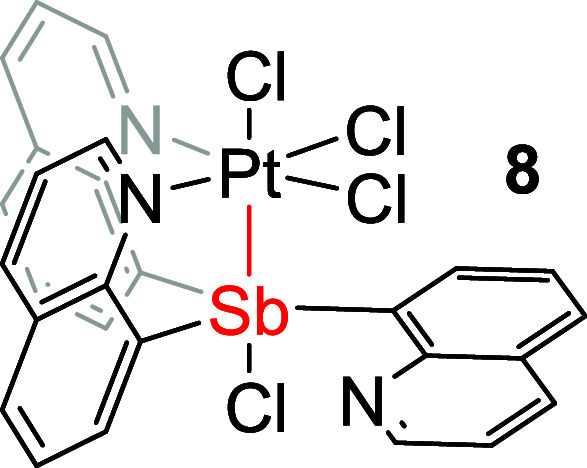


#### (ClSbQ_3_)PtCl_3_ (**8**)

Crystals suitable for SC-XRD were found from reactions to make **7** and could not be isolated analytically pure. Vapor diffusion using pentanes and DCM in freezer (see Supporting Information, Section 2).
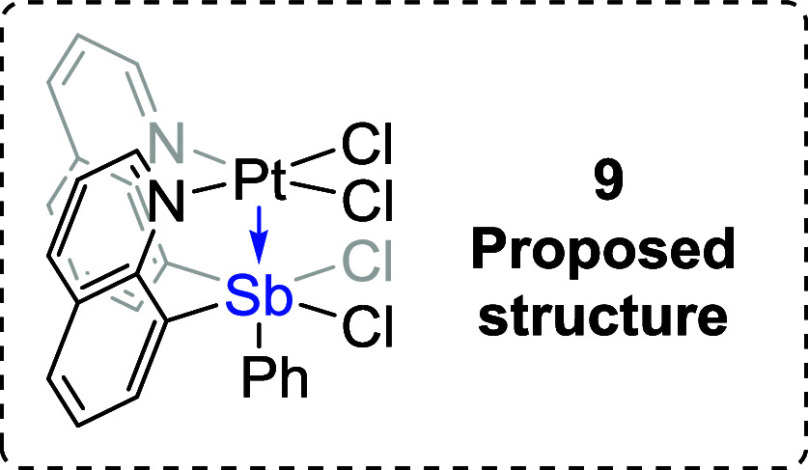


#### Proposed (Cl_2_SbQ_2_Ph)PtCl_2_ (**9**)

Formed in the synthesis of complex **10** (see below). The leftover DCM filtrate was concentrated until about 1 mL was left, and pentane was added to precipitate out complex **9** (22 mg, with ∼7% complex **10**). ^1^H NMR (600 MHz, CDCl_3_) δ 11.00 (brs, 2H, 1-position), 9.55 (brs, 2H), 8.59 (dd, ^3^*J*_HH_ = 8 Hz, ^3^*J*_HH_ = 1 Hz, 2H), 8.18 (d, ^3^*J*_HH_ = 8 Hz, 2H), 7.90 (t, ^3^*J*_HH_ = 8 Hz, 2H), 7.77 (dd, ^3^*J*_HH_ = 8, 6 Hz, 2H), 7.30 (t, ^3^*J*_HH_ = 8 Hz, 1H), 7.16 (t, ^3^*J*_HH_ = 8 Hz, 2H), 6.96 (brs, 2H). ^1^H NMR (600 MHz, CD_2_Cl_2_) δ 11.20 (d, ^3^*J*_HH_ = 5 Hz, 2H), 9.52 (d, ^3^*J*_HH_ = 7 Hz, 2H), 8.70 (d, ^3^*J*_HH_ = 8 Hz, 2H), 8.30 (d, ^3^*J*_HH_ = 8 Hz, 2H), 8.01–7.94 (m, 4H), 7.88 (t, ^3^*J*_HH_ = 7 Hz, 2H), 7.60 (d, ^3^*J*_HH_ = 7 Hz, 1H), 7.52 (t, ^3^*J*_HH_ = 8 Hz, 2H).
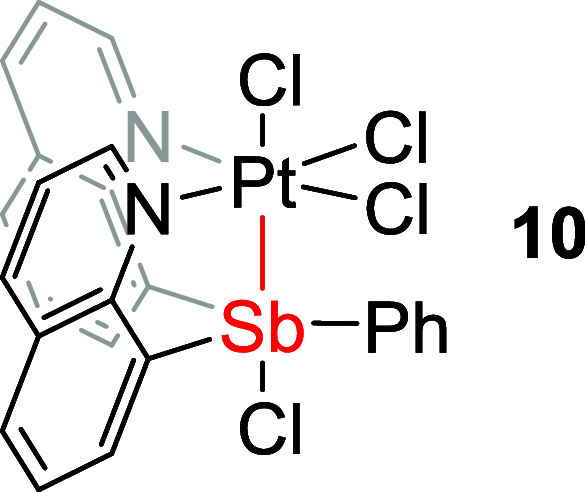


#### (ClSbQ_2_Ph)PtCl_3_ (**10**)

Inside a dinitrogen-filled glovebox, a suspension of complex **4** (50 mg, 0.069 mmol) and PhICl_2_ (20 mg, 0.073 mmol) in 15 mL of DCM was stirred at room temperature. In approximately 10 min, complex **10** started to precipitate as an off-white solid. After continuous stirring for approximately 30 min, no additional solid formed. Then, the crude complex **10** (29 mg) was collected by vacuum filtration as an off-white solid. The crude product can be further purified by washing with a minimal amount of DCM, and the pure product was collected after drying as a white powder (20 mg, 36% yield). X-ray quality crystals of ClSbQ_2_PhPtCl_3_ can be obtained by slow evaporation from a CD_2_Cl_2_ solution of product. ^1^H NMR (600 MHz, DMSO-*d*_6_) δ 10.68 (brs, 2H, 1-position), 9.30 (d, ^3^*J*_HH_ = 8 Hz, 2H), 9.01 (d, ^3^*J*_HH_ = 8 Hz, 2H), 8.48 (d, ^3^*J*_HH_ = 8 Hz, 2H), 8.06 (q, ^3^*J*_HH_ = 8 Hz, 4H), 7.93 (d, ^3^*J*_HH_ = 8 Hz, 2H), 7.59 (d, ^3^*J*_HH_ = 8 Hz, 1H), 7.53 (t, ^3^*J*_HH_ = 8 Hz, 2H). ^13^C{^1^H} NMR (201 MHz, DMSO-*d*_6_). δ 155.2, 146.6, 143.2, 141.2, 138.1, 136.4, 133.7, 132.2, 131.5, 130.9, 129.0, 128.4, 123.9. Anal. Calcd for C24H17Cl4N2PtSb: C, 36.39; H, 2.16; N, 3.54. Found: C, 35.29; H, 2.20; N, 3.37.
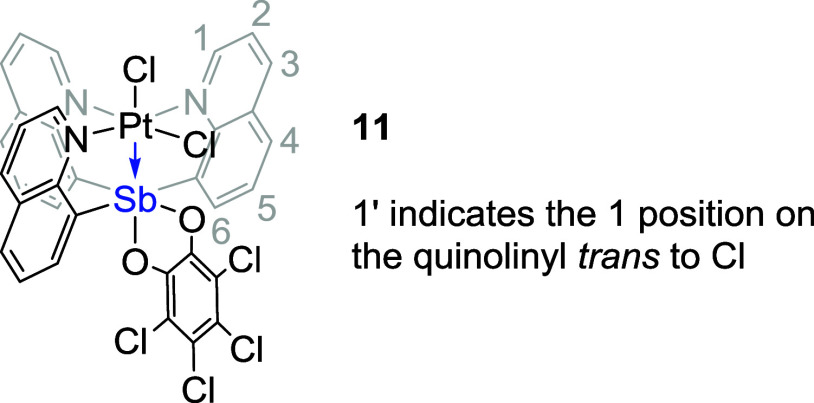


#### {(*o*-Chloranil)SbQ_3_}PtCl_2_ (**11**)

In a 100 mL round-bottom flask, a mixture of complex **3** (29 mg, 0.038 mmol) and 3,4,5,6-tetrachloro-1,2-benzoquinone (10 mg, 0.042 mmol) was stirred in 20 mL of DCM for 2 days. The reaction began as a suspension and then slowly turned orange over 2 days. After evaporation of the solvent via a vacuum, the orange/tan residue was washed with diethyl ether until no more color could be washed out. The solid was then collected and dried in vacuo to yield the product as an orange powder (18 mg, 48% yield). X-ray quality crystals were obtained by vapor diffusion of diethyl ether into a chloroform solution of product. ^1^H NMR (600 MHz, CD_2_Cl_2_) δ 12.13 (d, ^3^*J*_HH_ = 6 Hz, 2H, 1-position), 11.98 (d, ^3^*J*_HH_ = 6 Hz, 1H, 1′-position), 9.23 (dd, ^3^*J*_HH_ = 7 Hz, ^4^*J*_HH_ = 1 Hz, 2H), 8.98 (d, ^3^*J*_HH_ = 7 Hz, 1H), 8.35 (d, ^3^*J*_HH_ = 8 Hz, 2H), 8.25 (d, ^3^*J*_HH_ = 8 Hz, 1H), 7.80 (d, ^3^*J*_HH_ = 8 Hz, 2H, 4- or 6-position), 7.71–7.64 (m, 3H, 3-, 5- and 4′- or 6′-position), 7.60 (t, ^3^*J*_HH_ = 7 Hz, 3H, 2-position), 7.53 (t, ^3^*J*_HH_ = 8 Hz, 1H, 5′-position). ^13^C{^1^H} NMR (201 MHz, CD_2_Cl_2_) δ: 160.5, 160.4, 158.5, 158.4, 152.9, 151.6, 149.6, 148.9, 146.8, 143.7, 142.3, 142.1, 137.5, 136.4, 130.7, 130.6, 130.2, 130.1, 128.7, 128.2, 126.0, 125.5, 123.1, 122.6, 121.7, 121.0. **The ^13^C resonances at 160.5 and 160.4 ppm, as well as 158.5 and 158.4 ppm, show as doublets due to the insufficient default ^1^H decoupling bandwidths which did not cover the most downfield-shifted proton resonances (i.e., over 11 ppm, Supporting Information, Section 5). Anal. Calcd for C33H18Cl6N3O2PtSb: C, 38.93; H, 1.78; N, 4.13. Found: C, 38.69; H, 1.80; N, 3.92.
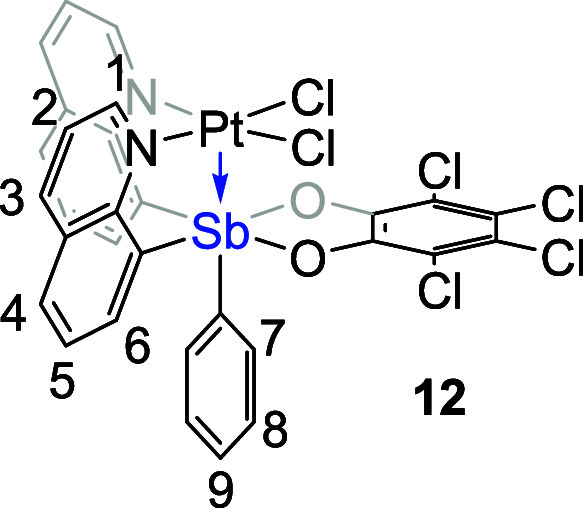


#### {(*o*-Chloranil)SbQ_2_Ph}PtCl_2_ (**12**)

In a 100 mL round-bottom flask, a mixture of complex **4** (59 mg, 0.079 mmol) and 3,4,5,6-tetrachloro-1,2-benzoquinone (23 mg, 0.090 mmol) was stirred in 20 mL of DCM for 2 days under ambient atmosphere. The color of the reaction mixture changed from red to orange. After 2 days, the solvent was evaporated to yield a crude product, which was then washed with diethyl ether to yield the pure product as a red/orange powder (37 mg, 48% yield). X-ray quality crystals were obtained by vapor diffusion of diethyl ether into an acetonitrile/DCM solution of product. ^1^H NMR (600 MHz, DMSO-*d*_6_) δ 10.21 (dd, ^3^*J*_HH_ = 6 Hz, ^4^*J*_HH_ = 2 Hz, 2H, 1-position), 8.92 (dd, ^3^*J*_HH_ = 8 Hz, ^4^*J*_HH_ = 1 Hz, 2H, 3-position), 8.28 (d, ^3^*J*_HH_ = 8 Hz, 2H, 4- or 6-position), 8.14 (dd, ^3^*J*_HH_ = 7 Hz, ^4^*J*_HH_ = 1 Hz, 2H, 4- or 6-position), 7.94 (dd, ^3^*J*_HH_ = 8, 6 Hz, 2H, 2-position), 7.74 (t, ^3^*J*_HH_ = 8 Hz, 2H, 5-position), 7.71–7.67 (m, 3H, 8- and 9-position), 7.62 (dd, ^3^*J*_HH_ = 7 Hz, ^4^*J*_HH_ = 2 Hz, 2H, 7-position). ^13^C{^1^H} NMR (201 MHz, DMSO-*d*_6_) δ: 155.3, 146.8, 145.3, 143.4, 142.0, 138.2, 137.0, 135.6, 132.0, 131.0, 130.9, 129.3, 128.1, 123.8, 117.7, 115.4. Anal. Calcd for C30H17Cl6N2O2PtSb: C, 37.26; H, 1.77; N, 2.90. Found: C, 37.71; H, 2.06; N, 2.89.

### Computational Methods

The M06/def2-SVP method and basis set in Gaussian 16 with the default ultrafine integration grid were used for all geometry optimizations. During both geometry optimization and single-point calculations, solvent effects were incorporated using the conductor-like polarizable continuum model (CPCM) method for dichloroethane (DCE). Single-point calculations of the optimized structures were carried out at the B2PLYP-D3(BJ)/def2-TZVPD level using the program ORCA-5.0.3 with the default integration grid. Resolution of identity approximation for both Coulomb and exchange integrals (RI-JK) with an auxiliary def2-TZVPD/C def2/JK basis set was used in ORCA calculations. NLMO calculations and second-order perturbation analyses were performed using the NBO package, as implemented in Gaussian 16.

## Supporting Information Available

Additional experimental procedures and details, NMR spectra, crystal structure data, computational details, Cartesian coordinates for the calculated structures and absolute energies (PDF)
Complexes_**7**_**8**_**9**_**10**_**11**_**12** (XYZ)

## Supplementary Material

om4c00221_si_001.pdf

om4c00221_si_002.xyz
