# Supplementary material for: Synthesis of Quinoline-Based Pt–Sb Complexes with L- or Z-Type Interaction: Ligand-Controlled Redox via Anion Transfer
Source: Organometallics. 2024 Aug 13;43(17):1789–802. doi: 10.1021/acs.organomet.4c00221 (PMC11388469; doi:10.1021/acs.organomet.4c00221)
Supplement: Supplementary file 1 — om4c00221_si_001.pdf [file om4c00221_si_001.pdf]

Supporting Information
for

Synthesis of Quinoline-Based Pt–Sb Complexes with L- or Z-Type Interaction: Ligand Controlled Redox via Anion Transfer

Christopher K. Webber[†,#], Fanji Kong[†,#], Jugal Kumawat[§], Jyothish Joy[§], Erica K. Richardson[†], Paolo Siano[†], Diane A. Dickie[†], Daniel H. Ess[§,*] and T. Brent Gunnoe[†,*]

[†]Department of Chemistry, University of Virginia; Charlottesville, Virginia 22904, United States.

[§]Department of Chemistry and Biochemistry, Brigham Young University; Provo, Utah 84604, United States.

*Corresponding authors. Email: tbg7h@virginia.edu, dhe@chem.byu.edu

[#]These authors contributed equally to this work.

Table of Contents

page

1. General Information S3
2. Reaction of Complex (SbQ_3)$PtCl_2$ (**3**) with $PhICl_2$ S4
3. Conversion of Complex (Cl_2SbQ_2Ph)$PtCl_2$ (**9**) to ($ClSbQ_2Ph$)$PtCl_3$ (**10**) S8
4. DFT Modeling of Chloride Transfer Between Pt and Sb. S11
5. Unexpected Doublets Observed in $^{13}C\{^1H\}$ NMR Spectra S12
6. Reaction of Complex {(*o*-chloranil)SbQ_3}$PtCl_2$ (**11**) with MeOH S13
7. Reaction of Complex **7** with HBF_4 .... S14
8. EXSY experiments of Complexes **5** and **6** S15
9. FT-IR Analysis of Complexes **5** and **6** S16
10. NMR Spectra S17
11. X-Ray Crystal Structure Data S31
12. Absolute Energies for the Calculated Structures S37
13. NBO Second Order Perturbation Analysis of the Pt–Sb Bond S38
14. References S39

1. General Information

All NMR reactions were performed using Wilmad medium wall precision low pressure/vacuum (LPV) NMR tubes (or referred to J-Young tubes). Tetrahydrofuran (THF) and diethyl ether (Et_2O) were dried via potassium-benzophenone/ketyl stills under a dinitrogen atmosphere and stored over activated 4Å molecular sieves inside a glovebox. Pentane and methylene chloride were dried using a solvent purification system with activated alumina and stored over activated 3Å molecular sieves inside a dinitrogen filled glovebox. Chloroform-d_1 and methylene chloride-d_2 were stored over activated 4Å molecular sieves inside a dinitrogen filled glovebox. $SbPhCl_2$ and iodobenzene dichloride were synthesized as previously reported.[1-2] All other chemicals were purchased from commercial sources and used as received.

NMR spectra were obtained on Varian VNMRS 600 MHz or a Bruker Avance III 800 MHz or 400 MHz spectrometer. All reported chemical shifts were referenced to residual ^{1}H resonances (^{1}H NMR) or ^{13}C resonances (^{13}C{^{1}H} NMR). ^{1}H NMR: chloroform-d_1 7.26 ppm; methylene chloride-d_2 5.32 ppm; dimethyl sulfoxide-d_6 2.50 ppm. ^{13}C{^{1}H} NMR: chloroform-d_1 77.16 ppm; methylene chloride-d_2 53.84 ppm; dimethyl sulfoxide-d_6 39.52 ppm.[3] Powder X-Ray diffraction data was obtained on Panalytical Empyrean Multipurpose X-Ray Diffractometer. Elemental analyses were performed by the University of Virginia Chemistry Department Elemental Analysis Facility using a Perkin-Elmer CHNS-O series II analyzer.

2. Reaction of Complex (SbQ_3)$PtCl_2$ (3) with $PhICl_2$

Upon stirring complex **3** in $CDCl_3$ with $PhICl_2$ at r.t. for 10–15 mins, an intermediate was observed and later $Cl_2SbQ_3PtCl_2$ (**7**) started to form and precipitate out from the reaction solution (**Figure S1**). To understand the identity of the intermediate, a separate identical reaction was stopped at 10 mins, and vacuumed to dryness. The resulting powder mixture was examined via powder XRD analysis (**Figure S3**), and the resulted pattern qualitatively showed the existence of ($ClSbQ_3$)$PtCl_3$ (**8**) in the reaction mixture, based on the predicted powder XRD pattern from the X-ray crystal structure of **8** (**Figure S2**).

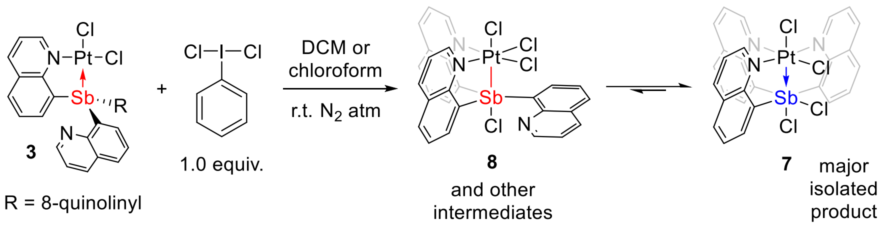

Scheme S1. Oxidation of complex **3** using $PhICl_2$.

After the powder XRD analysis, the sample was redissolved in $CDCl_3$ and monitored using ^1H NMR spectroscopy (**Figure S4**). Complex **3**, **7**, and **8** were all present in the ^1H NMR spectra as expected. After 1 hour, the integration ratio of complex **7** to **8** increased, which seems to be consistent with the conversion from **8** to **7**. However, due to the solubility issue of **7** in $CDCl_3$, the resonances corresponding to **7** may not dramatically increase. Another unknown species was also observed in the first ^1H NMR spectrum after XRD, and later decreased in intensity after 1 hour.

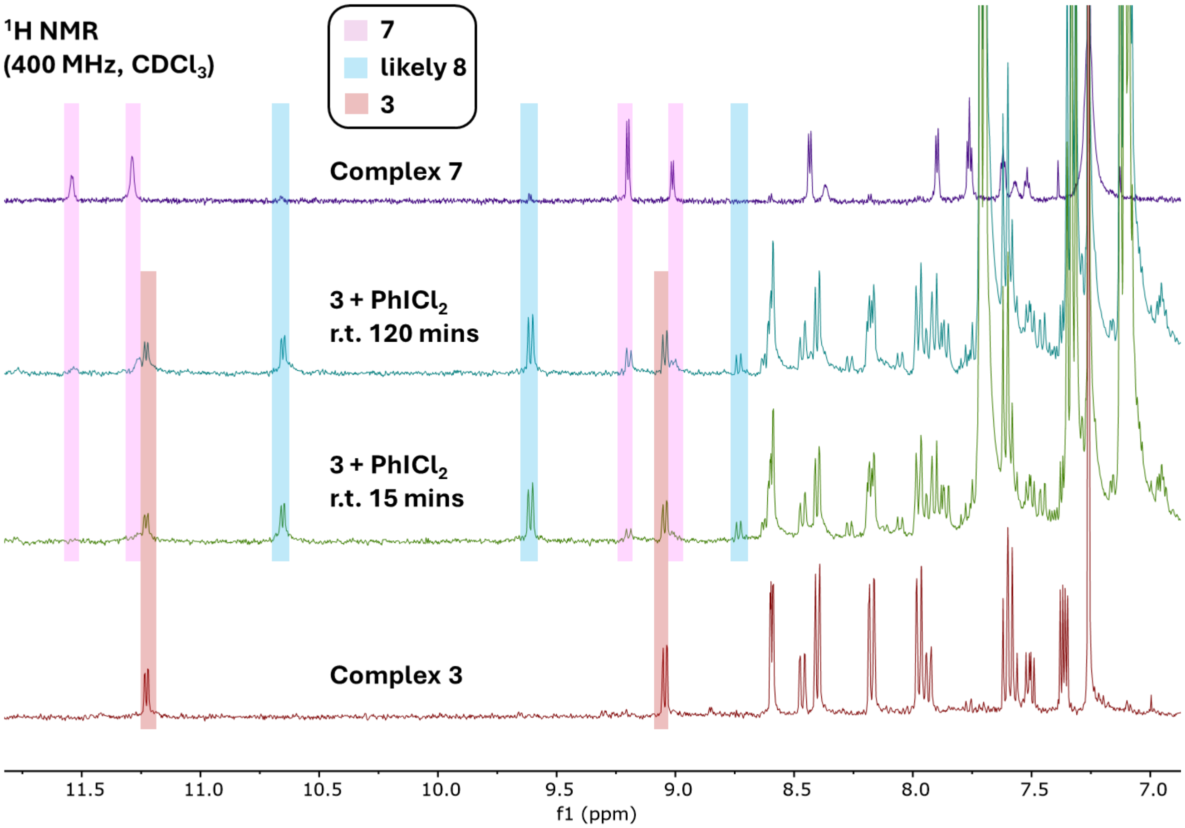

Figure S1. ^{1}H NMR spectra of the reaction of complex **3** with $PhICl_2$ in $CDCl_3$.

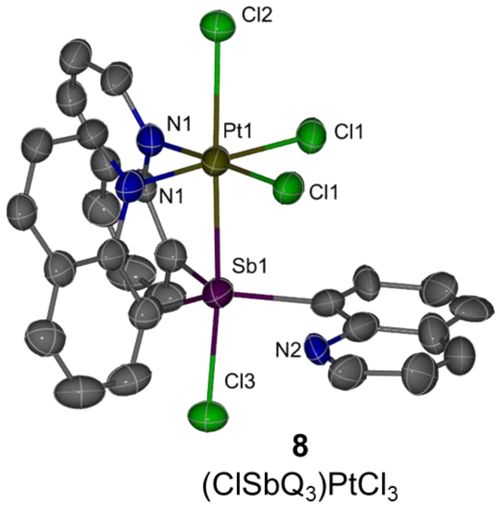

8
($ClSbQ_3$)$PtCl_3$

Figure S2. ORTEPs of ($ClSbQ_3$)$PtCl_3$ (**8**). Ellipsoids are drawn at the 50% probability level. Selected bond lengths and distances (Å): Pt1–Sb 2.6409(6), Pt1–Cl1 2.3052(15), Pt1–Cl2 2.7064(18), Pt1–N1 2.050(5), Sb1–Cl3 2.4899(19), H_a···Cl2 2.5357(16). *Note:* H_a is the calculated position of the quinoline proton ortho to the nitrogen.

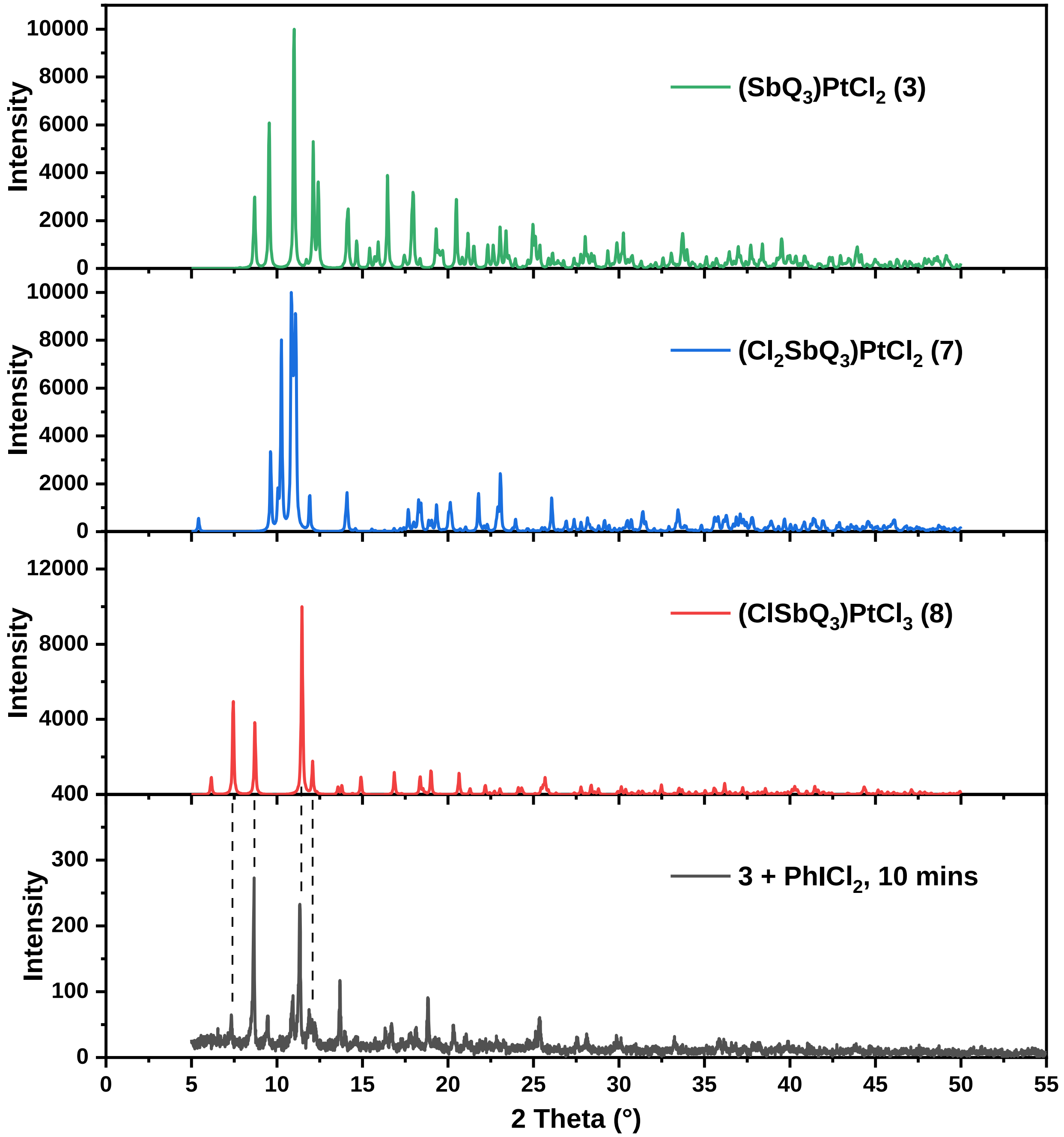

Figure S3. Powder XRD pattern of isolated solid mixture from the reaction of complex **3** with $PhICl_2$ in DCM after 10 mins and other related complexes.

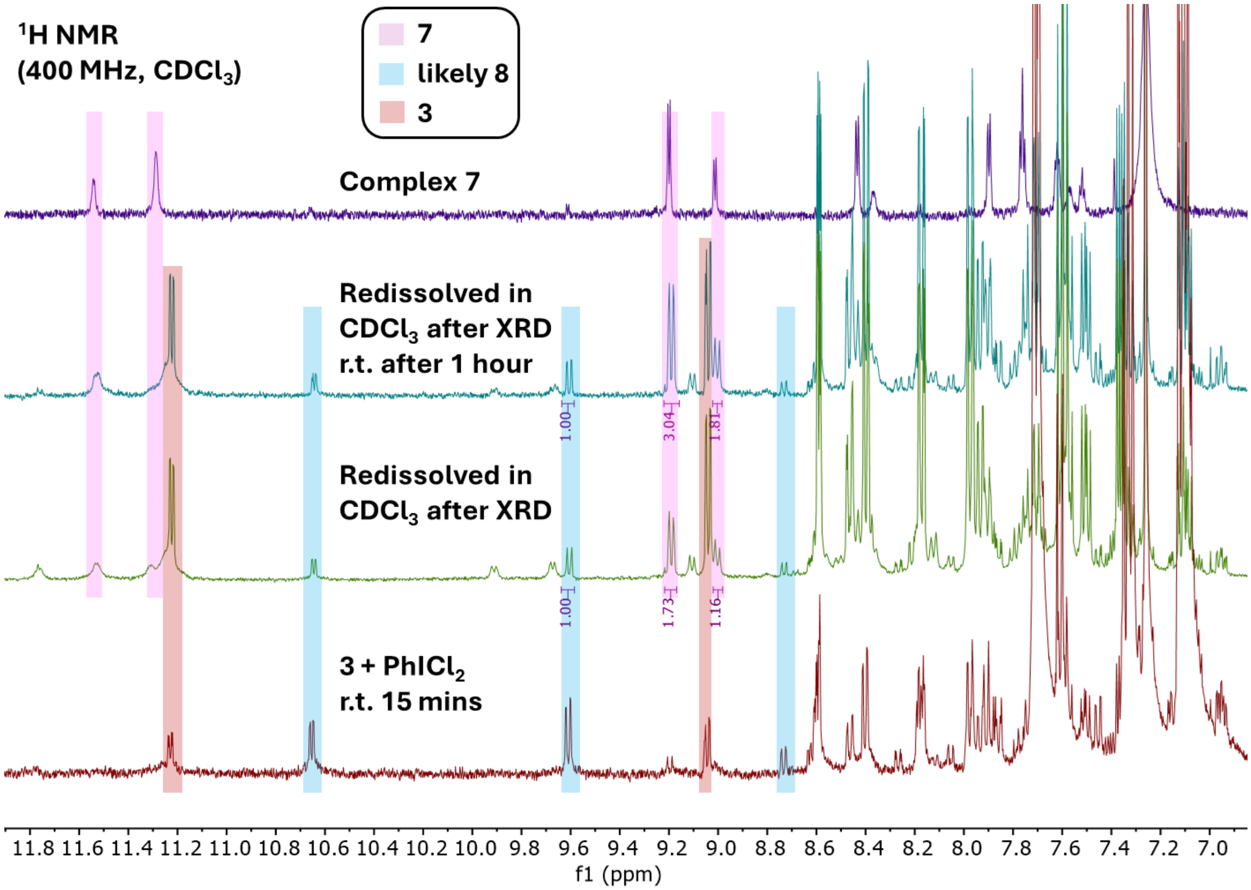

Figure S4. ^{1}H NMR spectra of redissolved sample in $CDCl_3$ after powder XRD analysis.

The Sb ligand coordinates to Pt in a tetradentate fashion in complex **7** via Sb and three quinolines while it is tridentate in complex **8** with one of the quinoline side-arms uncoordinated to Pt. The conversion from **8** to **7** is likely attributed to the chloride transfer from Pt to Sb center along with the coordination of a quinoline arm. Although we cannot definitively conclude that the first observed intermediate has the structure of **8**, the current experimental evidence supports this speculation.

3. Conversion of Complex (Cl_2SbQ_2Ph)$PtCl_2$ (9) to ($ClSbQ_2Ph$)$PtCl_3$ (10)

During the attempts to oxidize complex **4** using $PhICl_2$, two different products were observed (**Scheme S2**). The formed complex **9** is soluble in DCM, while the other product (complex **10**) is barely soluble in DCM and is present as a yellow precipitate.

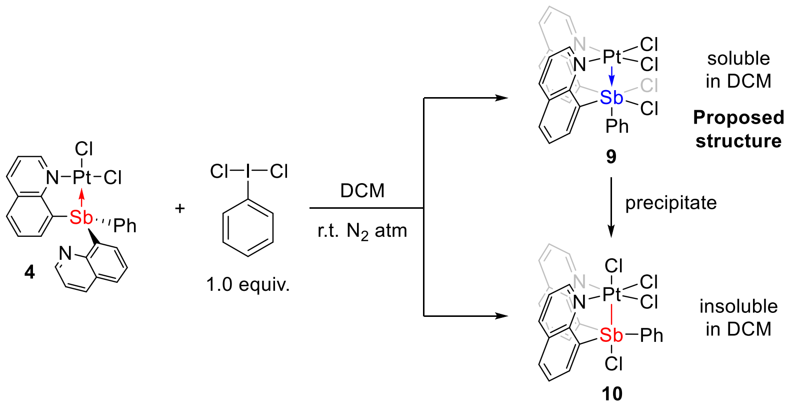

Scheme S2. Oxidation of complex **4** using $PhICl_2$ and conversion from proposed complex **9** to ($ClSbQ_2Ph$)$PtCl_3$ (**10**).

Upon storing the freshly isolated crude complex **9** in $CDCl_3$ in a J-Young tube under dinitrogen overnight, all the proton resonances belonging to complex **9** disappeared leaving only a minor unknown impurity from the reaction (**Figure S5**), and complex **10** was formed as yellow precipitate inside the J-Young tube. As shown in **Figure S6**, the observed minor impurity was present in the original crude complex **9**. Complex **10** was characterized in DMSO-d_6 due to its poor solubility in $CDCl_3$ or DCM-d_2. However, we were able to obtain a readable ^{1}H NMR spectrum in $CDCl_3$ using an 800 MHz spectrometer with extended number of scans and a line-broadening factor to set 1.0 for better signal-to-noise ratio. We found complex **10** was also present in the $CDCl_3$ solution of complex **9**, which is consistent with the conversion of **9** to **10**. In addition, the presence of a more downfield shifted H_a in complex **10** than **9** in $CDCl_3$ (**Figure S6**) is also consistent with the proposed structure of **9** without an axial chloride *trans* to the Sb center.

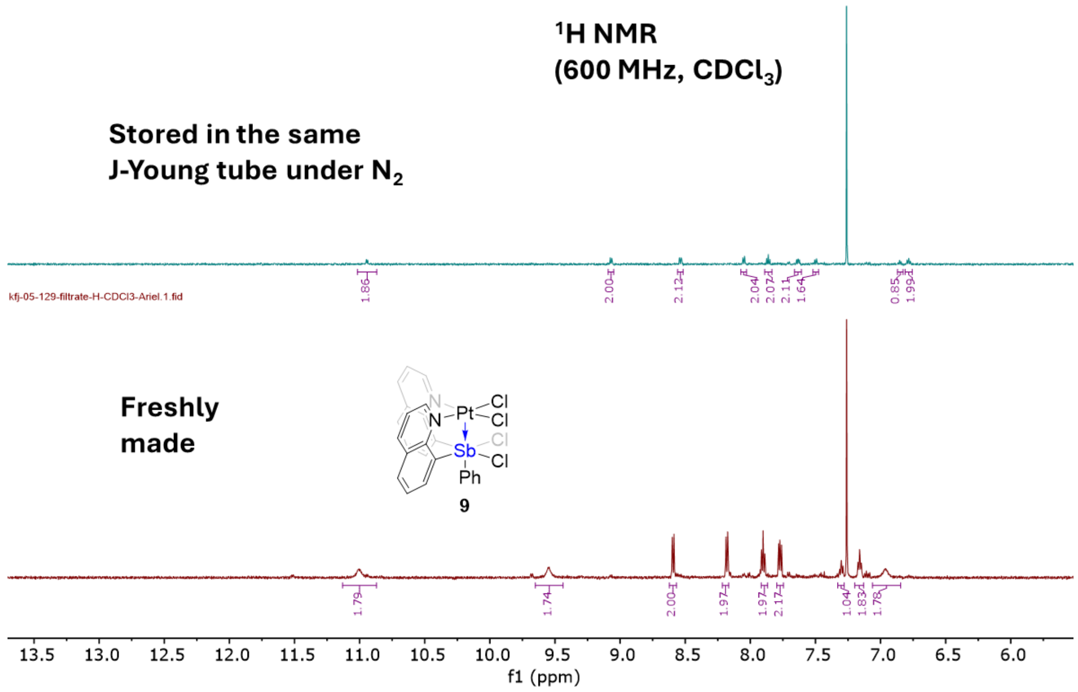

Figure S5. Complex **9** in $CDCl_3$ kept in a sealed J-Young tube under N_2.

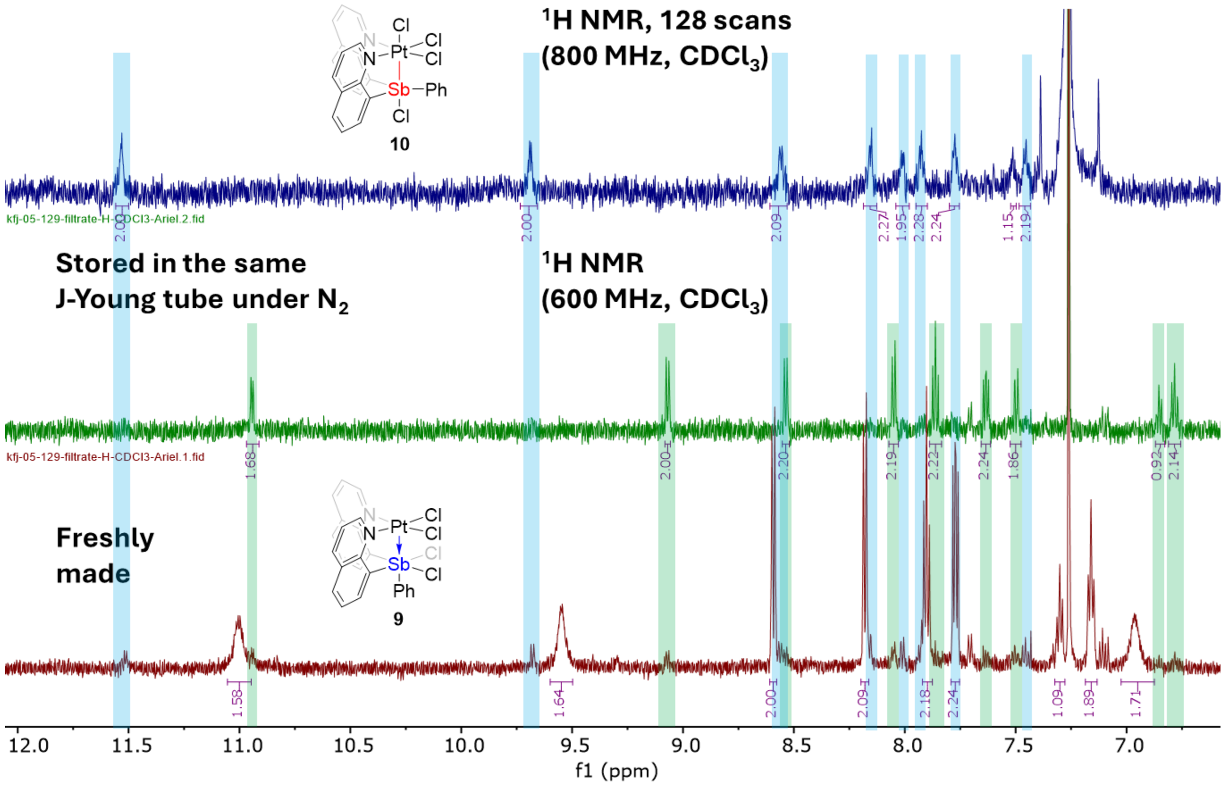

Figure S6. Comparison of ^{1}H NMR spectra of complex **9** and **10** in $CDCl_3$.

Efforts have been made to analyze complex **9** in the solid phase, which was expected to be more stable compared to **9** in solution phase. However, as shown in **Figure S7**, the powder XRD pattern of complex **9** stored in the dinitrogen filled glovebox matched that of complex **10**. This observation suggests that the conversion from complex **9** to **10** also occurs in the solid phase making it difficult to confirm the structure of complex **9**.

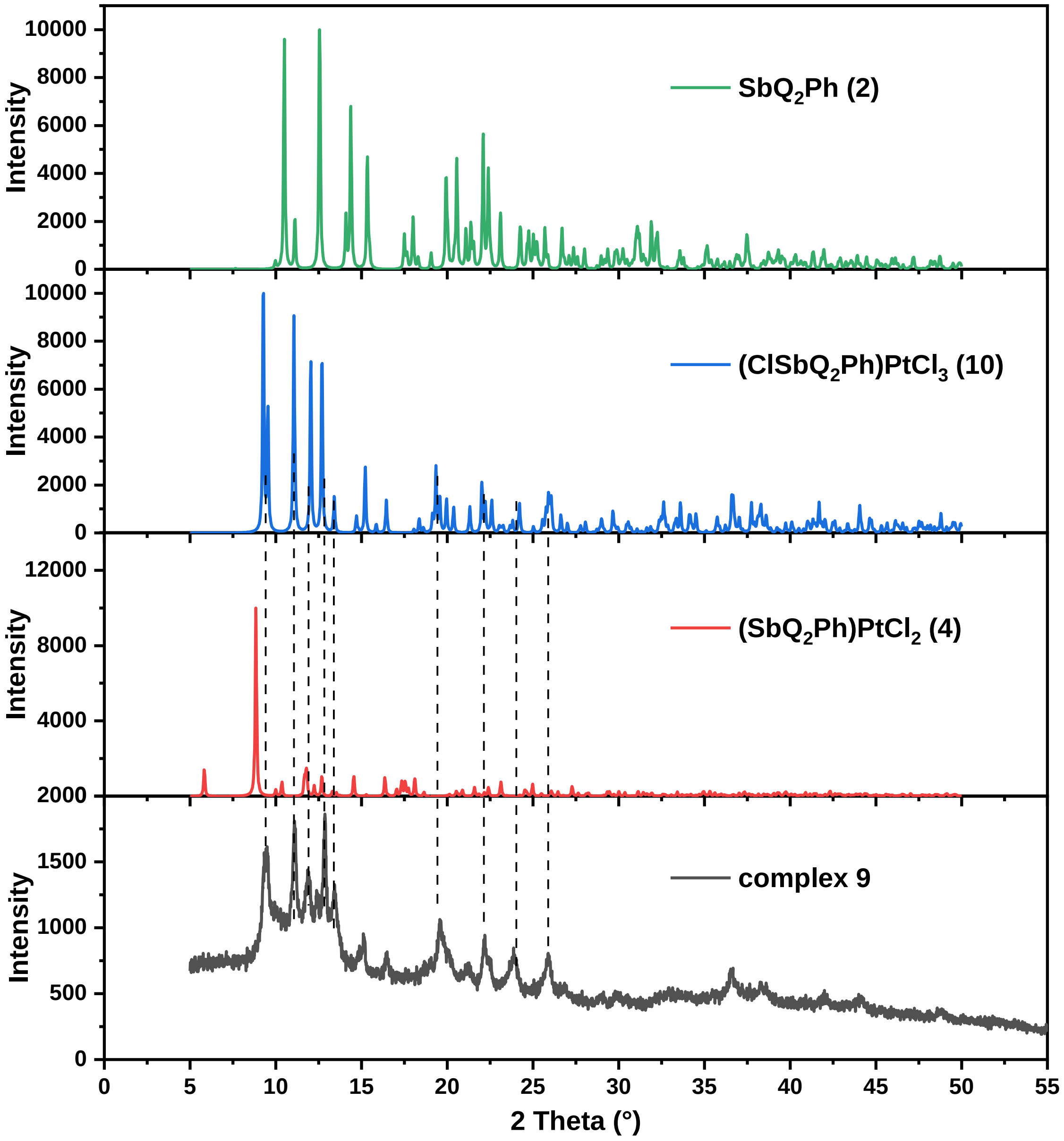

Figure S7. Powder XRD pattern of isolated crude complex **9** and other related complexes.

4. DFT Modeling of Chloride Transfer Between Pt and Sb.

Reacting complex **3** or **4** with $PhICl_2$ lead to the isolated products **8** or **10** with different coordination mode. To better understand this, we examined the relative energies of complex **7** versus **8** and complex **9** versus **10**. Despite a mixture of **7** and **8** formed from reaction of **3** with $PhICl_2$, there is a calculated 6.5 kcal·mol^{-1} energy preference (B2-PLYP/def2-TZVPD//M06/def2-SVP) for complex **7**. This could suggest that the mixture of complexes is the result of kinetic competition during their formation. Perhaps surprisingly, there is a reversal in relative energies with change from Q to a Ph ligand. Complex **10** was calculated to be 2.5 kcal·mol^{-1} more stable than **9** (**Scheme S3**). One possible explanation for the inversion of the relative energies is that the Ph group does not have the ability to have bridging coordination between Sb and Pt and therefore chloride cannot then form the ionic type bond with Pt as occurs in complex **7**.

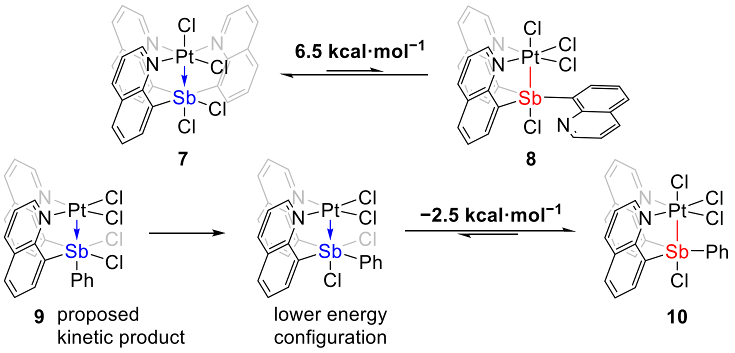

Scheme S3. DFT modeling of chloride transfer between Pt and Sb via B2-PLYP/def2-TZVPD//M06/def2-SVP.

5. Unexpected Doublets Observed in $^{13}C\{^1H\}$ NMR Spectra

In the obtained $^{13}C\{^1H\}$ NMR spectra of complexes **3**, **4** and **11**, doublets were observed in the most downfield $^{13}C\{^1H\}$ resonance for each complex (**Figure S8**, bottom). The presence of the downfield shifted ^1H resonances at > 11 ppm in the ^1H NMR spectra of those complexes led us to consider that the power of the ^1H decoupler for the $^{13}C\{^1H\}$ NMR experiments might not be sufficient for the downfield shifted ^1H resonances. To test this hypothesis, we changed the center of the ^1H decoupler field from 4.0 to 9.3 ppm and obtained another $^{13}C\{^1H\}$ NMR spectrum of complex **4** (**Figure S8**, top). As expected, the doublet observed in the bottom spectrum of **Figure S8** collapsed to a singlet, which is consistent with our speculation that the observed abnormal doublets in the $^{13}C\{^1H\}$ NMR spectra of complexes **3**, **4** and **11** were the result of incomplete ^1H decoupling of the proton resonances with chemical shifts > 11 ppm.

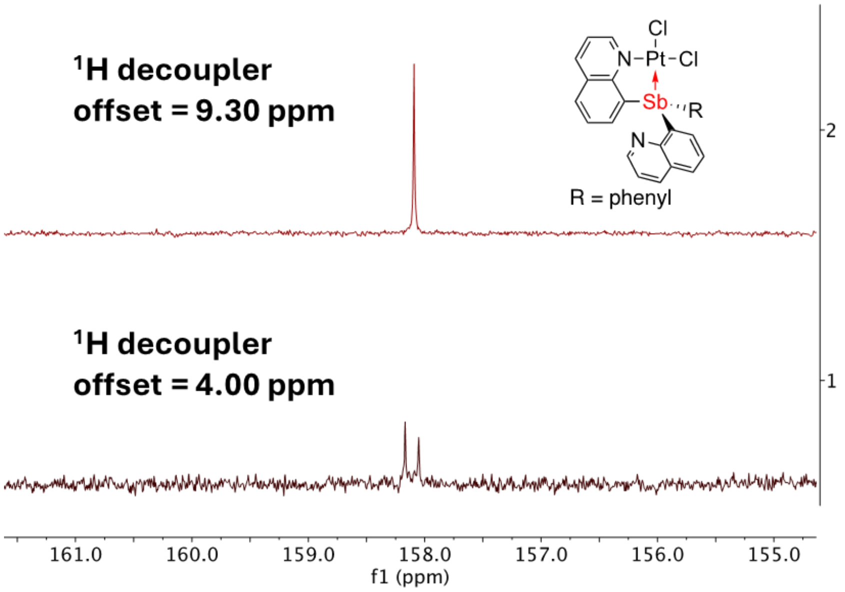

Figure S8. Comparison between $^{13}C\{^1H\}$ NMR spectra of complex **4** using different ^1H decoupling offset.

6. Reaction of Complex {(*o*-chloranil)SbQ$_3$}PtCl$_2$ (11) with MeOH

The reaction of complex **11** with MeOH generates {(o-chloranil)SbQ$_2$(OMe)}PtCl$_2$ (**13**).

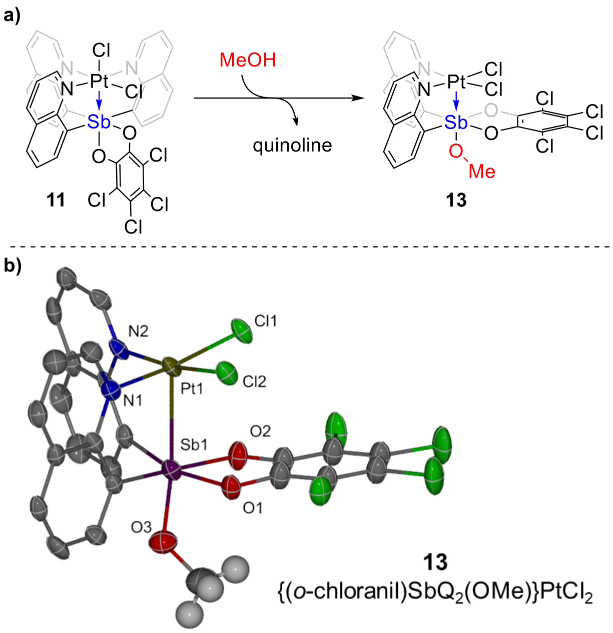

Scheme S4. a) Formation of {(*o*-chloranil)SbQ$_2$(OMe)}PtCl$_2$ (**13**) by reacting {(*o*-chloranil)SbQ$_3$}PtCl$_2$ (**11**) with MeOH, and **b)** the ORTEP (50% probability) of complex **13**.

Experimental Procedure:

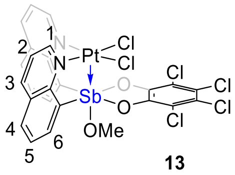

{(*o*-chloranil)SbQ$_2$OMe}PtCl$_2$ (**13**). In a sealed chamber containing diethyl ether, vials containing a solution of complex **11** in a mixture DCM and MeOH (1-to-1 by volume) were placed to facilitate vapor diffusion. Crystals of **13** were found in the vials.

7. Reaction of Complex 7 with HBF_4

Complex **7** was treated with $HBF_4 \cdot OEt_2$ in $CDCl_3$ in order to quaternize one of the quinoline nitrogen, which could potentially lead a chloride shift from Sb to Pt, forming a protonated analogue of complex **8**. However, all the proton resonances broadened out upon the addition of HBF_4 and later disappeared after heating at 40 °C overnight (**Figure S9**), suggesting the likely decomposition of complex **7** or the formation of insoluble salts.

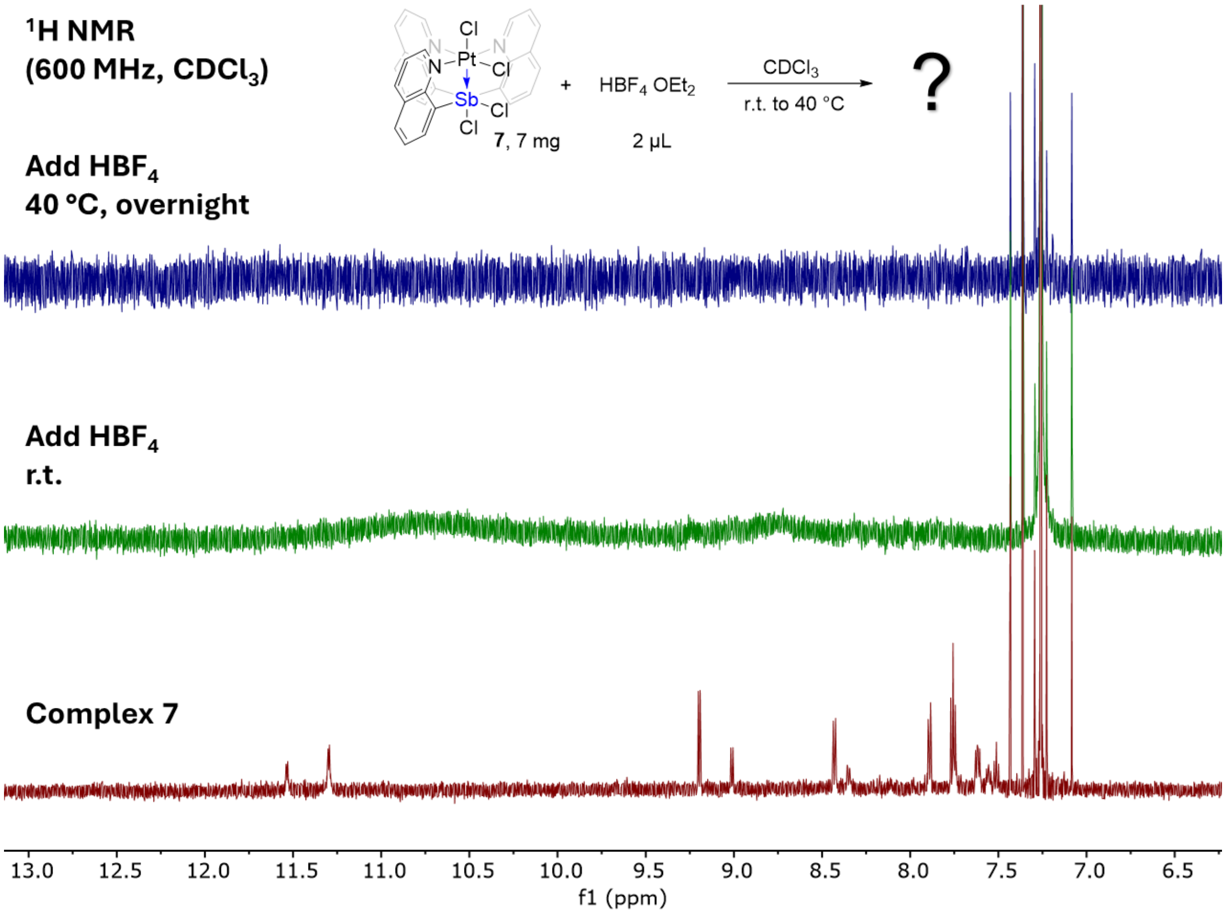

Figure S9. ^{1}H NMR spectra of the reaction of complex **7** with $HBF_4 \cdot OEt_2$ in $CDCl_3$.

8. EXSY experiments of Complexes 5 and 6

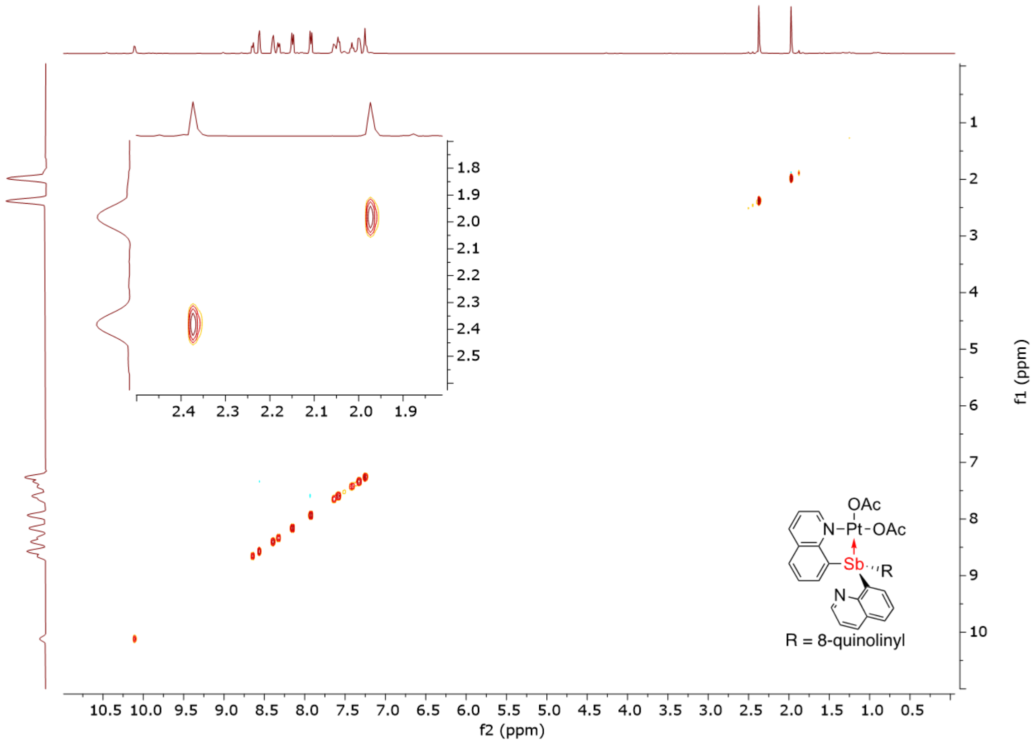

Figure S10. EXSY spectrum of complex **5**.

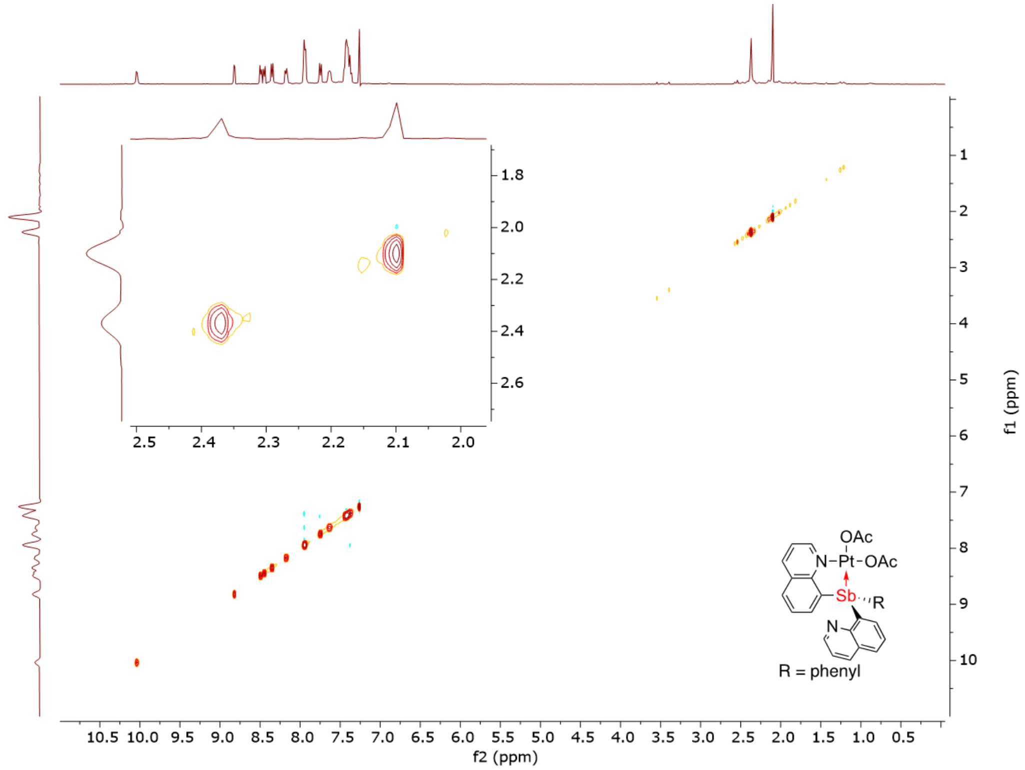

Figure S11. EXSY spectrum of complex **6**.

9. FT-IR Analysis of Complexes 5 and 6

FT-IR analysis was completed for complexes **5** and **6**. The strong absorptions at 1605 and 1566 cm^{-1} are attributed to ν_{asym}(COO), and the absorption at 1408 cm^{-1} is assigned to ν_{sym}(COO). The Δν {ν_{asym}(COO) - ν_{asym}(COO)} are 197 and 158 cm^{-1} for complex **5** and 194 and 158 cm^{-1} for **6**. The absorption at 1605 cm^{-1} could be partially attributed to aromatic C=C stretching. Previous studies have tried to correlate the acetate bonding mode with the Δν for which Δν in the range of 164–171 cm^{-1} is assigned to ionic acetate while larger values suggest monodentate acetate and smaller values indicate a bidentate or bridging acetate.[4-5] Based on this interpretation, the observed Δν values of complexes **5** and **6** are consistent with one monodentate acetate and acetate in a bridging mode.

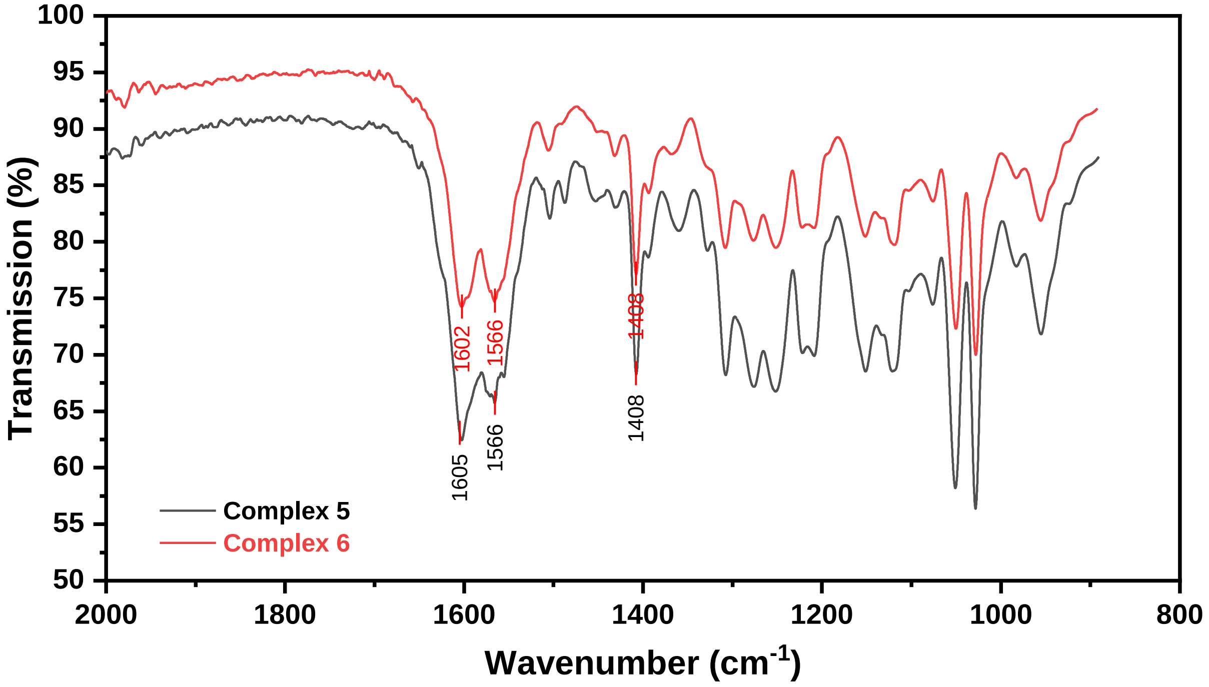

Figure S12. FT-IR spectrum of complex **5** and **6**.

10. NMR Spectra

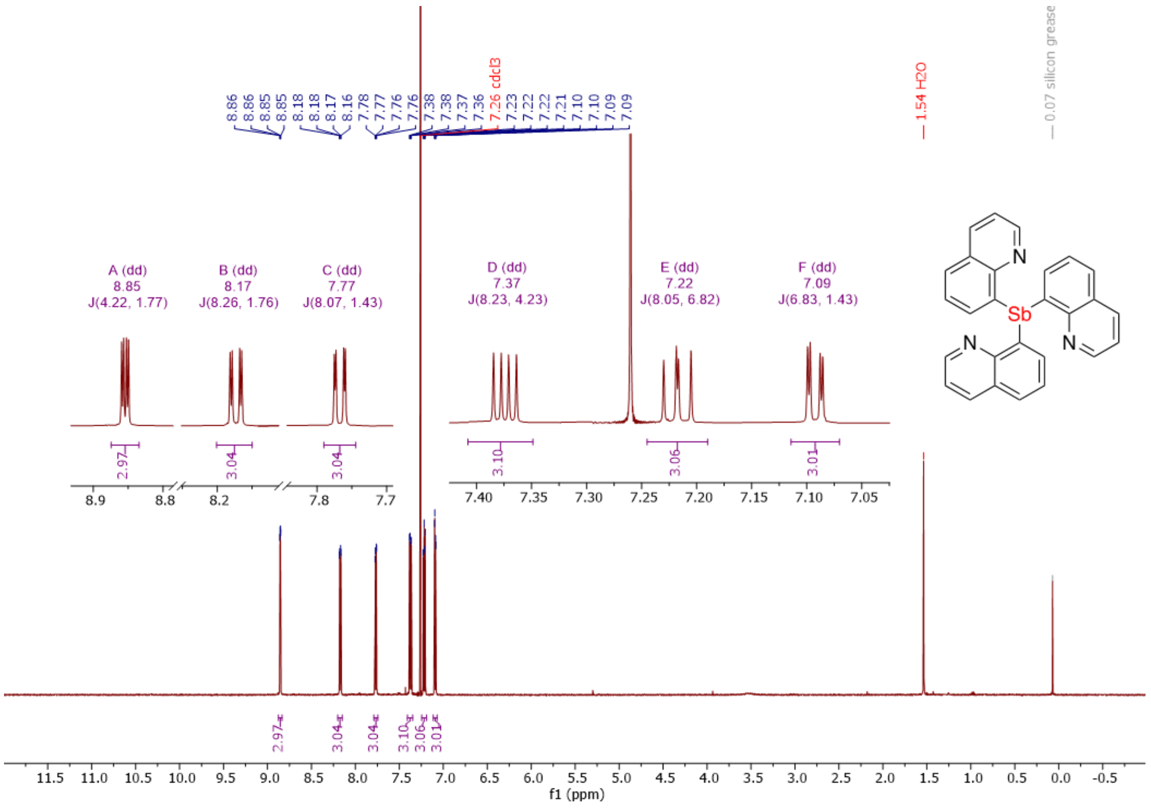

Figure S13. ^{1}H NMR spectrum of SbQ_3 (**1**) (600 MHz, $CDCl_3$). *Note:* water and silica grease are present in the spectrum.

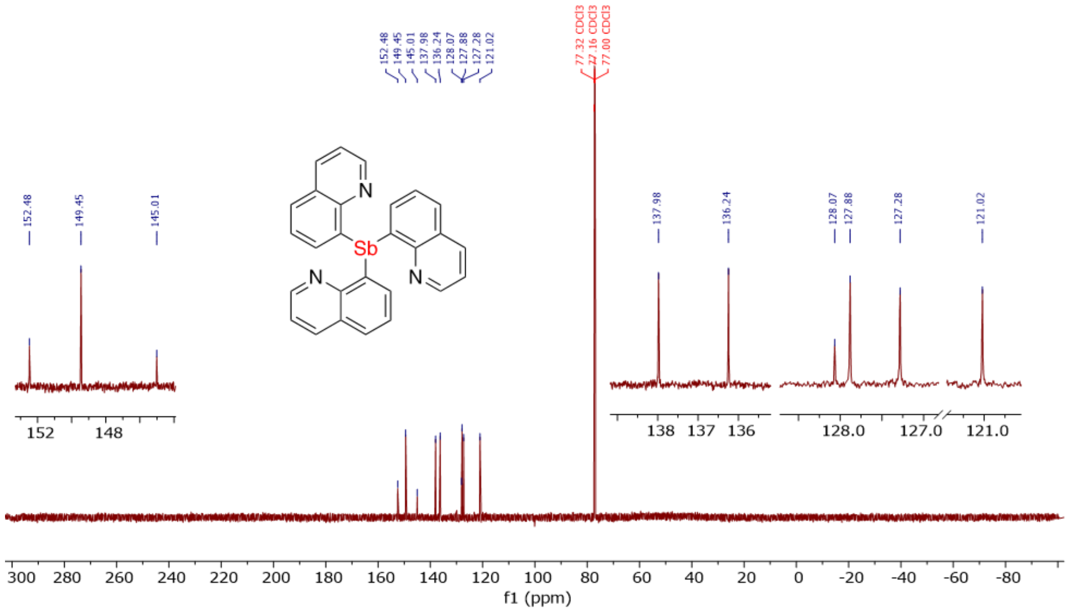

Figure S14. $^{13}C\{^{1}H\}$ NMR spectrum of SbQ_3 (**1**) (201 MHz, $CDCl_3$).

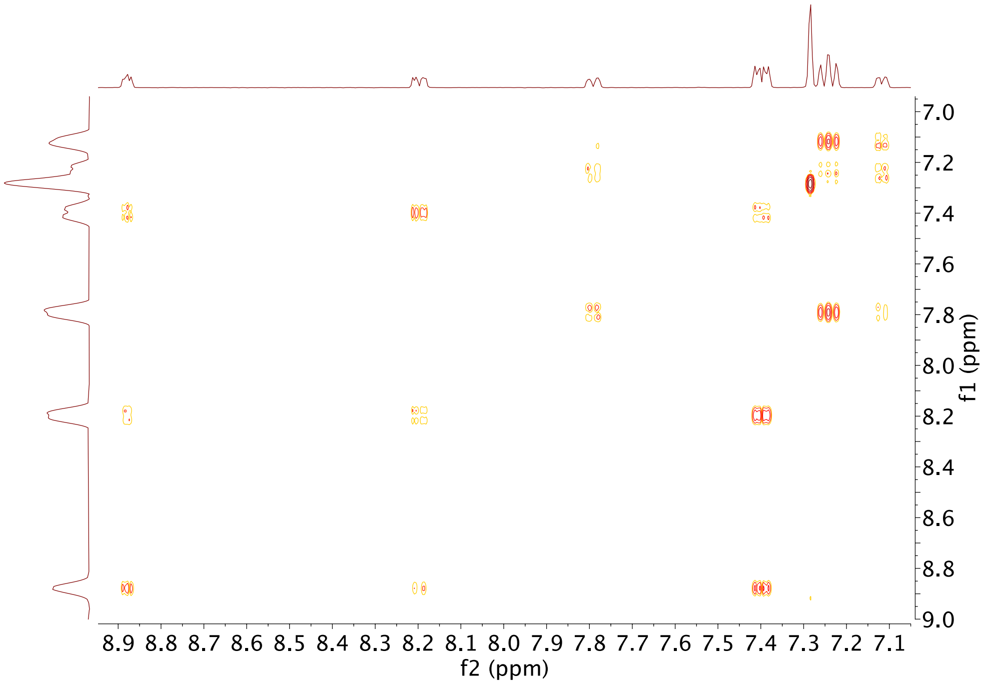

Figure S15. COSY spectrum of SbQ_3 (**1**) (400 MHz, $CDCl_3$).

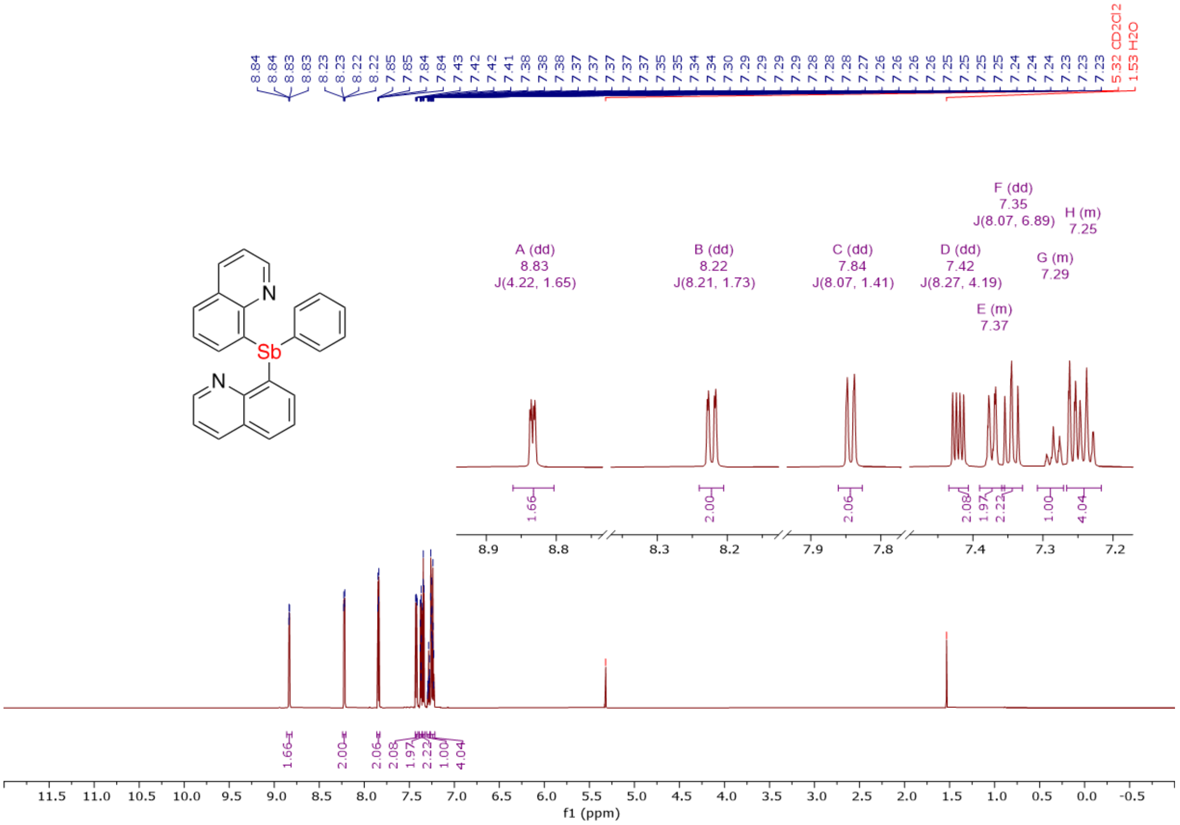

Figure S16. ^{1}H NMR spectrum of SbQ_2Ph (**2**) (800 MHz, CD_2Cl_2). *Note:* water is present in the spectrum.

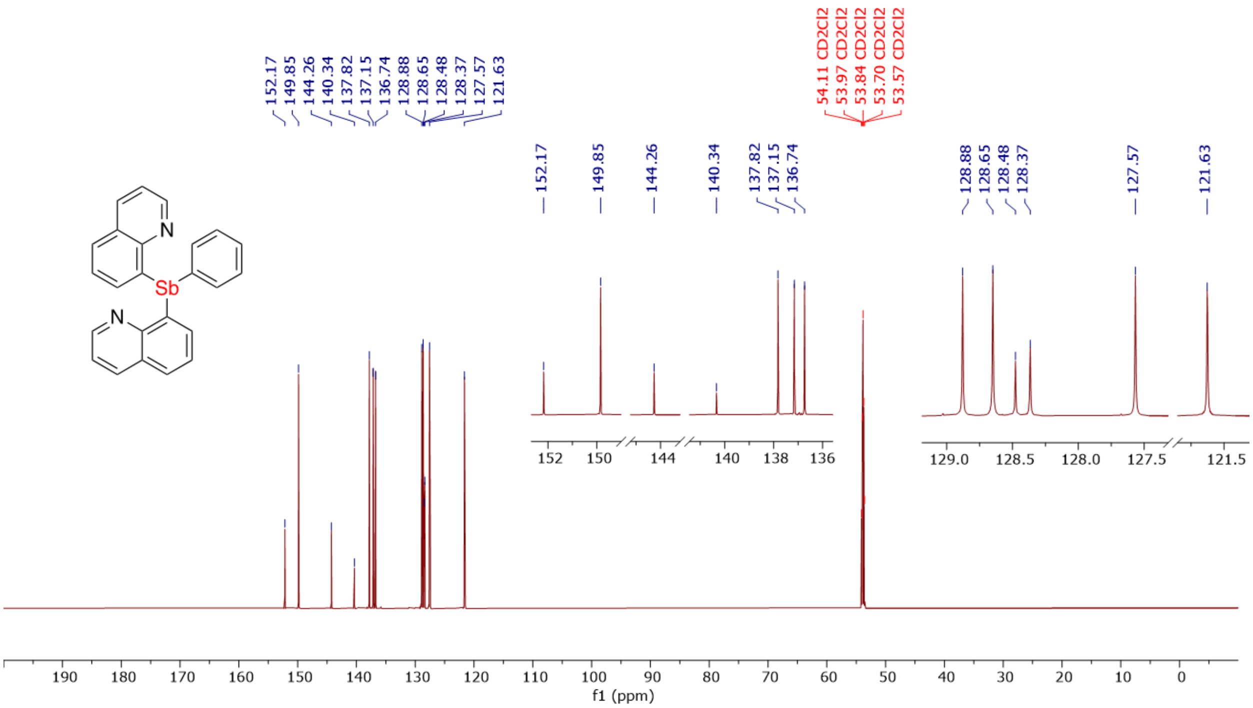

Figure S17. $^{13}C\{^{1}H\}$ NMR spectrum of SbQ_2Ph (**2**) (201 MHz, CD_2Cl_2).

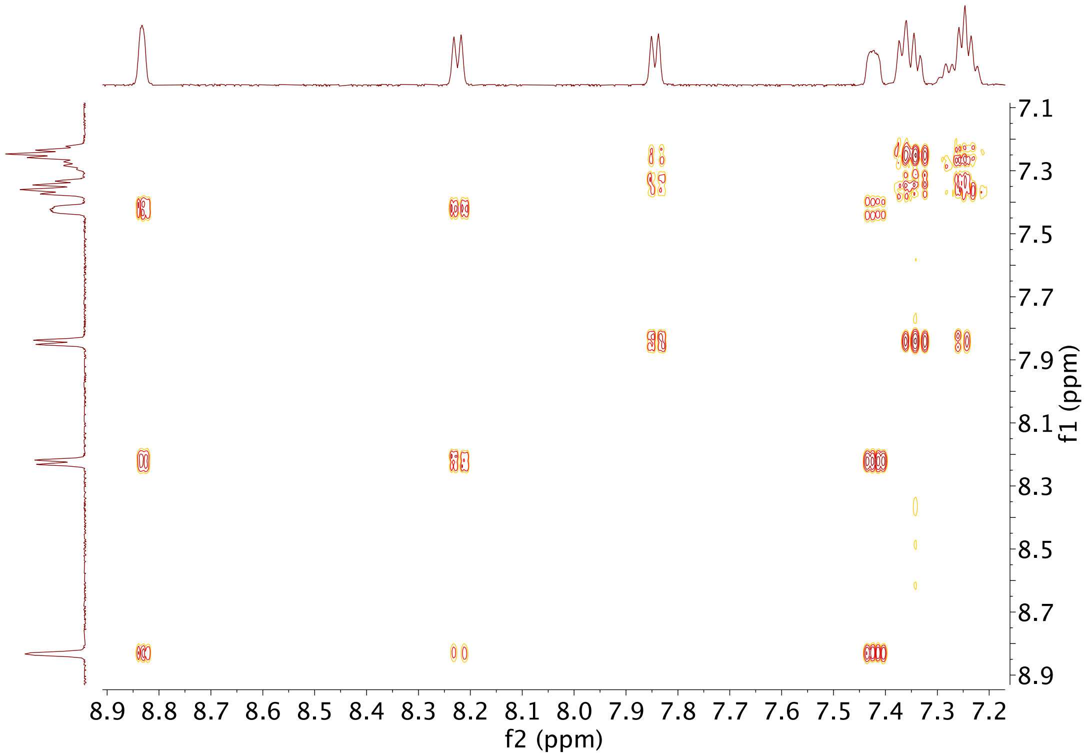

Figure S18. COSY spectrum of SbQ_2Ph (**2**) (400 MHz, CD_2Cl_2).

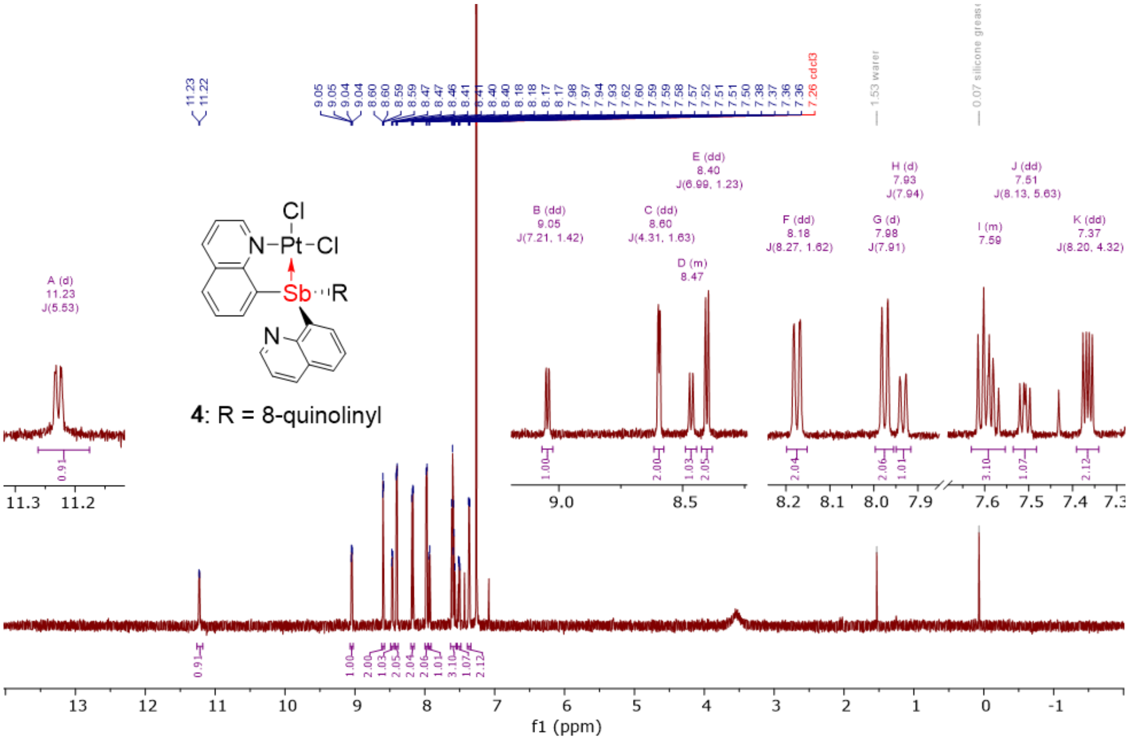

Figure S19. ^{1}H NMR spectrum of (SbQ$_3$)PtCl$_2$ (**3**) (600 MHz, CDCl$_3$). *Note:* water and silica grease are present in the spectrum.

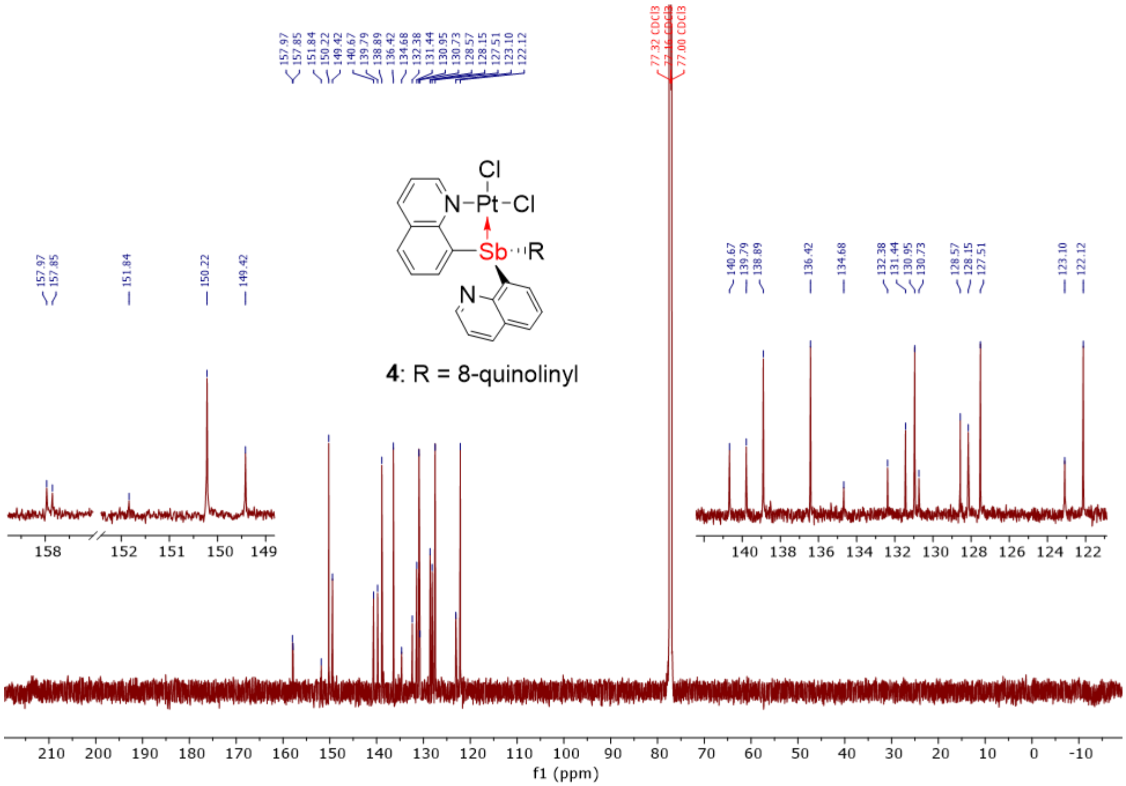

Figure S20. ^{13}C{^{1}H} NMR spectrum of (SbQ$_3$)PtCl$_2$ (**3**) (201 MHz, CDCl$_3$).

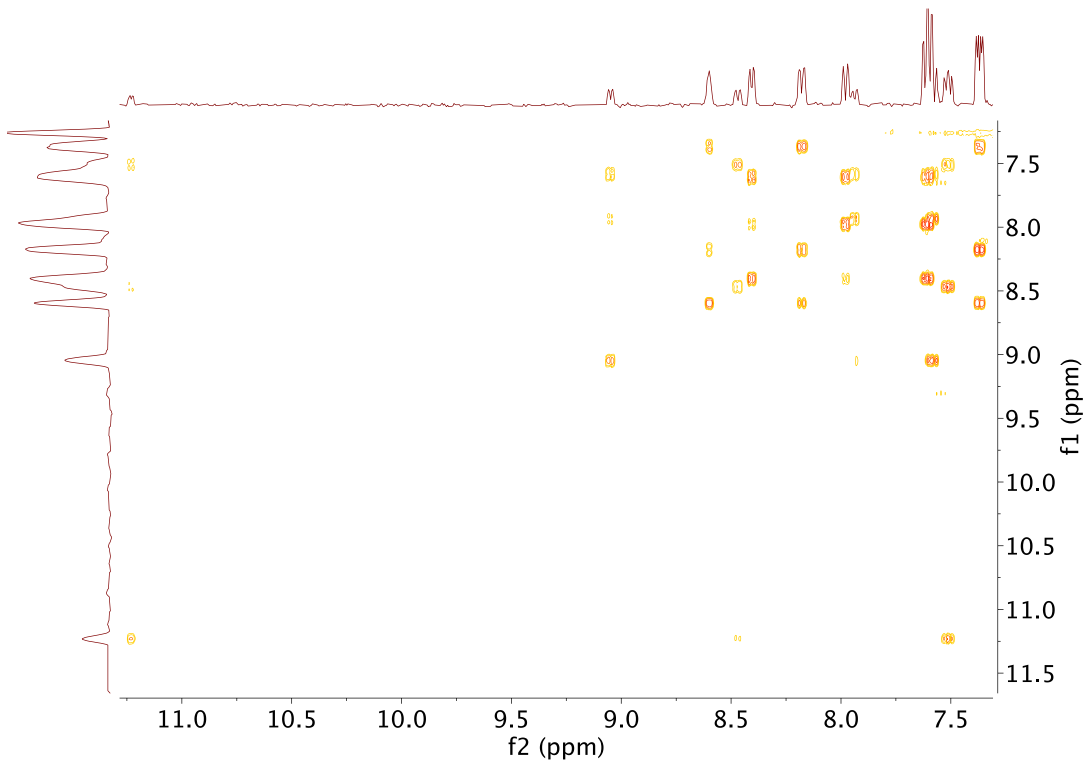

Figure S21. COSY spectrum of $(SbQ_3)PtCl_2$ (**3**) (400 MHz, $CDCl_3$).

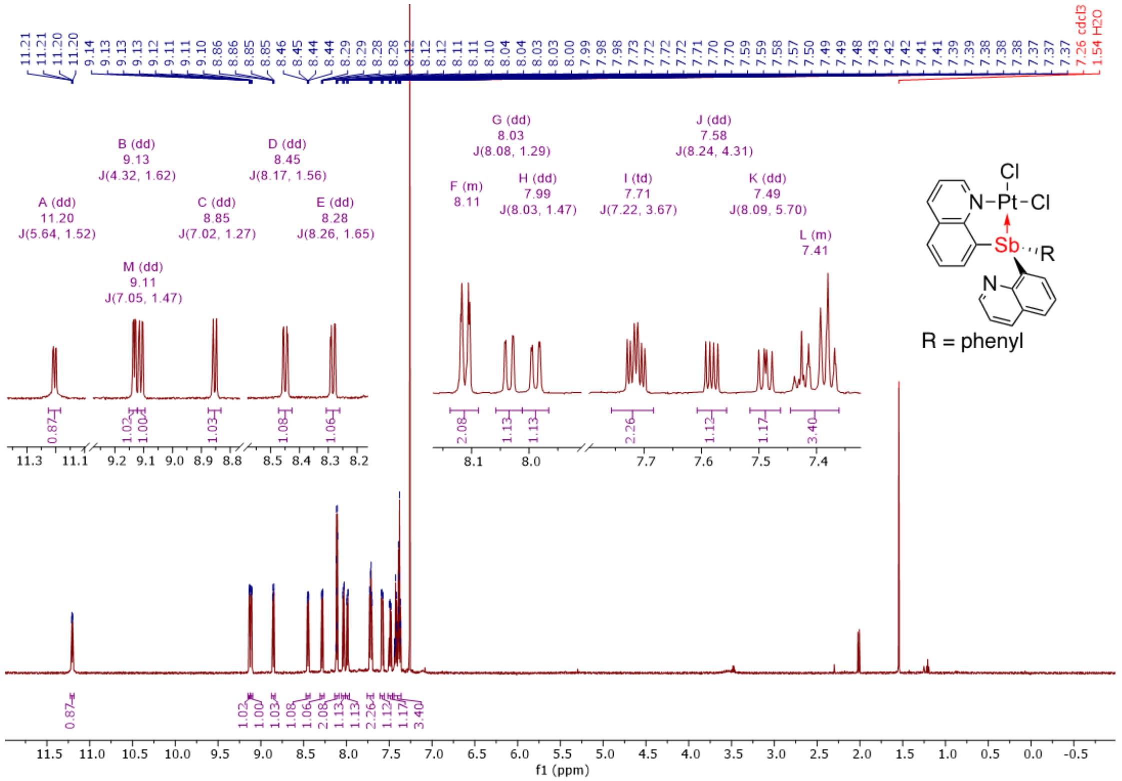

Figure S22. ^{1}H NMR spectrum of $(SbQ_2Ph)PtCl_2$ (**4**) (600 MHz, $CDCl_3$). *Note:* diethyl ether, MeCN and water are present in the spectrum.

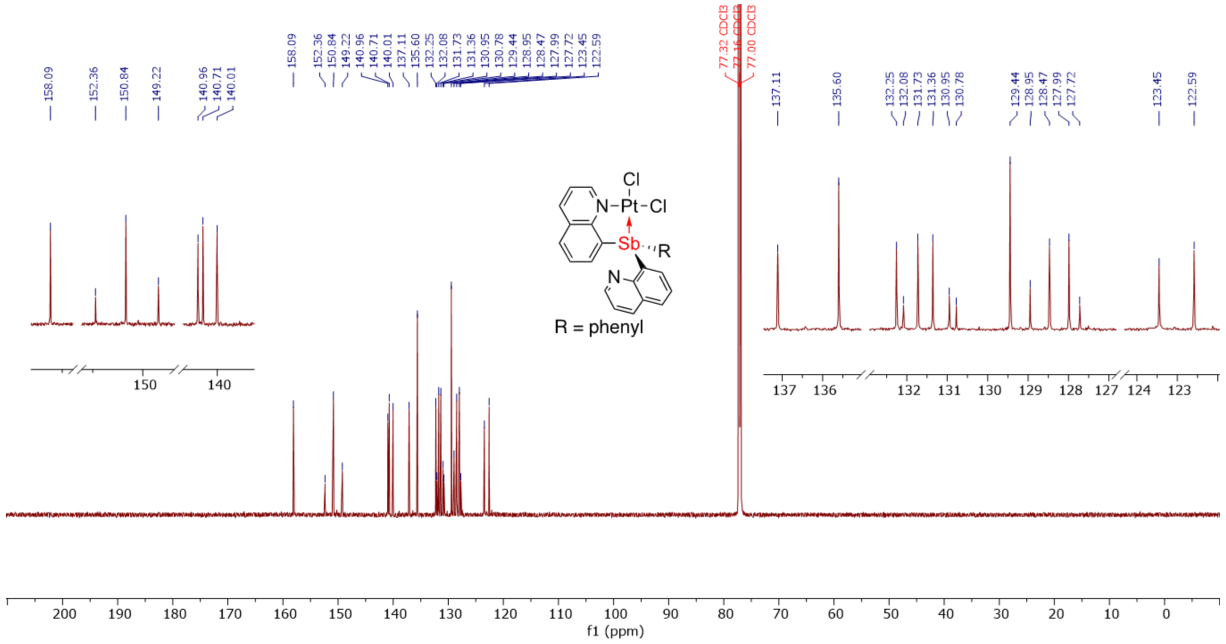

Figure S23. $^{13}C\{^{1}H\}$ NMR spectrum of $(SbQ_2Ph)PtCl_2$ (**4**) (201 MHz, $CDCl_3$).

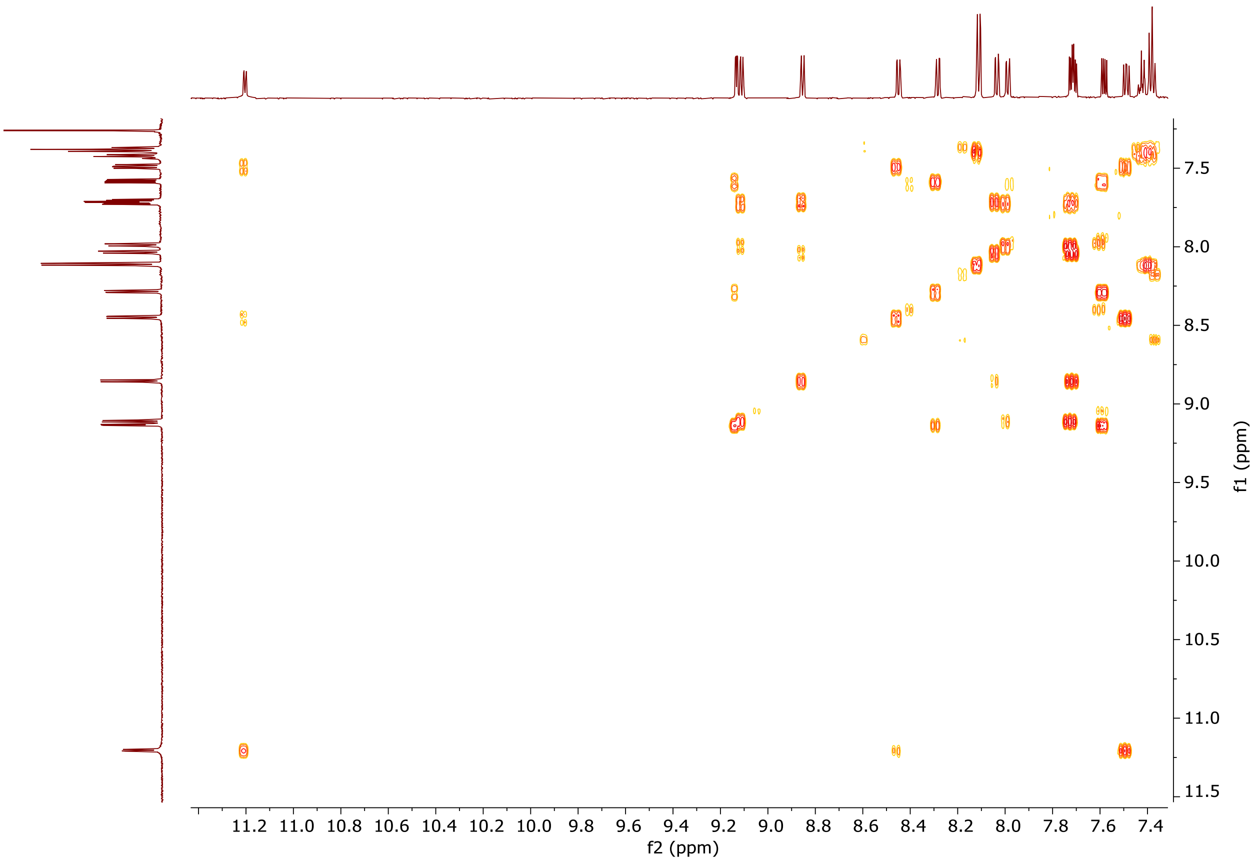

Figure S24. COSY spectrum of $(SbQ_2Ph)PtCl_2$ (**4**) (400 MHz, $CDCl_3$).

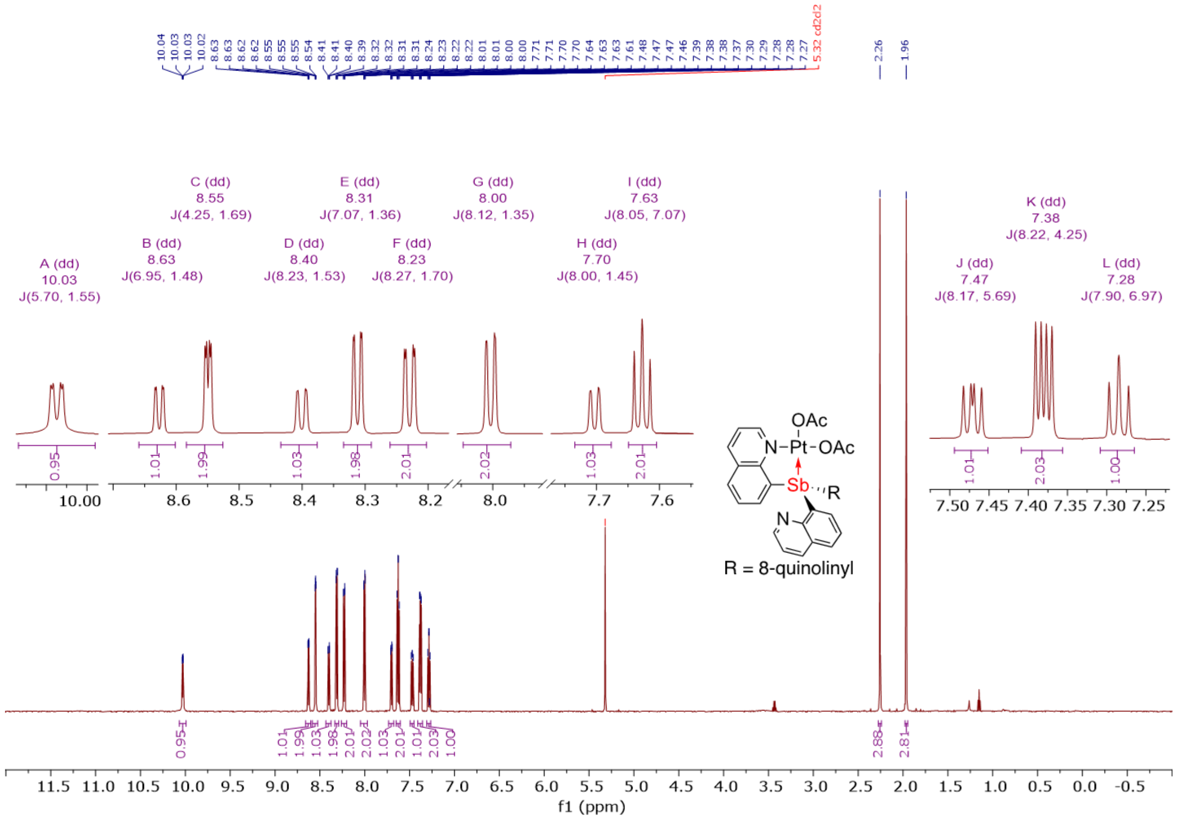

Figure S25. ^{1}H NMR spectrum of (SbQ$_3$)Pt(OAc)$_2$ (**5**) (600 MHz, CD$_2$Cl$_2$). *Note:* diethyl ether is present in the spectrum.

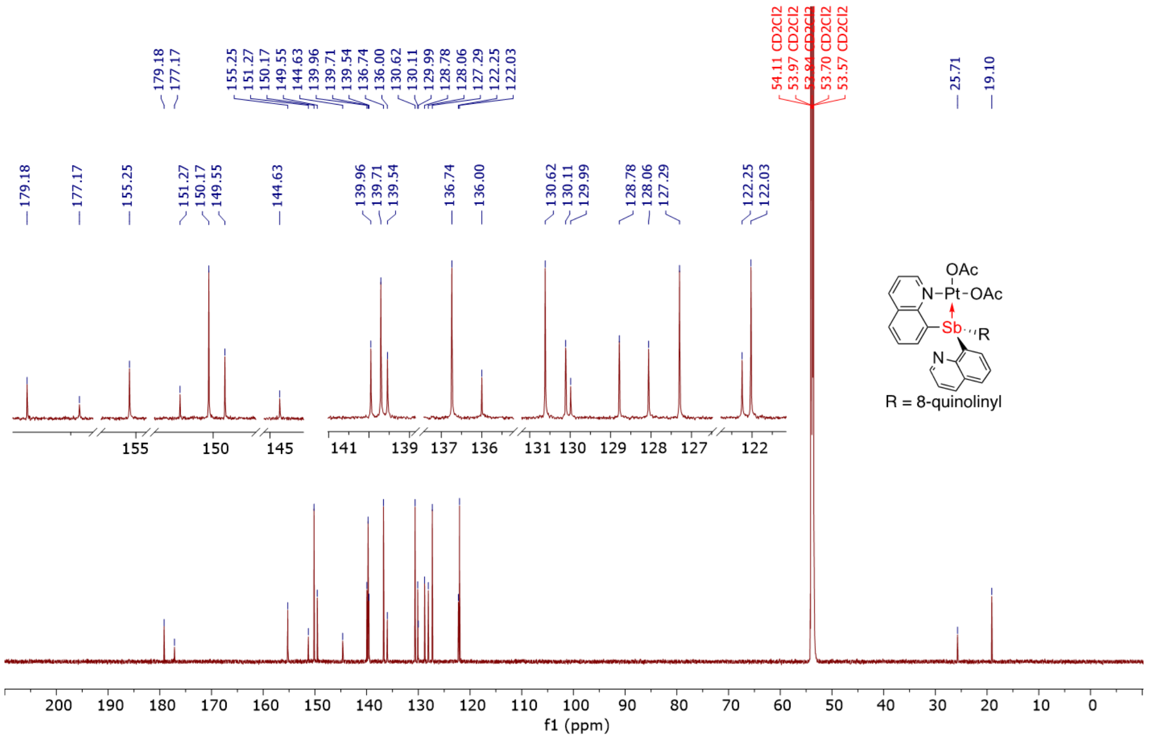

Figure S26. ^{13}C{^{1}H} NMR spectrum of (SbQ$_3$)Pt(OAc)$_2$ (**5**) (201 MHz, CD$_2$Cl$_2$).

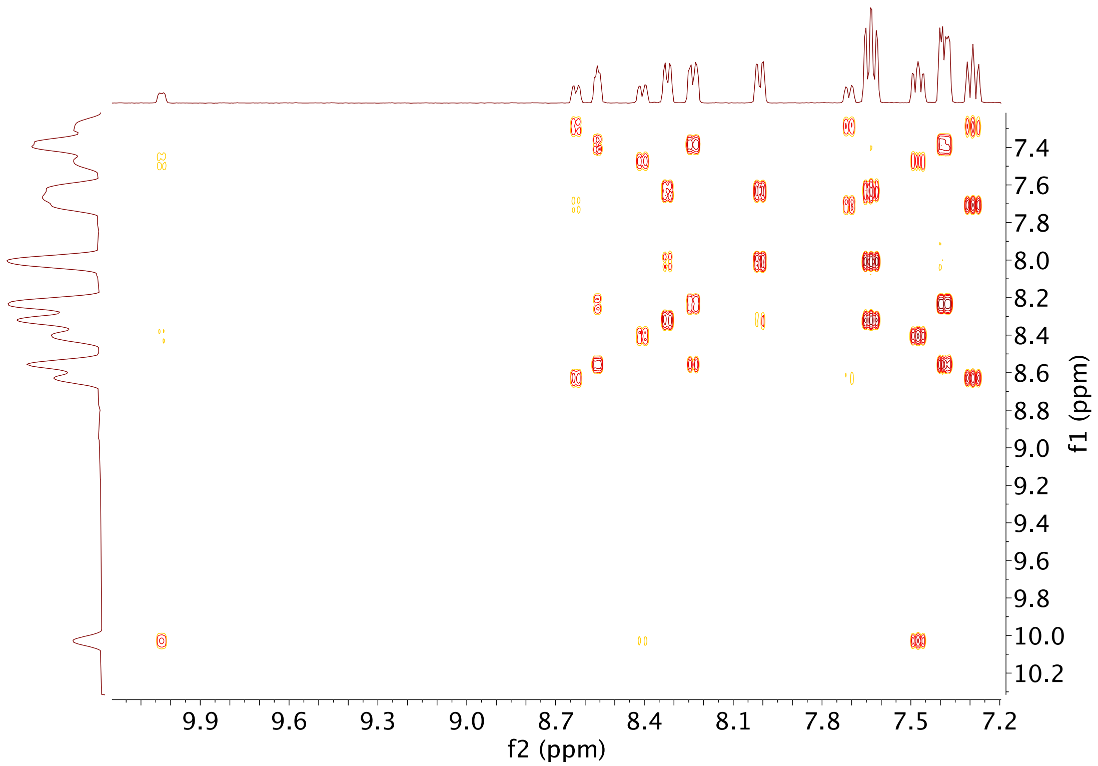

Figure S27. COSY spectrum of $(SbQ_3)Pt(OAc)_2$ (**5**) (400 MHz, CD_2Cl_2).

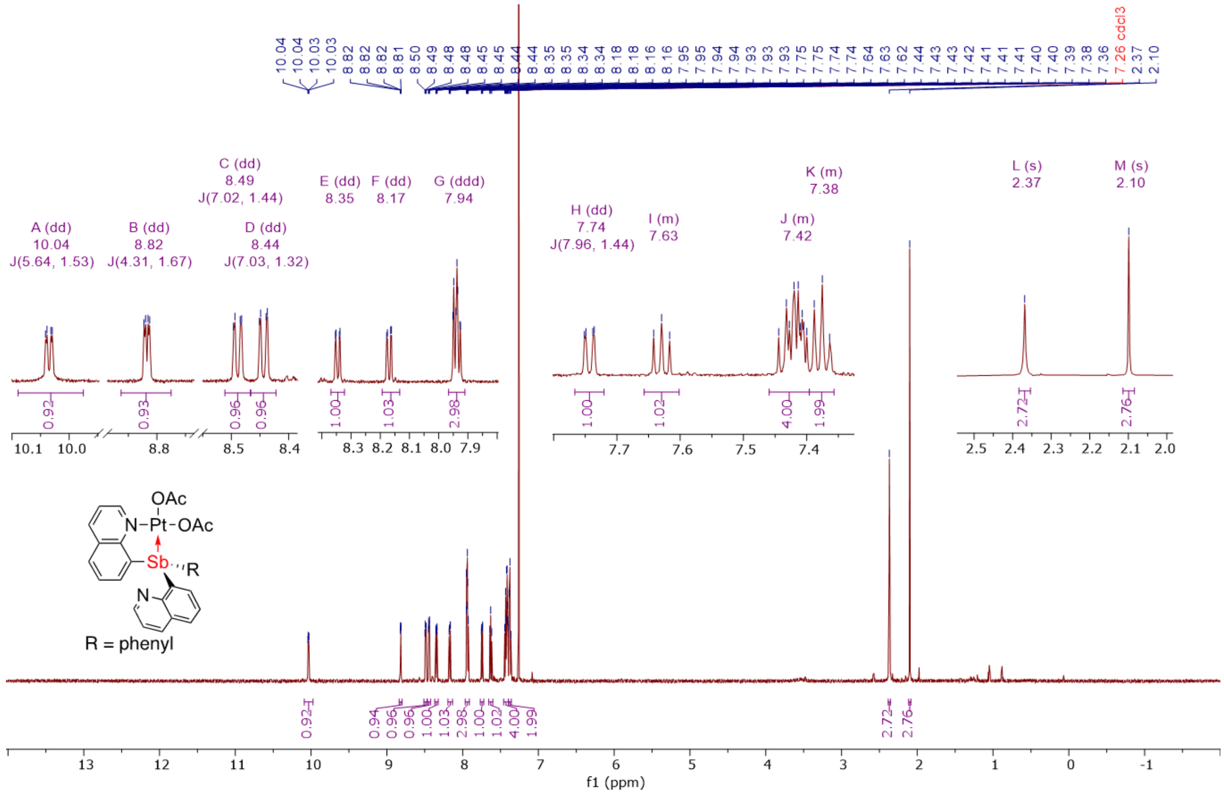

Figure S28. ^{1}H NMR spectrum of $(SbQ_2Ph)Pt(OAc)_2$ (**6**) (600 MHz, $CDCl_3$).

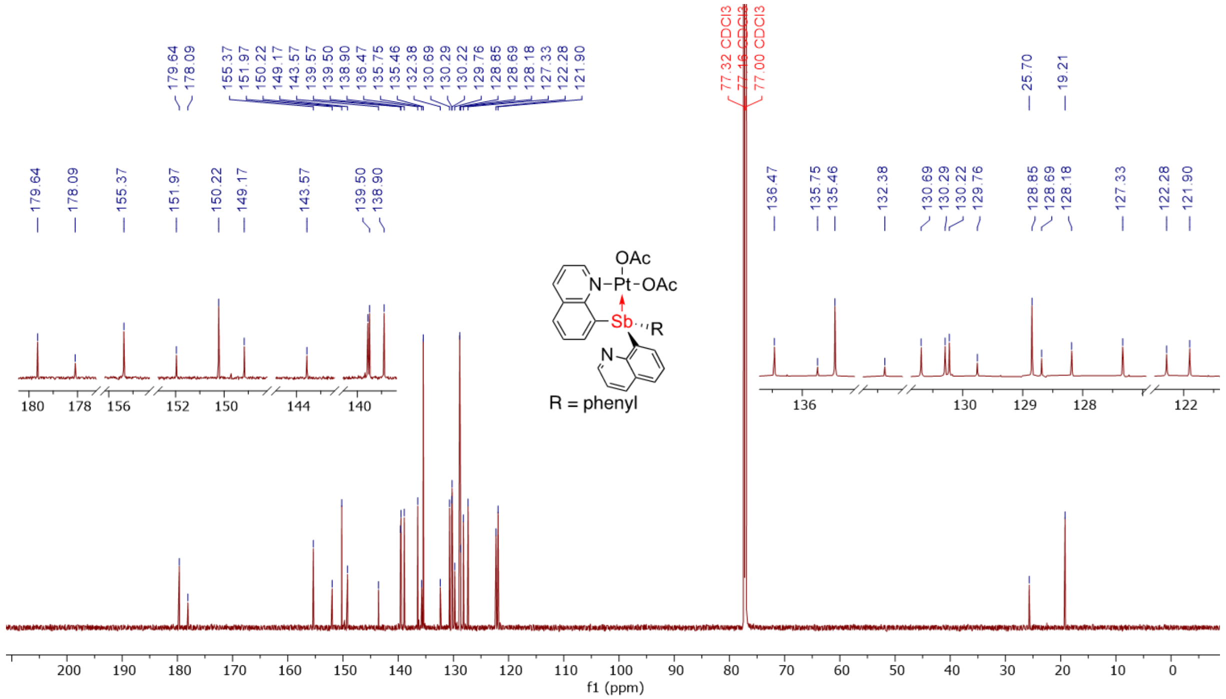

Figure S29. $^{13}C\{^{1}H\}$ NMR spectrum of $(SbQ_2Ph)Pt(OAc)_2$ (**6**) (201 MHz, $CDCl_3$).

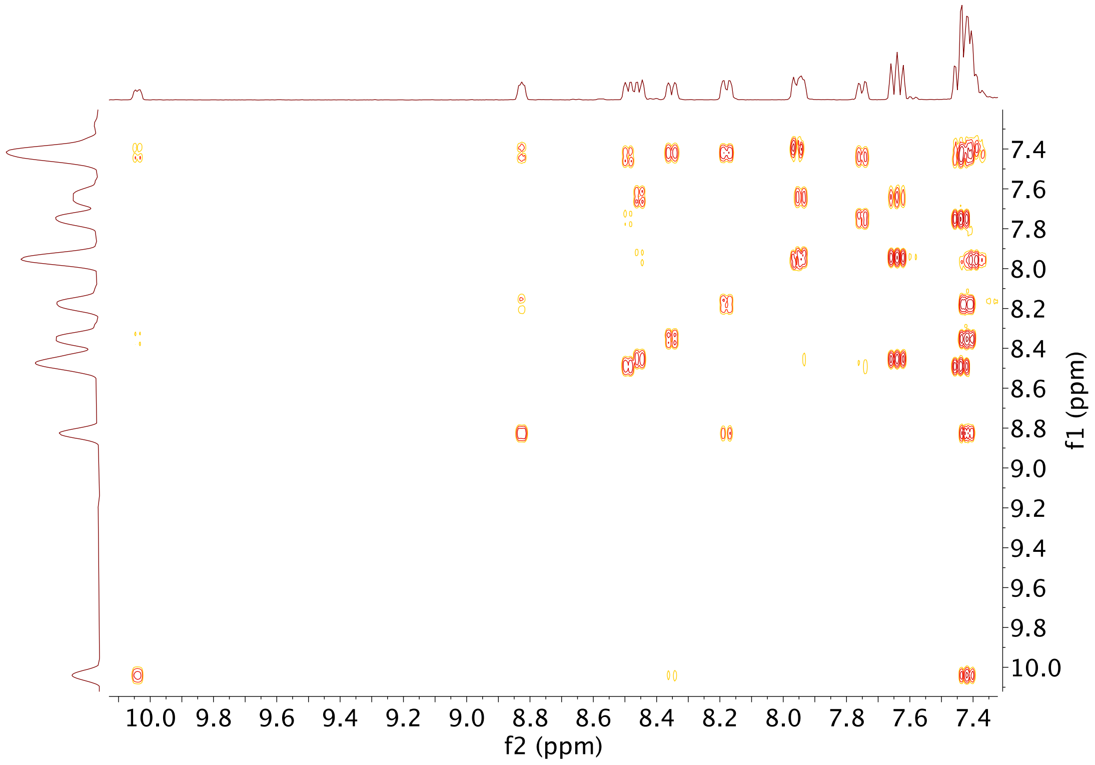

Figure S30. COSY spectrum of $(SbQ_2Ph)Pt(OAc)_2$ (**6**) (400 MHz, $CDCl_3$).

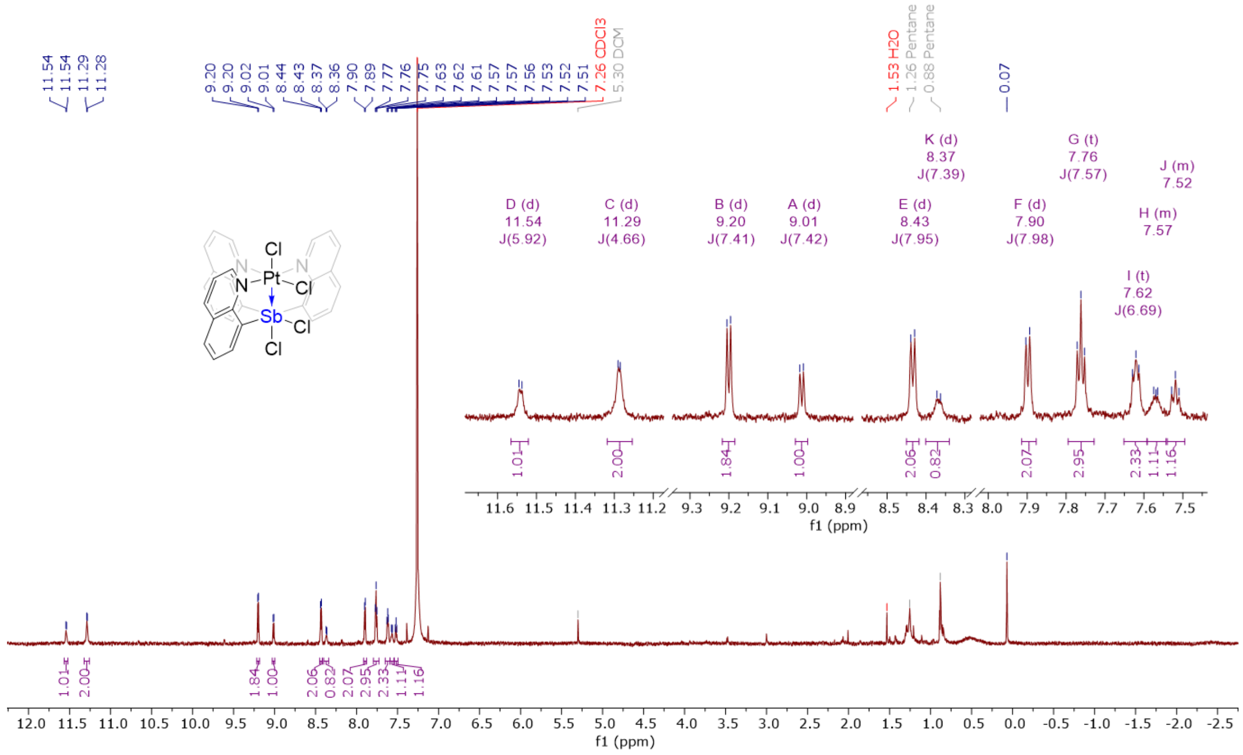

Figure S31. ^{1}H NMR spectrum of $(Cl_2SbQ_3)PtCl_2$ (**7**) (600 MHz, CD_2Cl_2). *Note:* solvent impurities are present with high intensity due to the poor solubility of complex **7**.

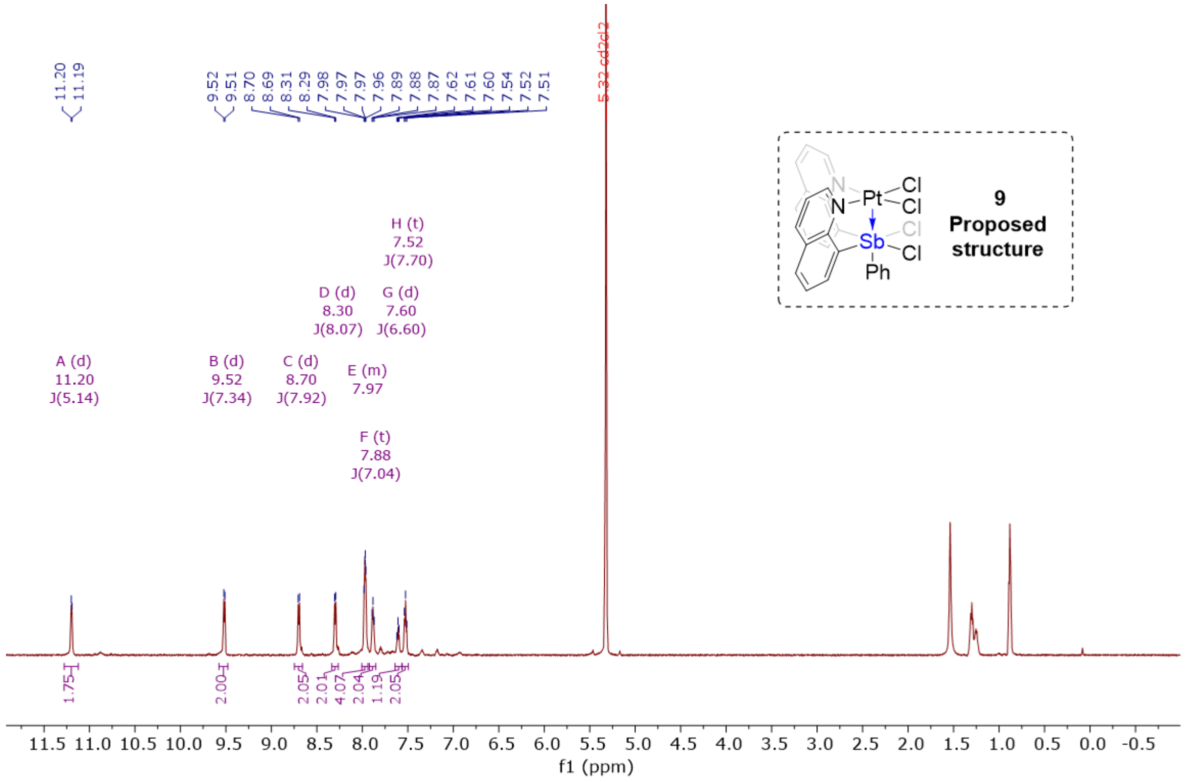

Figure S32. ^{1}H NMR spectrum of $(Cl_2SbQ_2Ph)PtCl_2$ (**9**) (600 MHz, CD_2Cl_2).

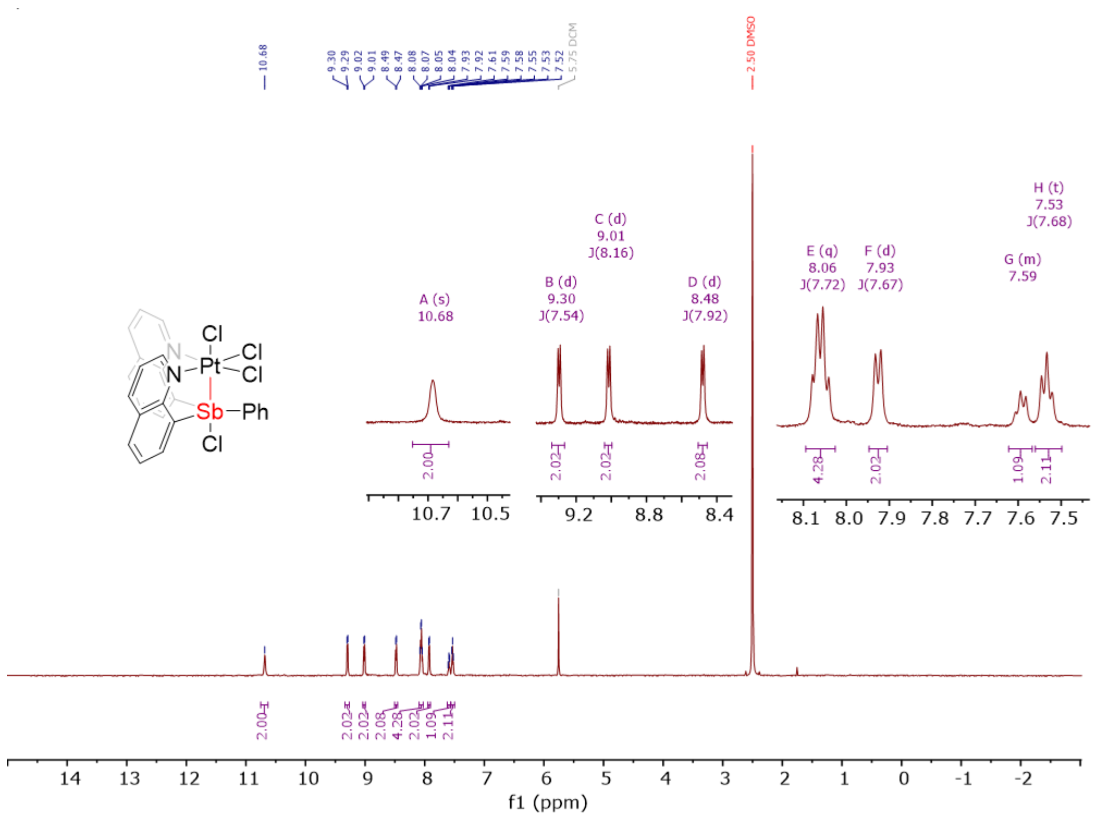

Figure S33. ^{1}H NMR spectrum of (ClSbQ$_2$Ph)PtCl$_3$ (**10**) (600 MHz, DMSO-d_6). *Note:* dichloromethane is present in the spectrum.

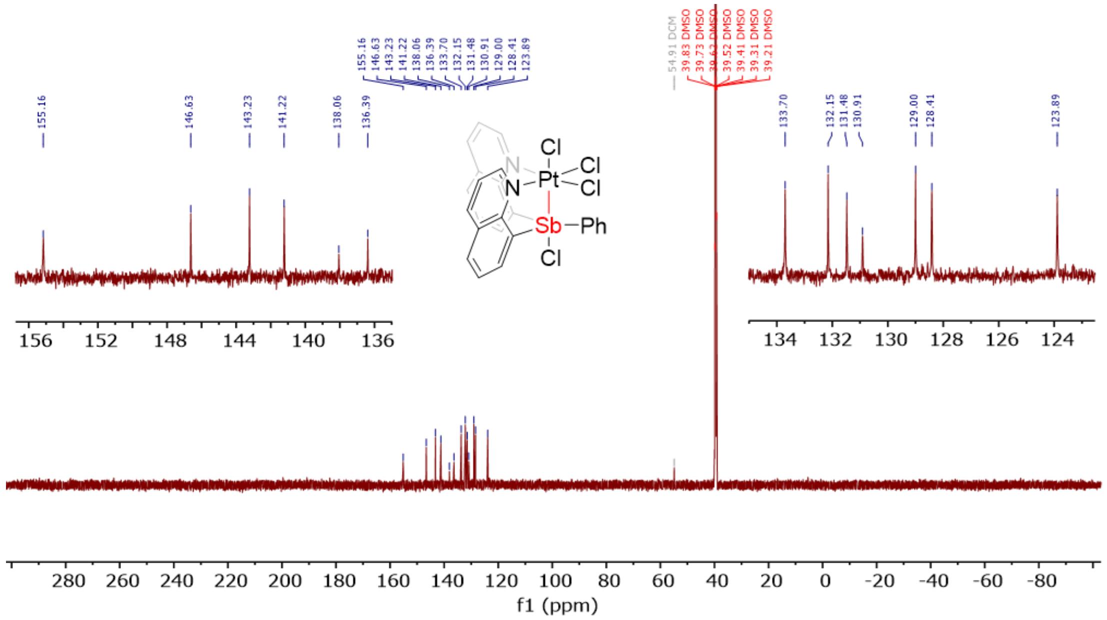

Figure S34. ^{13}C{^{1}H} NMR spectrum of (ClSbQ$_2$Ph)PtCl$_3$ (**10**) (201 MHz, DMSO-d_6). *Note:* dichloromethane is present in the spectrum

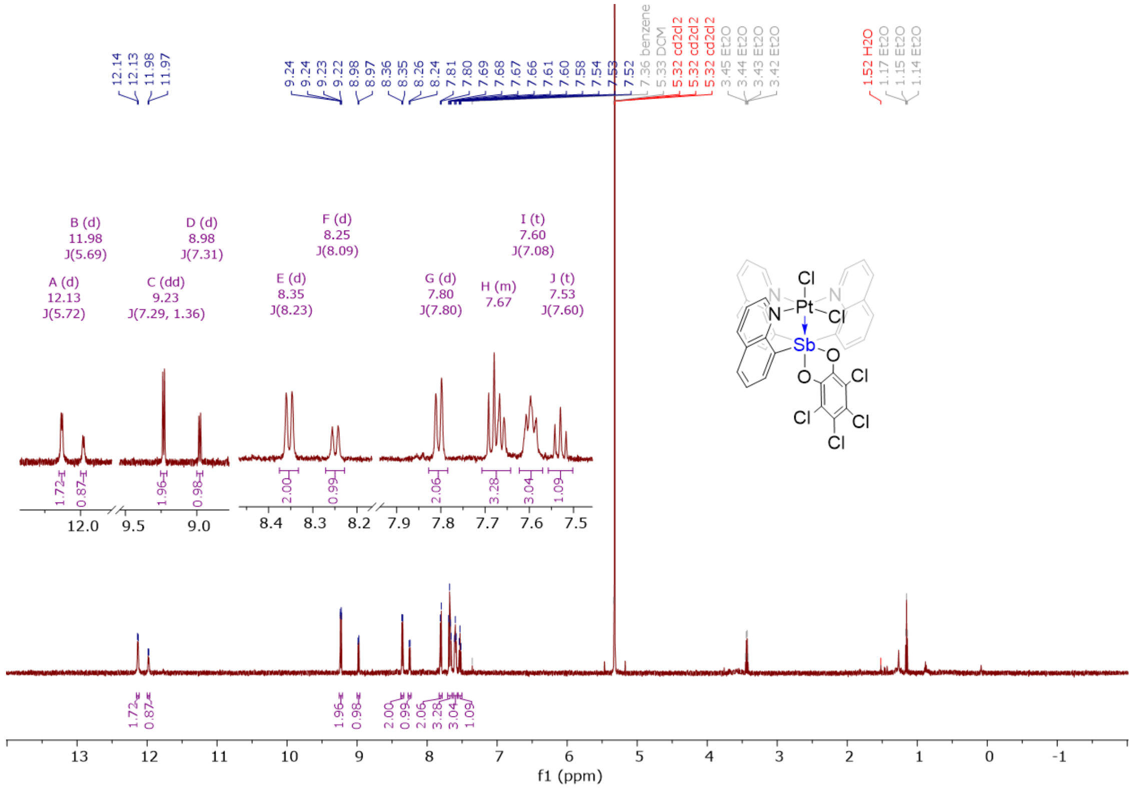

Figure S35. ^{1}H NMR spectrum of {(*o*-chloranil)SbQ$_3$}PtCl$_2$ (**11**) (600 MHz, CD_2Cl_2). *Note:* diethyl ether is present in the spectrum

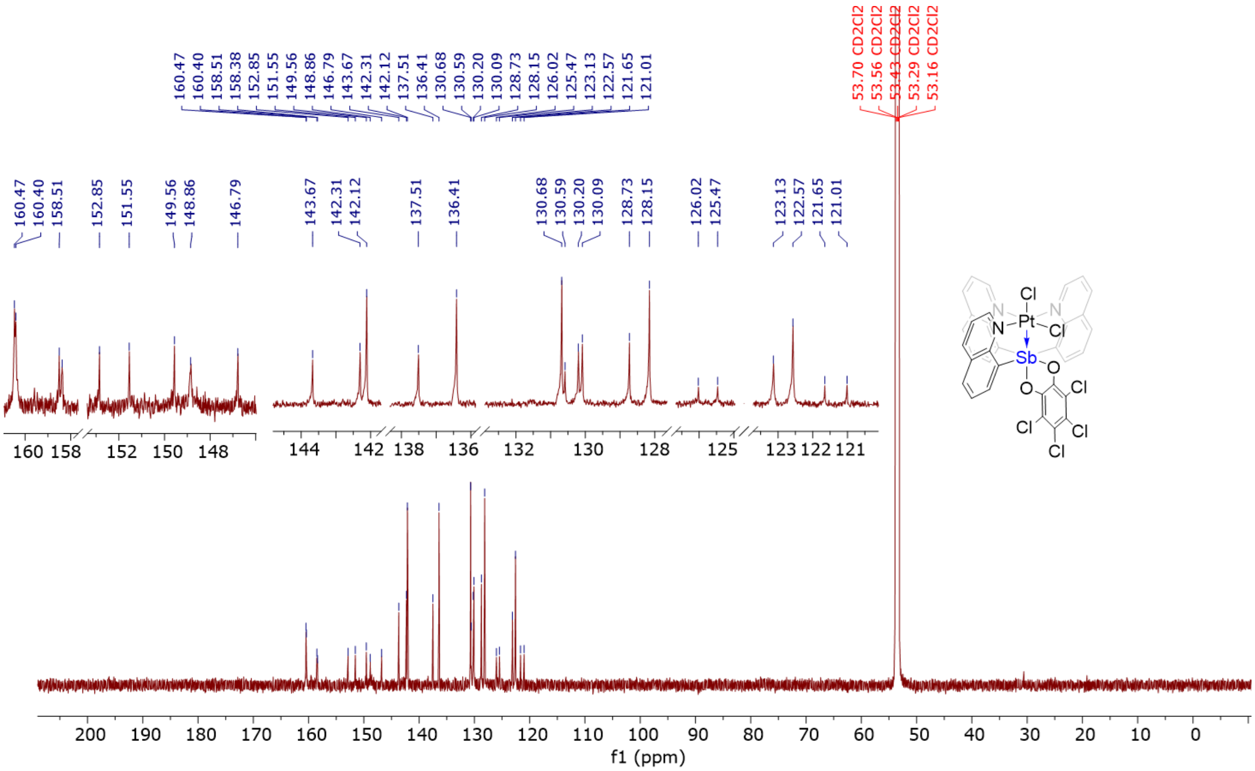

Figure S36. ^{13}C{^{1}H} NMR spectrum of {(*o*-chloranil)SbQ$_3$}PtCl$_2$ (**11**) (201 MHz, CD_2Cl_2).

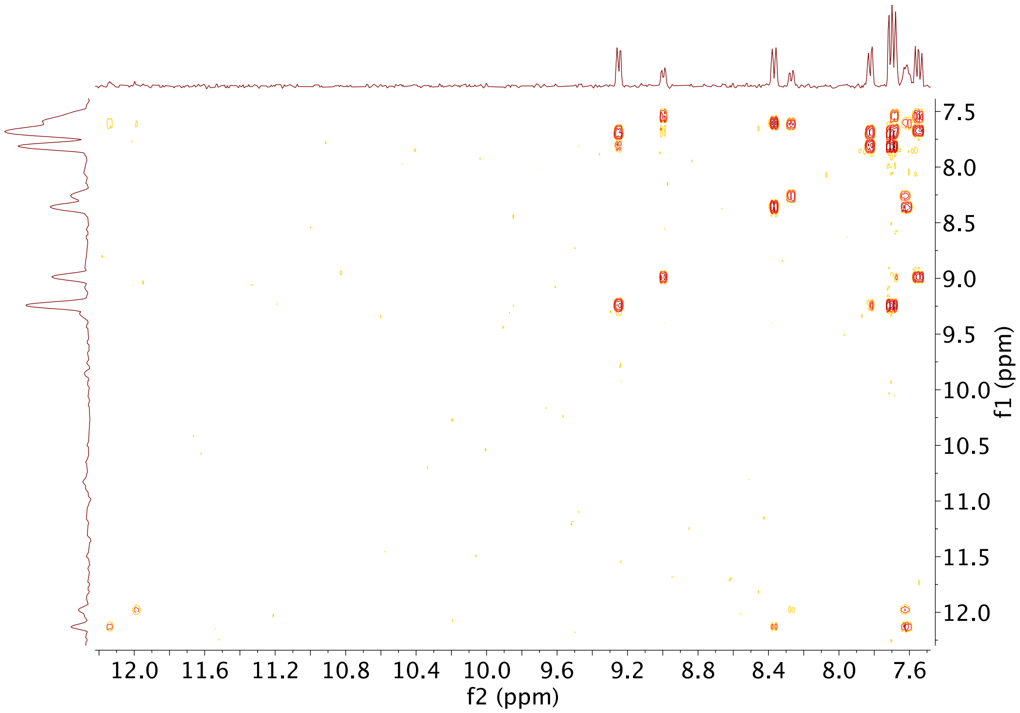

Figure S37. COSY spectrum of {(*o*-chloranil)SbQ_3}$PtCl_2$ (**11**) (400 MHz, CD_2Cl_2)

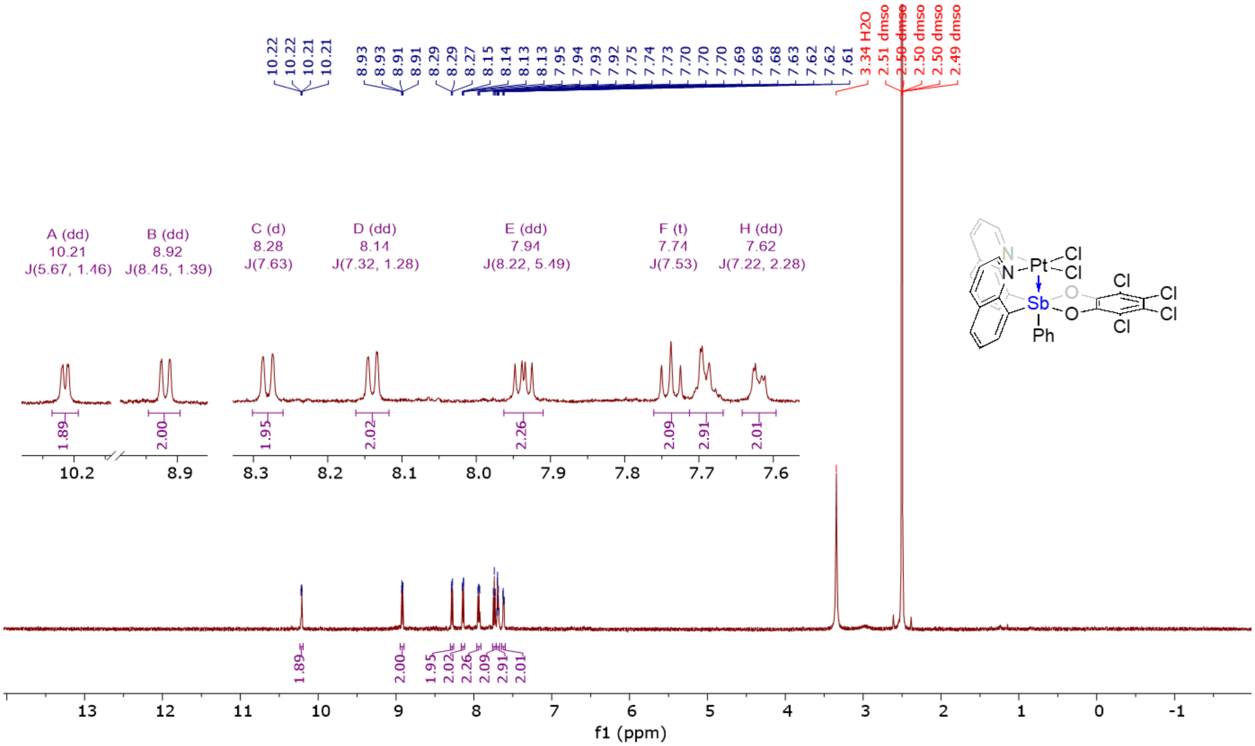

Figure S38. ¹H NMR spectrum of {(*o*-chloranil)SbQ_2Ph}$PtCl_2$ (**12**) (600 MHz, DMSO-d_6). *Note:* water is present in DMSO-d_6 solvent.

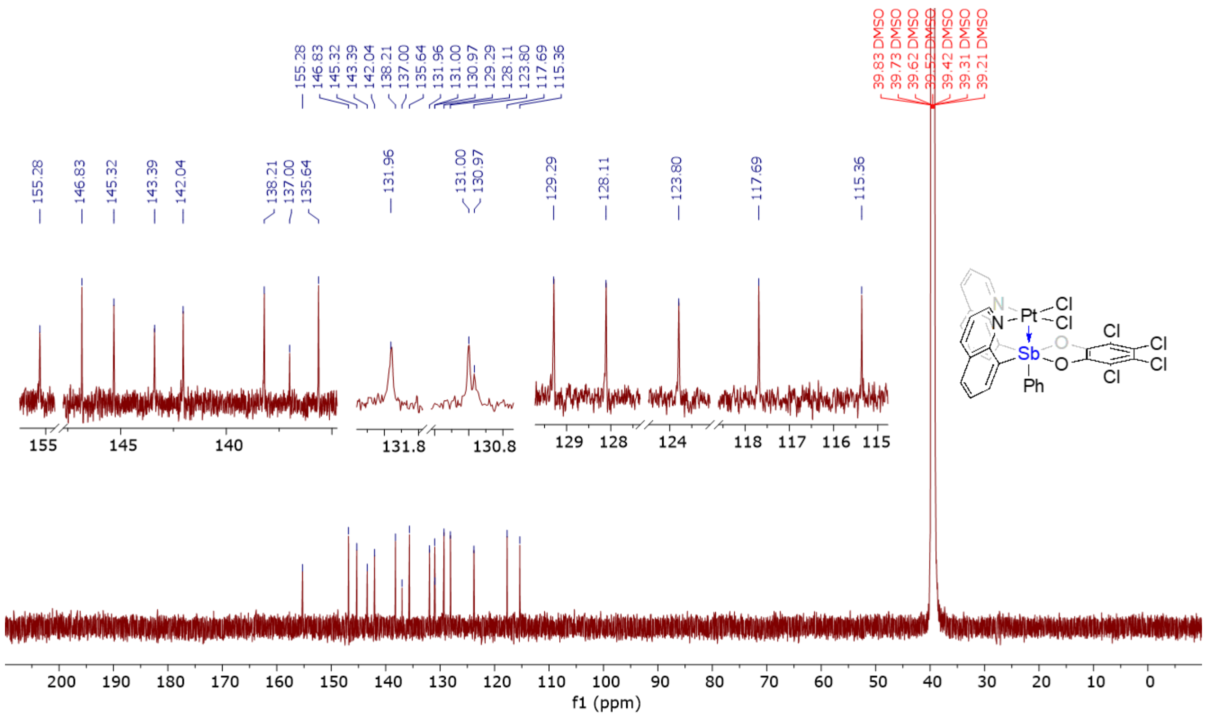

Figure S39. $^{13}C\{^1H\}$ NMR spectrum of {(*o*-chloranil)SbQ_2Ph}PtCl_2 (**12**) (201 MHz, DMSO-d_6).

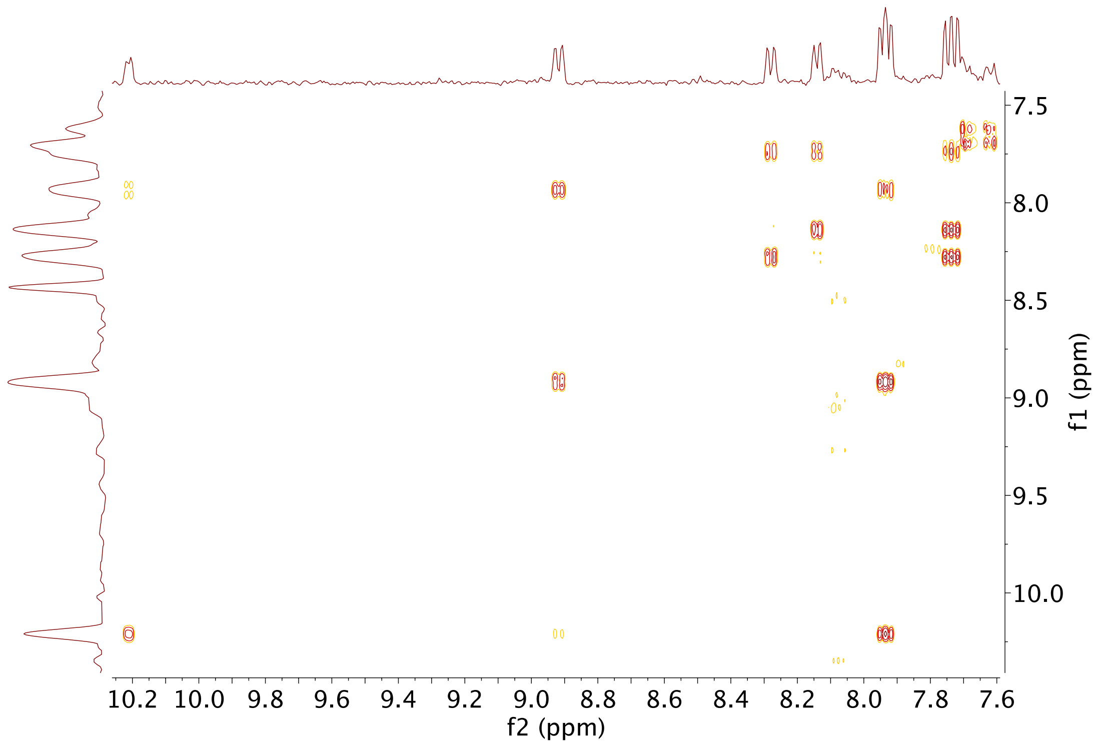

Figure S40. COSY spectrum of {(*o*-chloranil)SbQ_2Ph}PtCl_2 (**12**) (400 MHz, DMSO-d_6).

11. X-Ray Crystal Structure Data

Single crystals of all complexes were coated with Paratone oil and mounted on a MiTeGen MicroLoop. The X-ray intensity data of complexes **3–8**, **10**, **11** and **13** were measured on a Bruker Kappa APEXII Duo system equipped with a graphite monochromator and a Mo K_{α} fine-focus sealed tube (λ = 0.71073 Å), or a Bruker D8 Venture Photon III Kappa four-circle diffractometer system equipped with an Incoatec IμS 3.0 micro-focus sealed X-ray tube (Mo K_{α}, λ = 0.71073 Å) and a HELIOS double bounce multilayer mirror monochromator. The X-ray intensity data of complexes **1**, **2** and **12** were measured on a Bruker Kappa APEXII Duo system equipped with a multi-layer mirror monochromator and a Cu Kα Incoatec Microfocus IμS (λ = 1.54178 Å), or a Bruker D8 Venture Photon III Kappa four-circle diffractometer system (Cu K_{α}, λ = 1.54178 Å).

The frames were integrated with the Bruker SAINT software package[6] using a narrow-frame algorithm. Data were corrected for absorption effects using the Multi-Scan method (SADABS).[6] The structures were solved and refined using the Bruker SHELXTL Software Package[7] within APEX3 or APEX4[6] and OLEX2.[8] All the non-hydrogen atoms in the crystal structures were refined anisotropically. All hydrogen atoms were placed in geometrically calculated positions with $U_{iso} = 1.2U_{equiv}$ of the parent atom ($U_{iso} = 1.5U_{equiv}$ for methyl).

Solvent located in the crystal lattice of complexes **4**, **7**, **8**, **11**, and **12** was severely disordered and could not be adequately modeled with or without restraints. Thus, the structure factors were modified using the PLATON SQUEEZE[9] technique, in order to produce a "solvate-free" structure factor set. A void space of 534 Å^3 containing 147 e$^-$ was found in **4** which likely corresponds to diethyl ether and/or DCE. A void space of 301

Å^3 containing 95 e$^-$ was found in **7**, which corresponds to approximately 1 dichloromethane molecule in the ASU. A void space of 1275 Å^3 containing 494 e$^-$ was found in **8**, which corresponds to approximately 1.5 molecules of dichloromethane per ASU. A void space of 380 Å^3 containing 145 e$^-$ was found in **11**, which corresponds to approximately 2 molecules of chloroform and two molecules of water per ASU. A void space of 611 Å^3 containing 127 e$^-$ was found in **12**, which could correspond to approximately 3 diethyl ether molecules per ASU.

In complexes **3** and **5**, the dichloromethane solvent molecule was disordered over two positions. The relative occupancies of the positions were freely refined with the sum of the occupancies set to one, with SOF of 0.74(2) / 0.26(2) for **3** and 0.54(2) / 0.46(2) for **5**, respectively. In complexes **10** the dichloromethane solvent molecule was disordered over two positions across the symmetry with a SOF of 0.5. In complex **6**, the Pt and Sb atoms were disordered over two positions, and the relative occupancies of the two positions were freely refined with the sum of the occupancies set to one. In complex **8**, one of the quinoline were disordered over two positions across the symmetry. In complex **13**, the *o*-chloranil moiety and diethyl ether solvent molecule were disordered over two positions with SOF of 0.800(12) / 0.200(12) for *o*-chloranil and 0.773(5) / 0.227(5) for the diethyl ether.

Table S1. Crystal structure data table for SbQ_3 (**1**) and SbQ_2Ph (**2**).

	**1**	**2**
CCDC number	2351462	2351463
Empirical formula	$C_{34}H_{26}N_3Sb$	$C_{24}H_{17}N_2Sb$
Formula weight	598.33	455.14
Temperature [K]	100.04	100.00
Wavelength [Å]	1.54178	1.54178
Crystal size [mm^3]	0.063 × 0.088 × 0.162	0.032 × 0.04 × 0.062
Crystal habit	colorless block	colorless plate
Crystal system	monoclinic	monoclinic
Space group	P 2_1/c	P 2_1/c
a [Å]	17.2104(13)	12.1576(6)
b [Å]	17.6422(13)	8.9032(5)
c [Å]	17.2819(13)	18.6627(10)
α [°]	90	90
β [°]	94.135(2)	108.282(3)
γ [°]	90	90
Volume [Å^3]	5233.6(7)	1918.11(18)
Z	8	4
ρ_{calc} [gcm^{-3}]	1.519	1.576
μ [mm^{-1}]	8.574	11.473
F(000)	2416	904
2θ range [°]	5.15 to 140.47	9.98 to 137.38
Index ranges	$-20 \le h \le 20$<br>$-20 \le k \le 21$<br>$-21 \le l \le 21$	$-14 \le h \le 13$<br>$-10 \le k \le 10$<br>$-22 \le l \le 21$
Reflections collected	53761	15185
Independent reflections	9915<br>[R_{int} = 0.0405]	3517<br>[R_{int} = 0.0778]
Data / Restraints / Parameters	9915 / 0 / 687	3517 / 0 / 244
Goodness-of-fit on F^2	1.027	1.081
Final *R* indexes [$I \geq 2\sigma(I)$]	R_1 = 0.0277<br>wR_2 = 0.0755	R_1 = 0.0603<br>wR_2 = 0.1472
Final *R* indexes [all data]	R_1 = 0.0311<br>wR_2 = 0.0790	R_1 = 0.0884<br>wR_2 = 0.1636
Largest peak/hole [eÅ^{-3}]	0.38/−0.66	1.08/−1.42

Table S2. Crystal structure data table for $(SbQ_3)PtCl_2$ (**3**), $(SbQ_2Ph)PtCl_2$ (**4**), and $(SbQ_3)Pt(OAc)_2$ (**5**).

	**3**	**4**	**5**
CCDC number	2351464	2351465	2351466
Empirical formula	$C_{28}H_{20}Cl_4N_3PtSb$	$C_{24}H_{17}Cl_2N_2PtSb$	$C_{32}H_{26}Cl_2N_3O_4PtSb$
Formula weight	857.11	721.13	904.30
Temperature [K]	100(2)	100(2)	100(2)
Wavelength [Å]	0.71073	0.71073	0.71073
Crystal size [mm^3]	0.058 × 0.106 × 0.161	0.066 × 0.066 × 0.088	0.086 × 0.180 × 0.310
Crystal habit	yellow-colorless plate	colorless rod	colorless prism
Crystal system	monoclinic	triclinic	triclinic
Space group	P 2_1/c	P -1	P -1
a [Å]	10.1640(3)	9.188(2)	10.8518(5)
b [Å]	22.5202(7)	10.849(3)	11.3958(4)
c [Å]	11.4858(3)	16.157(4)	12.8320(5)
α [°]	90	105.583(7)	79.3300(10)
β [°]	91.5130(10)	100.202(7)	86.2640(10)
γ [°]	90	98.716(7)	80.8960(10)
Volume [Å^3]	2628.13(13)	1492.8(6)	1538.73(11)
Z	4	2	2
ρ_{calc} [gcm^{-3}]	2.166	1.604	1.952
μ [mm^{-1}]	6.777	5.774	5.634
F(000)	1624	676	868
2θ range [°]	1.99 to 25.71	1.34 to 25.39	1.90 to 28.34
Index ranges	−12 ≤ h ≤ 12<br>−25 ≤ k ≤ 27<br>−12 ≤ l ≤ 14	−11 ≤ h ≤ 11<br>−13 ≤ k ≤ 13<br>−19 ≤ l ≤ 19	−14 ≤ h ≤ 14<br>−15 ≤ k ≤ 14<br>−17 ≤ l ≤ 17
Reflections collected	29331	18384	56291
Independent reflections	4984<br>[R_{int} = 0.0309]	5475<br>[R_{int} = 0.0824]	7646<br>[R_{int} = 0.0475]
Data / Restraints / Parameters	4984 / 0 / 344	5475 / 0 / 271	7646 / 1 / 418
Goodness-of-fit on F^2	1.079	0.924	1.049
Final *R* indexes [$I \geq 2\sigma(I)$]	R_1 = 0.0335<br>wR_2 = 0.0912	R_1 = 0.0428<br>wR_2 = 0.0887	R_1 = 0.0164<br>wR_2 = 0.0405
Final *R* indexes [all data]	R_1 = 0.0355<br>wR_2 = 0.0927	R_1 = 0.0858<br>wR_2 = 0.1020	R_1 = 0.0183<br>wR_2 = 0.0412
Largest peak/hole [eÅ^{-3}]	3.109/−0.993	1.398/−1.040	1.358/−1.063

Table S3. Crystal structure data table for $(SbQ_2Ph)Pt(OAc)_2$ (**6**), $(Cl_2SbQ_3)PtCl_2$ (**7**), $(ClSbQ_3)PtCl_3$ (**8**) and $(ClSbQ_2Ph)PtCl_3$ (**10**).

	**6**	**7**	**8**	**10**
CCDC number	2351468	2351456	2351457	2351458
Empirical formula	$C_{28}H_{23}N_2O_4PtSb$	$C_{27}H_{18}Cl_4N_3PtSb$	$C_{27}H_{18}Cl_4N_3PtSb$	$C_{25}H_{19}Cl_6N_2PtSb$
Formula weight	768.32	843.08	843.08	876.96
Temperature [K]	100(2)	100(2)	100(2)	100(2)
Wavelength [Å]	0.71073	0.71073	0.71073	0.71073
Crystal size [mm^3]	0.061 × 0.064 × 0.073	0.051 × 0.101 × 0.120	0.077 × 0.079 × 0.128	0.079 × 0.106 × 0.304
Crystal habit	yellow plate	colorless plate	colorless-yellow plate	pale yellow rod
Crystal system	triclinic	triclinic	tetragonal	orthorhombic
Space group	P -1	P -1	I 4/m	P n m a
a [Å]	9.3214(4)	9.9563(6)	20.2935(3)	15.8348(18)
b [Å]	10.4338(3)	10.3454(6)	20.2935(3)	14.6902(18)
c [Å]	14.8390(5)	16.4282(9)	14.6408(5)	11.9364(14)
α [°]	72.4690(10)	86.341(2)	90	90
β [°]	84.4140(10)	83.081(2)	90	90
γ [°]	65.3320(10)	62.530(2)	90	90
Volume [Å^3]	1249.92(8)	1490.35(15)	6029.5(3)	2776.6(6)
Z	2	2	8	4
ρ_{calc} [gcm^{-3}]	2.041	1.879	1.858	2.098
μ [mm^{-1}]	6.709	5.973	5.906	6.602
F(000)	732	796	3184	1656
2θ range [°]	2.24 to 26.47	2.50 to 25.27	2.01 to 26.36	2.14 to 28.34
Index ranges	−11 ≤ h ≤ 11<br>−12 ≤ k ≤ 13<br>−18 ≤ l ≤ 18	−11 ≤ h ≤ 11<br>−12 ≤ k ≤ 12<br>−19 ≤ l ≤ 19	−20 ≤ h ≤ 25<br>−25 ≤ k ≤ 24<br>−18 ≤ l ≤ 17	−21 ≤ h ≤ 21<br>−19 ≤ k ≤ 19<br>−15 ≤ l ≤ 15
Reflections collected	43280	37619	19502	25751
Independent reflections	5129 [R_{int} = 0.0397]	5384 [R_{int} = 0.0609]	3207 [R_{int} = 0.0381]	3593 [R_{int} = 0.0616]
Data / Restraints / Parameters	5129 / 0 / 346	5384 / 0 / 325	3207 / 0 / 214	3593 / 0 / 178
Goodness-of-fit on F^2	1.048	1.052	1.092	1.082
Final *R* indexes [$I \geq 2\sigma(I)$]	R_1 = 0.0236<br>wR_2 = 0.0556	R_1 = 0.0355<br>wR_2 = 0.0803	R_1 = 0.0340<br>wR_2 = 0.0850	R_1 = 0.0333<br>wR_2 = 0.0612
Final *R* indexes [all data]	R_1 = 0.0279<br>wR_2 = 0.0583	R_1 = 0.0479<br>wR_2 = 0.0858	R_1 = 0.0499<br>wR_2 = 0.0967	R_1 = 0.0511<br>wR_2 = 0.0677
Largest peak/hole [eÅ^{-3}]	1.213/−0.429	2.698/−1.023	1.165/−0.522	1.952/−1.396

Table S4. Crystal structure data table for {(*o*-chloranil)SbQ$_3$}PtCl$_2$ (**11**), {(*o*-chloranil)SbQ$_2$Ph}PtCl$_2$ (**12**) and {(*o*-chloranil)SbQ$_2$(OMe)}PtCl$_2$ (**13**).

	**11**	**12**	**13**
CCDC number	2351459	2351460	2351461
Empirical formula	$C_{33}H_{18}Cl_6N_3O_2PtSb$	$C_{30}H_{17}Cl_6N_2O_2PtSb$	$C_{29}H_{25}Cl_6N_2O_4PtSb$
Formula weight	1018.04	966.99	995.05
Temperature [K]	100(2)	100(2)	100(2)
Wavelength [Å]	0.71073	1.54178	0.71073
Crystal size [mm^3]	0.078 × 0.136 × 0.184	0.052 × 0.061 × 0.105	0.058 × 0.093 × 0.188
Crystal habit	yellow plate	yellow-colorless needle	yellow-colorless plate
Crystal system	triclinic	monoclinic	monoclinic
Space group	P -1	P 2_1/c	P 2_1/c
a [Å]	10.5445(3)	17.1309(10)	12.5905(4)
b [Å]	10.7368(3)	11.5841(5)	16.9292(5)
c [Å]	16.2891(5)	17.4587(8)	16.2484(5)
α [°]	91.9870(10)	90	90
β [°]	90.4260(10)	101.393(4)	101.6490(10)
γ [°]	97.8100(10)	90	90
Volume [Å^3]	1825.82(9)	3396.3(3)	3391.97(18)
Z	2	4	4
ρ_{calc} [gcm^{-3}]	1.852	1.891	1.949
μ [mm^{-1}]	5.039	18.491	5.425
F(000)	968	1832	1904
2θ range [°]	1.92 to 26.38	2.63 to 68.58	2.23 to 28.30
Index ranges	$-13 \le h \le 13$<br>$-13 \le k \le 13$<br>$-20 \le l \le 20$	$-20 \le h \le 20$<br>$-13 \le k \le 13$<br>$-21 \le l \le 18$	$-16 \le h \le 16$<br>$-22 \le k \le 21$<br>$-21 \le l \le 21$
Reflections collected	45917	31474	53153
Independent reflections	7473<br>[R_{int} = 0.0382]	6227<br>[R_{int} = 0.1768]	8432<br>[R_{int} = 0.0894]
Data / Restraints / Parameters	7473 / 0 / 415	6227 / 0 / 355	8432 / 16 / 372
Goodness-of-fit on F^2	1.077	1.052	1.031
Final *R* indexes [$I \geq 2\sigma(I)$]	R_1 = 0.0355<br>wR_2 = 0.0944	R_1 = 0.0678<br>wR_2 = 0.1541	R_1 = 0.0449<br>wR_2 = 0.1008
Final *R* indexes [all data]	R_1 = 0.0418<br>wR_2 = 0.0998	R_1 = 0.1092<br>wR_2 = 0.1725	R_1 = 0.0813<br>wR_2 = 0.1164
Largest peak/hole [eÅ^{-3}]	2.223/−1.081	1.748/−1.525	1.865/−0.842

12. Absolute Energies for the Calculated Structures

Single points energies are at B2PLYP(D3BJ)/def2-TZVPD level. Energy values are in Hartree.

Complex 7

FINAL SINGLE POINT ENERGY = -3403.282855668618

Zero-point correction = 0.384641

Thermal correction to Energy = 0.432828

Thermal correction to Enthalpy = 0.434010

Thermal correction to Gibbs Free Energy = 0.296085

Complex 8

FINAL SINGLE POINT ENERGY = -3403.272328318600

Zero-point correction = 0.383689

Thermal correction to Energy = 0.432594

Thermal correction to Enthalpy = 0.433775

Thermal correction to Gibbs Free Energy = 0.290773

Complex 9

FINAL SINGLE POINT ENERGY = -3233.643683699304

Zero-point correction = 0.348959

Thermal correction to Energy = 0.393601

Thermal correction to Enthalpy = 0.394782

Thermal correction to Gibbs Free Energy = 0.263015

Complex 10

FINAL SINGLE POINT ENERGY = -3233.647614209871

Zero-point correction = 0.348547

Thermal correction to Energy = 0.393473

Thermal correction to Enthalpy = 0.394654

Thermal correction to Gibbs Free Energy = 0.261082

Complex 11

FINAL SINGLE POINT ENERGY = -4702.388634034842

Zero-point correction = 0.431169

Thermal correction to Energy = 0.490768

Thermal correction to Enthalpy = 0.491949

Thermal correction to Gibbs Free Energy = 0.327954

Complex 12

FINAL SINGLE POINT ENERGY = -4532.751473666562

Zero-point correction = 0.395573

Thermal correction to Energy = 0.452559

Thermal correction to Enthalpy = 0.453741

Thermal correction to Gibbs Free Energy = 0.293520

13. NBO Second Order Perturbation Analysis of the Pt–Sb Bond

Table S5. Second order perturbative Natural Bond Orbital (NBO) analysis of donor-acceptor interaction energy.

Complex	Donor(L) NBO	Acceptor (NL) NBO	E(2) (kcal·mol^{-1})	E(NL) − E(L) (a.u.)	F(L, NL) (a.u.)
**7**	LP(4) Pt1	LV(1) Sb2	111.2	0.18	0.13
**11**	LP(4) Pt1	LV(1) Sb2	71.9	0.25	0.12
**12**	LP(4) Pt1	LV(1) Sb2	14.7	0.21	0.05

14. References

1. Benz, S.; Poblador-Bahamonde, A. I.; Low-Ders, N.; Matile, S., Catalysis with Pnictogen, Chalcogen, and Halogen Bonds. *Angew. Chem. Int. Ed.* **2018,** *57*, 5408-5412. doi: 10.1002/anie.201801452

2. Zhang, L.; Qi, L.; Chen, J.-M.; Dong, W.; Fang, Z.-Y.; Cao, T.-Y.; Li, W.; Wang, L.-J., Preparation of selenyl 1,3-oxazines via PhICl2/Cu2O-promoted aminoselenation of O-homoallyl benzimidates with diselenides. *Chem. Commun.* **2021,** *57*, 12655-12658. doi: 10.1039/D1CC04854F

3. Fulmer, G. R.; Miller, A. J. M.; Sherden, N. H.; Gottlieb, H. E.; Nudelman, A.; Stoltz, B. M.; Bercaw, J. E.; Goldberg, K. I., NMR Chemical Shifts of Trace Impurities: Common Laboratory Solvents, Organics, and Gases in Deuterated Solvents Relevant to the Organometallic Chemist. *Organometallics* **2010,** *29*, 2176-2179. doi: 10.1021/om100106e

4. Deacon, G. B.; Phillips, R. J., Relationships between the carbon-oxygen stretching frequencies of carboxylato complexes and the type of carboxylate coordination. *Coord. Chem. Rev.* **1980,** *33*, 227-250. doi: 10.1016/S0010-8545(00)80455-5

5. Yilmaz, V. T.; Hamamci, S.; Andac, O.; Guven, K., A Three—Dimensional Lead(II) Polymer with Bridging Saccharinate and Unusually Coordinated Acetate Ligands — Synthesis, IR Spectra, and Crystal Structure of [Pb(H2O)(μ-OAc)(μ-sac)]n. *Z. Anorg. Allg. Chem.* **2003,** *629*, 172-176. doi: 10.1002/zaac.200390011

6. Bruker *Saint; SADABS; APEX3; APEX4*, Bruker AXS Inc.: Madison, Wisconsin, USA., 2012.

7. Sheldrick, G. M., SHELXT - integrated space-group and crystal-structure determination. *Acta Crystallogr A Found Adv* **2015,** *A71*, 3-8. doi: 10.1107/s2053273314026370

8. Dolomanov, O.; Bourhis, L.; Gildea, R.; Howard, J.; Puschmann, H., OLEX2: A complete structure solution, refinement and analysis program. *J. Appl. Cryst.* **2009,** *42*, 339-341. doi: 10.1107/S0021889808042726

9. Spek, A. L., PLATON SQUEEZE: a tool for the calculation of the disordered solvent contribution to the calculated structure factors. *Acta Crystallogr. Sect. C: Struct. Chem.* **2015,** *71*, 9-18. doi: 10.1107/s2053229614024929
